# NHC-catalyzed enantioselective synthesis of β-trifluoromethyl-β-hydroxyamides

**DOI:** 10.3762/bjoc.16.129

**Published:** 2020-06-30

**Authors:** Alyn T Davies, Mark D Greenhalgh, Alexandra M Z Slawin, Andrew D Smith

**Affiliations:** 1 EaStCHEM, School of Chemistry, University of St Andrews, North Haugh, St Andrews, Fife KY16 9ST, United Kingdom

**Keywords:** enantioselective catalysis, formal [2 + 2] cycloaddition, N-heterocyclic carbene, ring opening, trifluoromethyl group

## Abstract

The N-heterocyclic carbene (NHC)-catalyzed formal [2 + 2] cycloaddition between α-aroyloxyaldehydes and trifluoroacetophenones, followed by ring opening with an amine or a reducing agent is described. The resulting β-trifluoromethyl-β-hydroxyamide and alcohol products are produced with reasonable diastereocontrol (typically ≈70:30 dr) and excellent enantioselectivity, and they can be isolated in moderate to good yield as a single diastereoisomer.

## Introduction

**Figure 1:**
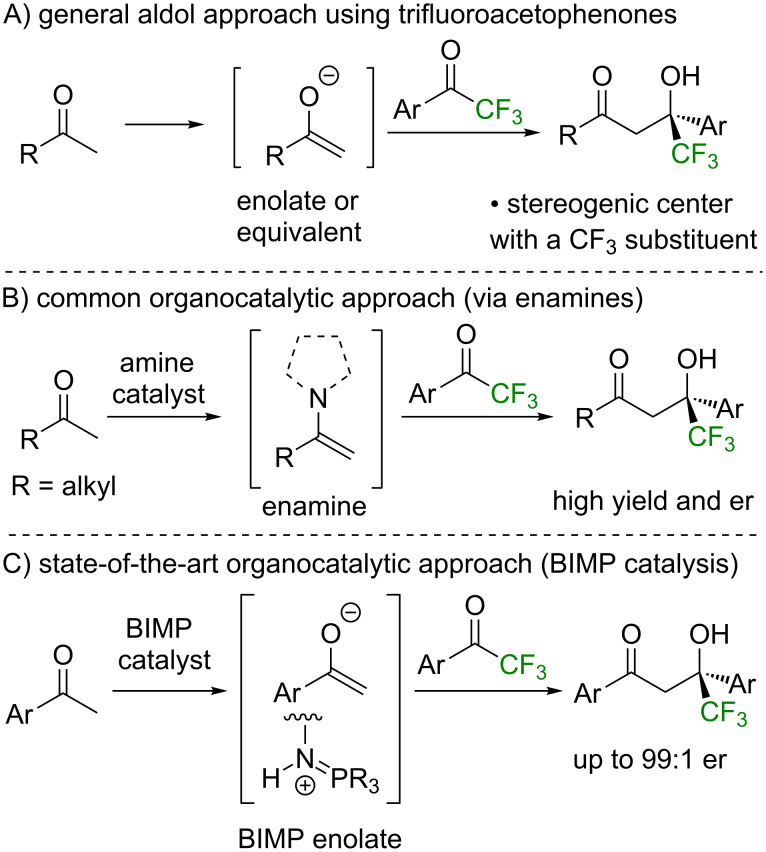
Organocatalytic enantioselective aldol approaches using trifluoroacetophenone derivatives.

The trifluoromethyl unit holds a prominent and privileged position within organic chemistry [1–6]. The incorporation of this motif is widely employed within the pharmaceutical, agrochemical, and materials industries as it can be used strategically to increase lipophilicity as well as to enhance metabolic stability and binding selectivity [7–9]. In this context, the generation of effective and practical methodologies capable of the enantioselective incorporation of the trifluoromethyl unit into organic molecules has been developed widely [10–12], with a series of elegant organocatalytic strategies utilized toward these aims [13]. One general strategy for the organocatalytic construction of stereogenic trifluoromethyl centers is through enantioselective addition of enolates or their equivalents to prochiral trifluoromethyl ketones (Figure 1A). Within this area, a common catalytic approach has utilized aliphatic ketones as enolate equivalents using prolinamide, cinchona, or hybrid catalysts that proceed via enamine intermediates (Figure 1B) [14–19]. The state of the art within this area has been recently demonstrated by Dixon and co-workers, who showed that bifunctional BIMP catalysts could promote the enantioselective addition of typically recalcitrant aryl ketones to trifluoromethyl ketones (Figure 1C) [20].

**Figure 2:**
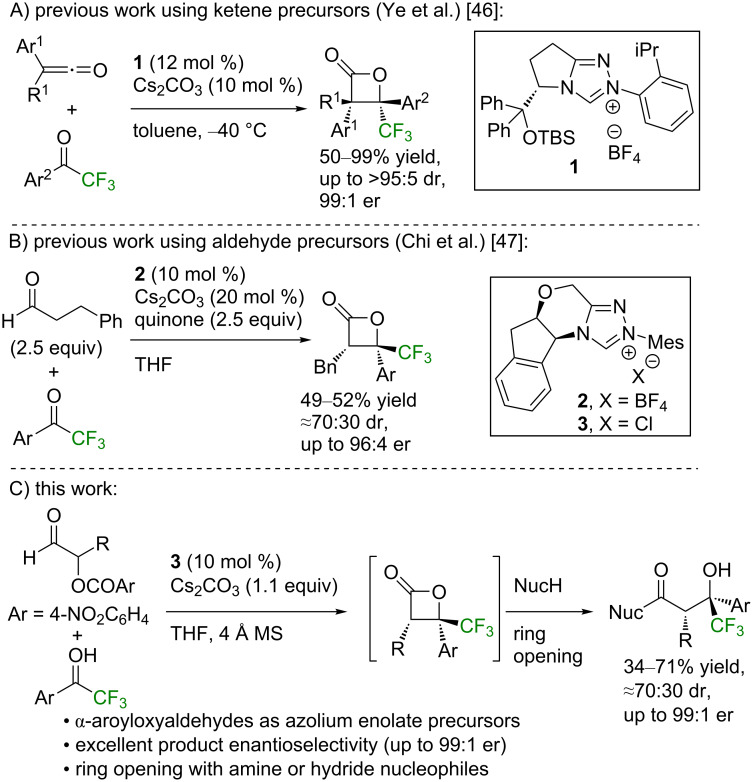
NHC-catalyzed approaches to β-lactones using trifluoroacetophenone derivatives.

Over the last twenty years, NHCs have been widely exploited as highly efficient organocatalysts that have found use in numerous applications and were the subject of many extensive reviews [21–26]. Among the most common reactive intermediates generated using NHCs, the azolium enolate has been widely used [27–28]. Methods to generate azolium enolates from a number of precursors have been reported, including the use of ketenes [29–30], α-functionalized aldehydes [31–35], enals [36–38], aryl esters [39–42], or aldehydes [43–45] in the presence of an oxidant. As representative examples of the use of azolium enolates in reactions with trifluoroacetophenone derivatives, Ye and co-workers have shown that using disubstituted ketenes as azolium enolate precursors and NHC precatalyst **1** allowed access to trifluoromethyl-substituted β-lactone products in a high yield, diastereo-, and enantioselectivity (Figure 2A) [46]. Chi and co-workers have reported limited examples of oxidative [2 + 2] cycloadditions using hydrocinnamaldehyde as an azolium enolate precursor and the NHC precatalyst **2**, giving β-lactone products in high enantioselectivity but requiring superstoichiometric quantities of quinone as an oxidant (Figure 2B) [47]. In our previous work, we have developed α-aroyloxyaldehydes as reactive, bench-stable precursors of azolium enolates [48–53], which can be synthesized in a single step from the desired aldehyde [54]. We have previously applied these precursors in the NHC-catalyzed formal [2 + 2] cycloadditions between α-aroyloxyaldehydes and perfluoroalkyl-substituted ketones using NHC precatalyst **3** to produce polyfluorinated oxetanes and β-perfluoroalkyl-β-hydroxyamides after derivatization [55]. In this paper, this formal [2 + 2] protocol is extended to the use of trifluoroacetophenones to develop an enantioselective route to β-trifluoromethyl-β-hydroxy carboxylic acid derivatives (Figure 2C).

## Results and Discussion

### Optimization

Optimization of the NHC-catalyzed formal [2 + 2] cycloaddition using the α-aroyloxyaldehyde **4** and trifluoromethylacetophenone (**5**) as reactants began using the NHC precatalyst **3**, triethylamine as the base, and THF as the solvent (Table 1, entry 1). A moderate conversion (48%, as determined by NMR analysis) to the desired β-lactone product **6** as a 70:30 mixture of diastereoisomers was observed, which served as a basis for further optimization. Variation of the base showed caesium carbonate to be significantly more effective than the organic bases examined, giving 88% conversion to product **6** (Table 1, entries 2 and 3). Trialling a number of alternative solvents, including dichloromethane, diethyl ether, and toluene showed no improvement upon the yield obtained with THF (Table 1, entries 4–6). Unfortunately, attempts to isolate the desired β-lactone product **6** were unsuccessful, with **6** being unstable to chromatographic purification under a variety of different conditions. As such, following the NHC-catalyzed formal [2 + 2] cycloaddition in THF, a subsequent ring opening step with allylamine was investigated. Although the diastereomeric ratio of the resultant crude reaction mixture was 75:25 after chromatographic purification, as seen by NMR analysis, the corresponding β-trifluoromethyl-β-hydroxyamide **7** was isolated as a single diastereoisomer in 57% yield and >99:1 er (Table 1, entry 7).

**Table 1:** Optimization of the NHC-catalyzed formal [2 + 2] cycoaddition.^a^

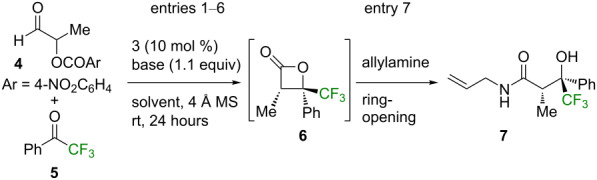					

entry	solvent	base	dr^b^	yield (%)	er^c^

1	THF	Et_3_N	70:30	48^d^	–
2	THF	iPr_2_NEt	70:30	50^d^	–
3	THF	Cs_2_CO_3_	75:25	88^d^	–
4	CH_2_Cl_2_	Cs_2_CO_3_	80:20	61^d^	–
5	Et_2_O	Cs_2_CO_3_	70:30	58^d^	–
6	toluene	Cs_2_CO_3_	70:30	55^d^	–
7	THF	Cs_2_CO_3_	75:25	57^e^	>99:1

^a^The data contained in this Table has previously been published as part of our previous communication in this area [55]. ^b^Determined by ^1^H NMR spectroscopic analysis of the crude reaction product mixture. ^c^er of the major diastereoisomer (>95:5 dr), determined by GC analysis using a chiral support. ^d^Combined yield of both diastereoisomers of **6**, determined by the analysis of the crude ^1^H NMR spectra with reference to 2,5-dimethylfuran as an internal standard. ^e^Isolated yield of product **7** (>95:5 dr).

### Scope and limitations

**Scheme 1:**
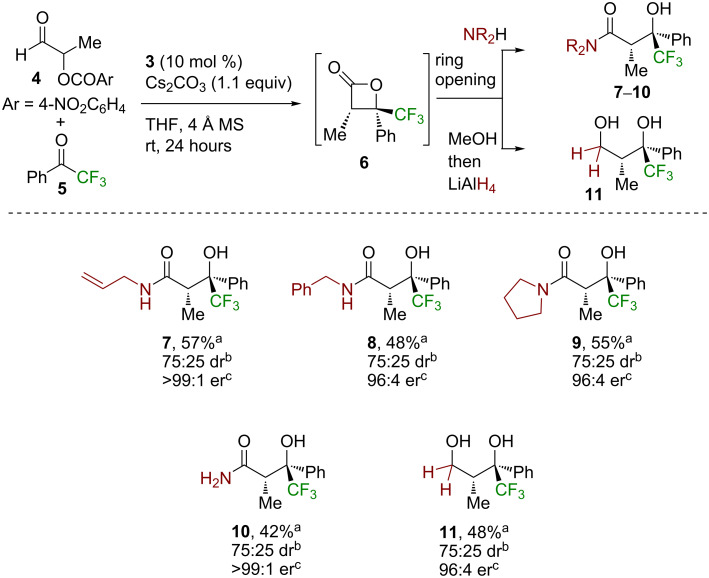
Reaction scope with respect to the nucleophile. ^a^Isolated yield of the product in >95:5 dr. ^b^Determined by ^1^H NMR spectroscopic analysis of the crude reaction product mixture. ^c^Determined by HPLC analysis or GC analysis using a chiral support.

Having optimized this process with a model system, further work probed the scope and limitations of this process through a sequential variation of the nucleophile used for the ring opening procedure, aryl and heteroaryl substitution of the trifluoromethylacetophenone, as well as variation of the α-aroyloxyaldehyde. Using α-aroyloxyaldehyde **4** and trifluoromethylacetophenone (**5**) as reactants, the variation of the nucleophile showed that a range of amine-based nucleophiles was readily tolerated in this process (Scheme 1). The use of benzylamine, pyrrolidine, and ammonia all gave the corresponding β-trifluoromethyl-β-hydroxyamide product in ≈75:25 dr, with purification giving **8**–**10**, respectively, as a single diastereoisomer in 42–55% yield and excellent enantioselectivity. Using methanol as a nucleophile gave the corresponding ester, however, this product proved unstable to purification. Generation of the ester in situ, followed by subsequent reduction with LiAlH_4_, gave the diol **11** in 48% isolated yield as a single diastereoisomer in 96:4 er.

**Scheme 2:**
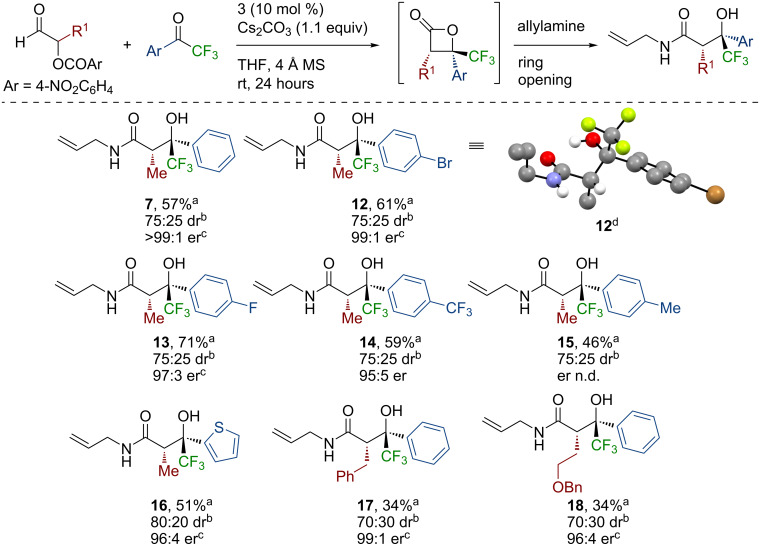
Reaction scope with respect to the trifluoroacetophenone derivative and α-aroyloxyaldehyde. ^a^Isolated yield of the product in >95:5 dr. ^b^Determined by ^1^H NMR spectroscopic analysis of the crude reaction product mixture. ^c^er of the major diastereoisomer (>95:5 dr), determined by HPLC analysis or GC analysis using a chiral support. ^d^Molecular representation of the X-ray crystal structure of **12**. The unit cell of **12** contained two molecules of **12**, with only one shown for clarity.

Subsequent variation of the electronic nature of the aromatic substituent within the trifluoroacetophenone component, followed by a ring opening with allylamine, showed that the introduction of electron-withdrawing groups (positive Hammett sigma constants) [56], such as *p*-bromo, *p*-fluoro, and *p*-trifluoromethyl groups, respectively, were well tolerated, giving a consistent diastereoselectivity of ≈75:25 dr (Scheme 2). Following chromatographic purification, the desired products **12**–**14**, respectively, were isolated in 59–71% yield as single diastereoisomers with excellent enantioselectivity (95:5 to >99:1 er). The relative and absolute configuration of (2*S*,3*S*)-β-trifluoromethyl-β-hydroxyamide **12** was confirmed by single crystal X-ray crystallographic analysis, with all other products in this series assigned by analogy to **12** [57]. The introduction of an electron-donating *p*-tolyl substituent was also tolerated in the system, providing the corresponding β-trifluoromethyl-β-hydroxyamide **15** in a moderate yield of 46%, although the er of **15** could not be determined by either GC or HPLC analysis. The trifluoroacetophenone derivatives with stronger electron-donating *p*-methoxy and *p*-dimethylamino substituents proved unreactive, consistent with the expected lower electrophilicity. A heterocyclic substituent could also be incorporated using this methodology, with thiophene-substituted β-trifluoromethyl-β-hydroxyamide **16** being produced in 80:20 dr, with purification giving **16** in 51% yield as a single diastereoisomer and 96:4 er. The substrate scope was further investigated by variation of the α-aroyloxyaldehyde component of the reaction. Functionalized α-aroyloxyaldehydes containing a C2-benzyl and a C2-benzyloxy derivative were tested, producing the corresponding β-trifluoromethyl-β-hydroxyamides **17** and **18**, respectively, in ≈70:30 dr after a ring opening with allylamine. Purification gave **17** and **18**, respectively, in a moderate yield as single diastereoisomers and with a good enantioselectivity.

**Scheme 3:**
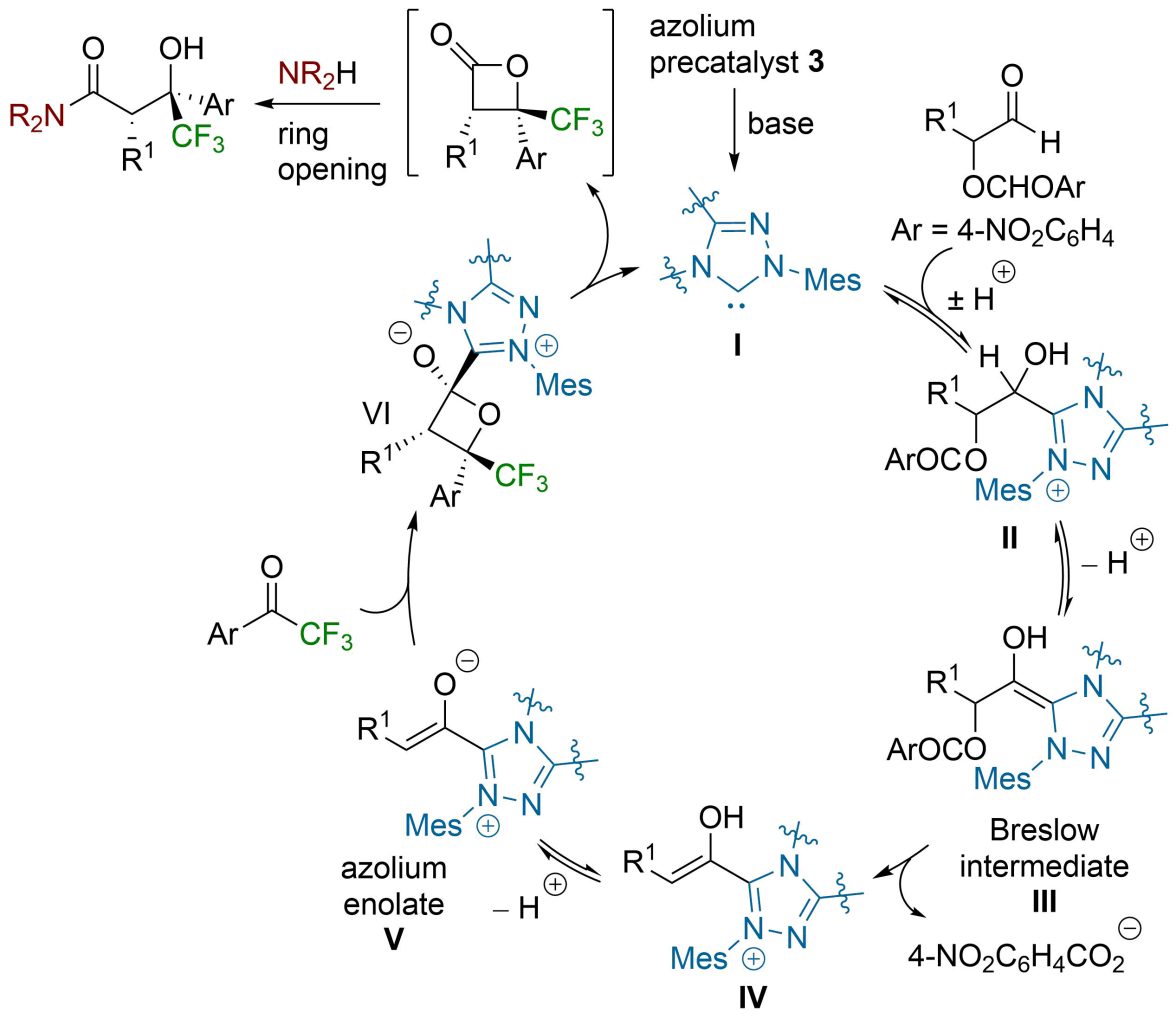
Proposed mechanism.

The mechanism of this NHC redox process is believed to proceed through the following mechanism (Scheme 3): After deprotonation of the triazolium salt precatalyst **3** [58], reversible addition of the free NHC **I** to the aldehyde leads to adduct **II** [59]. A subsequent deprotonation allows access to Breslow intermediate **III**, which can eliminate *para*-nitrobenzoate to leave azolium enol **IV**. Deprotonation gives azolium enolate intermediate **V**, which undergoes a formal [2 + 2] cycloaddition with the trifluoroacetophenone derivative to give **VI** [60]. Elimination of the NHC catalyst completes the catalytic cycle and provides the corresponding β-lactone product. Subsequent ring opening with a suitable nucleophilic amine leads to the isolable β-trifluoromethyl-β-hydroxyamide (Scheme 3).

## Conclusion

In this paper, we showed that azolium enolates generated from α-aroyloxyaldehydes can undergo NHC-catalyzed formal [2 + 2] cycloadditions with trifluoroacetophenone derivatives. Although the β-lactone products proved unstable to chromatographic purification, ring opening with amine nucleophiles allowed access to β-trifluoromethyl-β-hydroxyamides in moderate to good yield as single diastereoisomers in excellent er following purification [61].

## Supporting Information

File 1 Experimental procedures, product characterization data (mp, NMR, IR, HRMS, [α]_D_, HPLC), and spectra (^1^H, ^13^C, and ^19^F NMR, HPLC).

File 2 Crystallographic details for **12**.
