# Supplementary material for: NHC-catalyzed enantioselective synthesis of β-trifluoromethyl-β-hydroxyamides
Source: Beilstein J Org Chem. 2020 Jun 30;16:1572–8. doi: 10.3762/bjoc.16.129 (PMC7356285; doi:10.3762/bjoc.16.129)
Supplement: File 1 — Experimental procedures, product characterization data (mp, NMR, IR, HRMS, [α]D, HPLC), and spectra (1H, 13C, and 19F NMR, HPLC). [file Beilstein_J_Org_Chem-16-1572-s001.pdf]

BEILSTEIN JOURNAL OF ORGANIC CHEMISTRY

Supporting Information

for

NHC-catalyzed enantioselective synthesis of β-trifluoromethyl-β-hydroxyamides

Alyn T. Davies, Mark D. Greenhalgh, Alexandra M. Z. Slawin and Andrew D. Smith

Beilstein J. Org. Chem. **2020,** *16,* 1572–1578. doi:10.3762/bjoc.16.129

Experimental procedures, product characterization data (mp, NMR, IR, HRMS, $[\alpha]_D$, HPLC), and spectra (^{1}H, ^{13}C, and ^{19}F NMR, HPLC)

License and Terms: This is a supporting information file under the terms of the Creative Commons Attribution License (http://creativecommons.org/licenses/by/4.0). Please note that the reuse, redistribution and reproduction in particular requires that the authors and source are credited.
The license is subject to the *Beilstein Journal of Organic Chemistry* terms and conditions: (https://www.beilstein-journals.org/bjoc)

General Experimental	S2
NHC-Catalyzed Formal [2+2] Cycloadditions	S4
NMR Traces	S13
GC and HPLC Traces	S46
References	S56

General Experimental

Reactions carried out under a nitrogen atmosphere were done so using standard vacuum line techniques. All reaction glassware was flame-dried and cooled under vacuum prior to use.

Anhydrous THF were obtained and purified by an alumina column (Mbraun SPS-800). Anhydrous methanol was obtained by distillation over calcium hydride. All commercial reagents were used as supplied without further purification.

Analytical thin layer chromatography was performed on pre-coated aluminium plates (Kieselgel 60 F_{254} silica). TLC visualisation was carried out with ultraviolet light (254 nm), followed by staining with a 1% aqueous $KMnO_4$ solution. Flash column chromatography was performed on Kieselgel 60 silica in the solvent system stated.

^{1}H, ^{13}C and ^{19}F nuclear magnetic resonance (NMR) spectra were acquired on either a Bruker Avance II 400 (^{1}H 400 MHz; ^{13}C 101 MHz; ^{19}F 376 MHz), Bruker Avance 500 (^{1}H 500 MHz; ^{13}C 126 MHz; ^{19}F 471 MHz) or a Bruker Avance III 500 (^{1}H 500 MHz; ^{13}C 126 MHz; ^{19}F 471 MHz) spectrometer at ambient temperature in the deuterated solvent stated. All chemical shifts are quoted in parts per million (ppm) relative to the residual solvent. All coupling constants, *J*, are quoted in Hz and determined by analysis using MestReNova v9.0.1 software. Multiplicities are indicated by: s (singlet), d (doublet), t (triplet) and q (quartet), and combinations of these. The abbreviation Ar is used to denote aromatic.

Infrared spectra were recorded on a Shimadzu IRAffinity-1 Fourier transform IR spectrophotometer fitted with a Specac Quest ATR accessory (diamond puck). Spectra were recorded of either thin films or solids, with characteristic absorption wavenumbers (ν_{max}) reported in cm^{-1}.

Melting points were recorded on an Electrothermal 9100 melting point apparatus and are uncorrected.

HPLC analyses were obtained on a Shimadzu HPLC consisting of a Shimadzu DGU-20A5 degasser, Shimadzu LC-20AT liquid chromatograph, Shimadzu SIL-20AT auto sampler, Shimadzu CBM-20A communications bus module, Shimadzu SPD-M20A diode array detector, Shimadzu CTO-20A column oven and a Shimadzu FRC-10A fraction collector. Analysis was performed using Shimadzu LabSolutions v5.42 software and separation was achieved using the column described.

GC analyses were obtained on a Shimadzu GC consisting of a Shimadzu AOC-20i auto injector and a Shimadzu GC-2025 gas chromatograph. Analysis was performed using Shimadzu GCsolution v2.41 software and separation was achieved using the column described.

Mass spectrometry (*m/z*) data were acquired by electrospray ionisation (ESI) or atmospheric pressure chemical ionisation (APCI) at the EPSRC UK National Mass Spectrometry Facility at Swansea University. Low resolution NSI MS was carried out on a Micromass Quattro II spectrometer and high resolution NSI MS on a Thermofisher LTQ Orbitrap XL spectrometer.

Optical rotations were measured on a PerkinElmer Precisely/Model-341 polarimeter operating at the sodium D line with a 100 mm path cell.

Starting Materials

NHC precatalyst **3**[1] and α-aroyloxyaldehydes **4**, **S1** and **S2**[2] were synthesised as previously reported. Trifluoromethylketones **5** and **S3–S7** were purchased from the suppliers stated below.

α-Aroyloxyaldehyde substrates

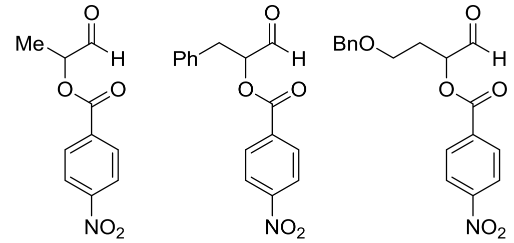

4 **S1** **S2**

Trifluoromethylketone substrates

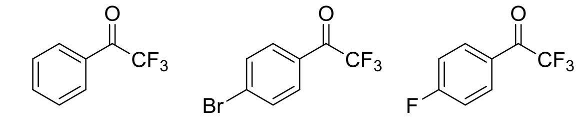

**5**	**S3**	**S4**
CAS: 434-45-7	CAS: 16184-89-7	CAS: 655-32-3
Sigma Aldrich	Apollo Scientific	Fluorochem

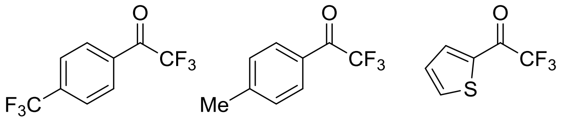

**S5**	**S6**	**S7**
CAS: 74853-66-0	CAS: 394-59-2	CAS: 651-70-7
Apollo Scientific	Fluorochem	Sigma Aldrich

NHC Catalyzed Formal [2+2] Cycloadditions

General procedure A: NHC Catalyzed Formal [2+2] Cycloadditions with trifluoromethylketones, followed by Amine Ring Opening

Following a similar procedure to that described previously,[3] α-aroyloxyaldehyde (1.5 eq.), trifluoromethylketone (1.0 eq.) and precatalyst **3** (0.1 eq.) were dissolved in anh. THF (0.05 M) in a flame-dried flask containing molecular sieves (4Å) under an N_2 atmosphere at room temperature. Caesium carbonate (1.1 eq.) was added and the reaction was allowed to stir for 24 h. The mixture was diluted with Et_2O, washed with sat. aq. NH_4Cl and sat. aq. $NaHCO_3$. The organic layer was dried (Na_2SO_4), filtered and concentrated *in vacuo* to give a residue which was dissolved in anh. THF (0.1 M). The specified amine nucleophile (5.0 eq.) and NEt_3 (1.1 eq.) were added and the solution was allowed to stir for 24 h. The mixture was diluted with Et_2O, washed with sat. aq. NH_4Cl and brine. The organic layer was dried (Na_2SO_4), filtered and concentrated *in vacuo* to leave the crude product, which was purified by column chromatography on silica.

*Racemic samples of all products were synthesised by the same method using a racemic sample of precatalyst **3**.*

(2*S*,3*S*)-*N*-Allyl-4,4,4-trifluoro-3-hydroxy-2-methyl-3-phenylbutanamide (**7**)

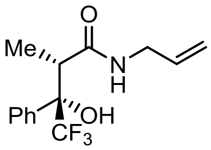

The preparation, characterization data and chiral GC analysis, along with the corresponding NMR and GC traces, for compound **7** can be found in our previous publication.[3]

(2*S*,3*S*)-*N*-Benzyl-4,4,4-trifluoro-3-hydroxy-2-methyl-3-phenylbutanamide (**8**)

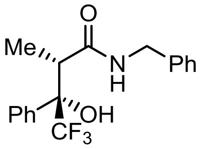

Following general procedure **A**, 1-oxopropan-2-yl 4-nitrobenzoate **4** (335 mg, 1.50 mmol), trifluoroacetophenone **5** (140 µL, 1.00 mmol), precatalyst **3** (37 mg, 0.10 mmol), caesium carbonate (358 mg, 1.10 mmol) and THF (20 mL) for 24h; followed by benzylamine (0.55 mL, 5.0 mmol), NEt_3 (139 µL, 1.00 mmol) and THF (10 mL) for a further 24 h gave the crude product (75:25 dr), which

was purified by column chromatography on silica (20% Et_2O in hexane) to give (2*S*,3*S*)-*N*-benzyl-4,4,4-trifluoro-3-hydroxy-2-methyl-3-phenylbutanamide **8** as a pale yellow solid (single diastereoisomer, 161 mg, 0.477 mmol, 48%).

mp 102–104 °C; $[\alpha]_D^{20}$ +38.0 (*c* 0.5, $CHCl_3$); **Chiral HPLC analysis**; Chiralcel OD-H (95:5 hexane : 2-propanol, flow rate 1 $mLmin^{-1}$, 211 nm, 30 °C) t_R minor (2*R*,3*R*): 23.0 min, t_R major (2*S*,3*S*): 24.8 min, 96:4 er; **^{1}H NMR** (400 MHz, $CDCl_3$) δ_H: 1.00 (3H, d, *J* 7.0, CH_3CH), 2.96 (1H, q, *J* 7.0, CH_3CH), 4.33–4.66 (2H, m, $NHCH_2$), 6.37 (1H, t, *J* 5.7, N*H*), 6.74 (1H, s, O*H*), 7.27–7.44 (8H, m, Ar*H*), 7.56 (1H, d, *J* 7.3, C(3)ArC(2,6)*H*); **^{13}C{^{1}H} NMR** (101 MHz, $CDCl_3$) δ_C: 13.9 (CH_3CH), 41.8 (CH_3CH), 43.7 ($NHCH_2$), 78.4 (q, *J* 27.4, CCF_3), 125.8 (q, *J* 288.4, CF_3), 126.0 (C(3)Ar*C*(2,6)), 127.9 (CH_2Ar*C*(2,6)H), 127.9 (Ar*C*H), 128.4 (2 × Ar*C*H), 128.5 (Ar*C*H), 128.9 (2 × Ar*C*H), 136.0 (C(3)Ar*C*(1)), 137.1 (CH_2Ar*C*(1)), 175.8 (*C*=O); **^{19}F{^{1}H} NMR** (376 MHz, $CDCl_3$) δ_F: −76.7 (CF_3); **IR** ν_{max} (film)/cm^{-1}: 3397 (O–H), 1647 (C=O); **HRMS** ($APCI^+$) $C_{18}H_{19}F_3O_2N$ ($[M+H]^+$), found 338.1362, requires 338.1362 (−0.1 ppm).

(2*S*,3*S*)-4,4,4-Trifluoro-3-hydroxy-2-methyl-3-phenyl-1-(pyrrolidin-1-yl)butan-1-one (**9**)

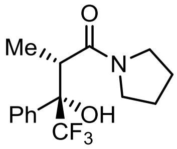

Following general procedure **A**, 1-oxopropan-2-yl 4-nitrobenzoate **4** (335 mg, 1.50 mmol), trifluoroacetophenone **5** (140 µL, 1.00 mmol), precatalyst **3** (37 mg, 0.10 mmol), caesium carbonate (358 mg, 1.10 mmol) and THF (20 mL) for 24h; followed by pyrrolidine (0.42 mL, 5.0 mmol), NEt_3 (139 µL, 1.00 mmol) and THF (10 mL) for a further 24 h gave the crude product (75:25 dr), which was purified by column chromatography on silica (30% Et_2O in hexane) to give (2*S*,3*S*)-4,4,4-trifluoro-3-hydroxy-2-methyl-3-phenyl-1-(pyrrolidin-1-yl)butan-1-one **9** as a colourless crystalline solid (single diastereoisomer, 166 mg, 0.551 mmol, 55%).

mp 122–125 °C; $[\alpha]_D^{20}$ +45.1 (*c* 0.5, $CHCl_3$); **Chiral HPLC analysis**; Chiralcel OD-H (90:10 hexane : 2-propanol, flow rate 1 $mLmin^{-1}$, 211 nm, 30 °C) t_R minor (2*R*,3*R*): 5.3 min, t_R major (2*S*,3*S*): 5.9 min, 96:4 er; **^{1}H NMR** (500 MHz, $CDCl_3$) δ_H: 0.92 (3H, d, *J* 7.0, CH_3CH), 1.88–1.97 (2H, m, NCH_2CH_2), 1.97–2.12 (2H, m, NCH_2CH_2), 3.29 (1H, q, *J* 7.0, CH_3CH), 3.46–3.62 (3H, m, NCH_2 and NCH_aH_b), 3.68 (1H, dt, *J* 9.8, 7.0, NCH_aH_b), 7.33–7.44 (4H, m, ArC(2,3,5,6)*H*), 7.54–7.64 (2H, m, ArC(4)*H* and O*H*); **^{13}C{^{1}H} NMR** (101 MHz, $CDCl_3$) δ_C: 13.2 (CH_3CH), 24.3 (NCH_2CH_2), 26.0 (NCH_2CH_2), 38.0 (CH_3CH), 46.0 (NCH_2), 46.8 (NCH_2), 78.2 (q, *J* 27.2, CCF_3), 126.0 (q, *J* 288.7, CF_3), 126.2 (Ar*C*(2,6)H), 128.28 (Ar*C*(3,5)H), 128.34 (Ar*C*(4)H), 136.5 (Ar*C*(1)), 174.3 (*C*=O);

^{19}F{^{1}H} NMR (471 MHz, $CDCl_3$) δ_F: –77.1 (CF_3); **IR** ν_{max} (film)/cm^{-1}: 3063 (O–H), 1617 (C=O); **HRMS** (APCI$^+$) $C_{15}H_{19}F_3O_2N$ ([M+H]$^+$), found 302.1362, requires 302.1362 (–0.1 ppm).

(2*S*,3*S*)-4,4,4-Trifluoro-3-hydroxy-2-methyl-3-phenylbutanamide (10)

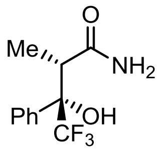

Following general procedure **A**, 1-oxopropan-2-yl 4-nitrobenzoate **4** (335 mg, 1.50 mmol), trifluoroacetophenone **5** (140 µL, 1.00 mmol), precatalyst **3** (37 mg, 0.10 mmol), caesium carbonate (358 mg, 1.10 mmol) and THF (20 mL) for 24h; followed by ammonia (7 M in MeOH, 0.71 mL, 5.00 mmol), NEt_3 (139 µL, 1.00 mmol) and THF (10 mL) for a further 24 h gave the crude product (75:25 dr), which was purified by column chromatography on silica (40% Et_2O in hexane) to give (2*S*,3*S*)-4,4,4-trifluoro-3-hydroxy-2-methyl-3-phenylbutanamide **10** as a colourless solid (single diastereoisomer, 104 mg, 0.419 mmol, 42%).

mp 149–151 °C; $[\alpha]_D^{20}$ +17.4 (*c* 0.5, $CHCl_3$); **Chiral GC analysis** Restek Rt®bDEXcst (length: 30 m, thickness: 0.250 mm, film thickness: 0.25 µm), carrier gas: He, linear velocity: 60 cm/sec, temperature: 160 °C, t_R minor (2*R*,3*R*) 44.0 min, t_R major (2*S*,3*S*) 45.6 min, > 99:1 er; **^{1}H NMR** (400 MHz, $CDCl_3$) δ_H: 1.00 (3H, d, *J* 7.0, CH_3CH), 3.01 (1H q, *J* 7.0, CH_3C*H*), 5.96 (1H, s, N*H*), 6.14 (1H, s, N*H*), 6.53 (1H, s, O*H*), 7.32–7.46 (3H, m, ArC(3,4,5)*H*), 7.56 (2H, dd, *J* 7.3, 1.8, ArC(2,6)*H*); **^{13}C{^{1}H} NMR** (101 MHz, $CDCl_3$) δ_C: 13.8 (*C*H_3CH), 40.9 (CH_3*C*H), 78.3 (q, *J* 27.4, *C*CF_3), 125.7 (q, *J* 288.2, *C*F_3), 126.0 (Ar*C*(2,6)H), 128.4 (Ar*C*(3,5)H), 128.5 (Ar*C*(4)H), 135.9 (Ar*C*(1)), 178.7 (*C*=O); **^{19}F{^{1}H} NMR** (376 MHz, $CDCl_3$) δ_F: –76.9 (CF_3); **IR** ν_{max} (film)/cm^{-1}: 3200 (O–H), 1668 (C=O); **HRMS** (APCI$^+$) $C_{11}H_{13}F_3O_2N$ ([M+H]$^+$), found 248.0893, requires 248.0893 (+0.0 ppm).

(2*R*,3*S*)-4,4,4-Trifluoro-2-methyl-3-phenylbutane-1,3-diol (11)

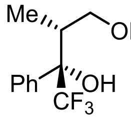

Following a *modification of general procedure **A***, 1-oxopropan-2-yl 4-nitrobenzoate **4** (335 mg, 1.50 mmol), trifluoroacetophenone **5** (140 µL, 1.00 mmol), precatalyst **3** (37 mg, 0.10 mmol), caesium carbonate (358 mg, 1.10 mmol) and THF (20 mL) for 24h. The crude product was then dissolved in MeOH (10 mL), DMAP (24 mg, 0.20 mmol) was added, and the reaction allowed to stir for 24 h. The solvent was removed *in vacuo* and the crude product was treated with lithium aluminium hydride (2 M

in PhMe, 2 mL, 4.00 mmol) under a N_2 atmosphere, and allowed to stir for a further 24 h. The reaction was quenched by slow addition of 1 M KOH, and the mixture extracted using EtOAc (3 × 20 mL). The combined organic layers were washed with brine, dried (Na_2SO_4), filtered and concentrated *in vacuo* to give the crude product (75:25 dr), which was purified by column chromatography on silica (15% Et_2O in hexane) to give (2*R*,3*S*)-4,4,4-trifluoro-2-methyl-3-phenylbutane-1,3-diol **11** as an orange oil (single diastereoisomer, 113 mg, 0.483 mmol, 48%).

$[\alpha]_D^{20}$ −39.4 (*c* 0.5, $CHCl_3$); **Chiral GC analysis** Agilent Cyclosil-B (length: 30 m, thickness: 0.250 mm, film thickness: 0.25 μm), carrier gas: He, linear velocity: 30 cm/sec, temperature: 135 °C, t_R minor (2*S*,3*R*) 50.8 min, t_R major (2*R*,3*S*) 51.8 min, 96:4 er; **^{1}H NMR** (400 MHz, $CDCl_3$) δ_H: 0.90 (3H, d, *J* 7.2, C*H*$_3$CH), 2.11 (1H, s, CH_2O*H*), 2.35–2.53 (1H, m, CH_3C*H*), 3.83 (1H, dt, *J* 10.8, 1.8, C*H*$_a$H$_b$OH), 4.41 (1H, d, *J* 10.7, CH$_a$*H*$_b$OH), 5.20 (1H, s, F_3CCO*H*), 7.31–7.43 (3H, m, ArC(3,4,5)*H*), 7.55 (2H, d, *J* 7.7, ArC(2,6)*H*); **^{13}C{^{1}H} NMR** (101 MHz, $CDCl_3$) δ_C: 12.6 (*C*H$_3$CH), 37.8 (CH_3*C*H), 66.0 (*C*H$_2$OH), 80.4 (q, *J* 27.1, *C*CF$_3$), 125.6 (Ar*C*(2,6)H), 126.2 (q, *J* 287.9, *C*F$_3$), 128.0 (Ar*C*(4)H), 128.2 (Ar*C*(3,5)H), 138.2 (Ar*C*(1)); **^{19}F{^{1}H} NMR** (376 MHz, $CDCl_3$) δ_F: −74.7 (C*F*$_3$); **IR** ν_{max} (film)/cm^{-1}: 3372 (O–H); **HRMS** (APCI$^+$) $C_{11}H_{14}F_3O_2$ ([M+H]$^+$), found 235.0934, requires 235.0940 (−2.7 ppm).

(2*S*,3*S*)-*N*-Allyl-3-(4-bromophenyl)-4,4,4-trifluoro-3-hydroxy-2-methylbutanamide (**12**)

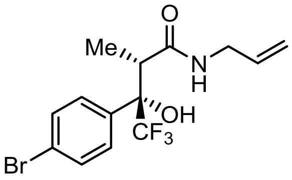

Following general procedure **A**, 1-oxopropan-2-yl 4-nitrobenzoate **4** (167 mg, 0.750 mmol), 1-(4-bromophenyl)-2,2,2-trifluoroethan-1-one **S3** (127 mg, 0.500 mmol), precatalyst **3** (18 mg, 50 μmol), caesium carbonate (179 mg, 0.550 mmol) and THF (10 mL) for 24h; followed by allylamine (188 μL, 2.50 mmol), NEt_3 (70 μL, 0.50 mmol) and THF (5 mL) for a further 24 h gave the crude product (75:25 dr), which was purified by column chromatography on silica (30% Et_2O in hexane) to give (2*S*,3*S*)-*N*-allyl-3-(4-bromophenyl)-4,4,4-trifluoro-3-hydroxy-2-methylbutanamide **12** as a colourless crystalline solid (single diastereoisomer, 111 mg, 0.304 mmol, 61%).

mp 107–108 °C; $[\alpha]_D^{20}$ +22.6 (*c* 0.5, $CHCl_3$); **Chiral GC analysis** Agilent Cyclosil-B (length: 30 m, thickness: 0.250 mm, film thickness: 0.25 μm), carrier gas: He, linear velocity: 60 cm/sec, temperature: 190 °C, t_R minor (2*R*,3*R*) 29.9 min, t_R major (2*S*,3*S*) 30.6 min, 99:1 er; **^{1}H NMR** (400 MHz, $CDCl_3$) δ_H: 0.97 (3H, d, *J* 7.0, C*H*$_3$), 2.88 (1H, q, *J* 7.0, CH_3C*H*), 3.74–4.12 (2H, m, NHC*H*$_2$), 5.06–5.37 (2H, m, HC=C*H*$_2$), 5.85 (1H, ddt, *J* 17.2, 10.2, 5.7, *H*C=CH$_2$), 6.10 (1H, t, *J* 5.8, N*H*), 6.75

(1H, s, O*H*), 7.43 (2H, d, *J* 8.4, ArC(3,5)*H*), 7.53 (2H, d, *J* 8.8, ArC(2,6)*H*); **^{13}C{^{1}H} NMR** (126 MHz, $CDCl_3$) δ_C: 13.9 (*C*H_3), 41.6 (CH_3*C*H), 42.0 (NH*C*H_2), 78.2 (q, *J* 27.6, *C*CF_3), 117.4 (HC=*C*H_2), 122.9 (Ar*C*(4)), 125.5 (q, *J* 288.4, *C*F_3), 127.9 (Ar*C*(3,5)H), 131.6 (Ar*C*(2,6)H), 132.9 (H*C*=CH_2), 135.2 (Ar*C*(1)), 175.5 (*C*=O); **^{19}F{^{1}H} NMR** (376 MHz, $CDCl_3$) δ_F: −76.9 (C*F*$_3$); **IR** ν_{max} (film)/cm^{-1}: 3302 (O–H), 1634 (C=O); **HRMS** (ESI$^+$) $C_{14}H_{16}{}^{79}BrF_3NO_2$ ([M+H]$^+$), found 366.0314, requires 366.0311 (+0.8 ppm).

(2*S*,3*S*)-*N*-Allyl-4,4,4-trifluoro-3-(4-fluorophenyl)-3-hydroxy-2-methylbutanamide (**13**)

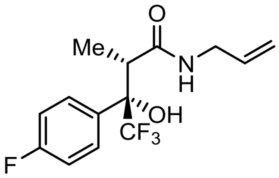

Following general procedure **A**, 1-oxopropan-2-yl 4-nitrobenzoate **4** (167 mg, 0.750 mmol), 2,2,2-trifluoro-1-(4-fluorophenyl)ethan-1-one **S4** (70 µL, 0.50 mmol), precatalyst **3** (18 mg, 50 µmol), caesium carbonate (179 mg, 0.550 mmol) and THF (10 mL) for 24h; followed by allylamine (188 µL, 2.50 mmol), NEt_3 (70 µL, 0.50 mmol) and THF (5 mL) for a further 24 h gave the crude product (75:25 dr), which was purified by column chromatography on silica (30% Et_2O in hexane) to give (2*S*,3*S*)-*N*-allyl-4,4,4-trifluoro-3-(4-fluorophenyl)-3-hydroxy-2-methylbutanamide **13** as a colourless crystalline solid (single diastereoisomer, 109 mg, 0.357 mmol, 71%).

mp 90–92 °C; $[\alpha]_D^{20}$ +19.2 (*c* 0.5, $CHCl_3$); **Chiral GC analysis** Agilent Cyclosil-B (length: 30 m, thickness: 0.250 mm, film thickness: 0.25 µm), carrier gas: He, linear velocity: 60 cm/sec, temperature: 157 °C, t_R minor (2*R*,3*R*) 40.1 min, t_R major (2*S*,3*S*) 41.1 min, 97:3 er; **^{1}H NMR** (400 MHz, $CDCl_3$) δ_H: 0.97 (3H, d, *J* 7.0, C*H*$_3$CH), 2.89 (1H, q, *J* 7.0, CH_3C*H*), 5.12–5.32 (2H, m, HC=C*H*$_2$), 5.85 (1H, ddt, *J* 17.2, 10.2, 5.7, *H*C=CH_2), 6.05 (1H, s, N*H*), 6.71 (1H, s, O*H*), 7.08 (2H, dd, *J* 9.1, 8.4, ArC(3,5)*H*), 7.54 (2H, dd, *J* 8.6, 5.3, ArC(2,6)*H*); **^{13}C{^{1}H} NMR** (101 MHz, $CDCl_3$) δ_C: 13.8 (*C*H_3CH), 41.8 (CH_3*C*H), 42.0 (NH*C*H_2), 78.1 (q, *J* 27.6, *C*CF_3), 115.3 (d, *J* 21.5, Ar*C*(3,5)H), 117.4 (HC=*C*H_2), 125.6 (q, *J* 288.3, *C*F_3), 128.0 (d, *J* 8.1, Ar*C*(2,6)H), 131.8 (d, *J* 3.2, Ar*C*(1)), 133.0 (H*C*=CH_2), 162.7 (d, *J* 247.7, Ar*C*(4)), 175.5 (*C*=O); **^{19}F{^{1}H} NMR** (377 MHz, $CDCl_3$) δ_F: −113.7 (ArC(4)*F*), −77.1 (C*F*$_3$); **IR** ν_{max} (film)/cm^{-1}: 3306 (O–H), 1622 (C=O); **HRMS** (ESI$^+$) $C_{14}H_{15}F_4NO_2Na$ ([M+Na]$^+$), found 328.0932, requires 328.0931 (+0.3 ppm).

(2*S*,3*S*)-*N*-allyl-4,4,4-trifluoro-3-hydroxy-2-methyl-3-(4-(trifluoromethyl)phenyl)butanamide (14)

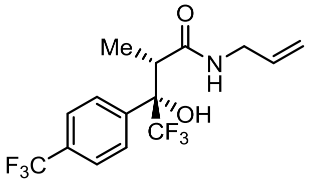

Following general procedure **A**, 1-oxopropan-2-yl 4-nitrobenzoate **4** (167 mg, 0.750 mmol), 2,2,2-trifluoro-1-(4-(trifluoromethyl)phenyl)ethan-1-one **S5** (84 µL, 0.50 mmol), precatalyst **3** (18 mg, 50 µmol), caesium carbonate (179 mg, 0.550 mmol) and THF (10 mL) for 24h; followed by allylamine (188 µL, 2.50 mmol), NEt_3 (70 µL, 0.50 mmol) and THF (5 mL) for a further 24 h gave the crude product (75:25 dr), which was purified by column chromatography on silica (30% Et_2O in hexane) to give (2*S*,3*S*)-*N*-allyl-4,4,4-trifluoro-3-hydroxy-2-methyl-3-(4-(trifluoromethyl)phenyl)butanamide **14** as a colourless crystalline solid (single diastereoisomer, 105 mg, 0.295 mmol, 59%).

mp 86–87 °C; $[\alpha]_D^{20}$ +14.6 (*c* 0.5, $CHCl_3$); **Chiral GC analysis** Agilent Cyclosil-B (length: 30 m, thickness: 0.250 mm, film thickness: 0.25 µm), carrier gas: He, linear velocity: 40 cm/sec, temperature: 165 °C, t_R minor (2*R*,3*R*) 40.0 min, t_R major (2*S*,3*S*) 40.7 min, 95:5 er; **^{1}H NMR** (500 MHz, $CDCl_3$) δ_H: 0.97 (3H, d, *J* 7.0, C*H*$_3$CH), 2.94 (1H, q, *J* 7.0, CH_3C*H*), 3.85–4.09 (2H, m, NHC*H*$_2$), 5.15–5.34 (2H, m, HC=C*H*$_2$), 5.86 (1H, ddt, *J* 17.2, 10.3, 5.7, *H*C=CH_2), 6.08 (1H, t, *J* 5.9, N*H*), 6.83 (1H, s, O*H*), 7.59–7.80 (4H, m, Ar*H*); **^{13}C{^{1}H} NMR** (126 MHz, $CDCl_3$) δ_C: 13.9 (*C*H_3CH), 41.6 (CH_3C*H*), 42.0 (NH*C*H_2), 78.3 (q, *J* 27.6, *C*CF_3), 117.4 (HC=*C*H_2), 123.9 (q, *J* 272.2, *C*F_3), 125.4 (q, *J* 3.3, Ar*C*(3,5)H), 125.5 (q, *J* 288.6, *C*F_3), 126.6 (Ar*C*(2,6)H), 130.8 (q, *J* 32.6, Ar*C*(4)), 132.9 (H*C*=CH_2), 140.1 (Ar*C*(1)), 175.3 (*C*=O); **^{19}F{^{1}H} NMR** (471 MHz, $CDCl_3$) δ_F: −76.8 (C*F*$_3$), −62.7 (ArC*F*$_3$); **IR** ν_{max} (film)/cm^{-1}: 3323 (O–H), 1641 (C=O); **HRMS** (APCI$^+$) $C_{15}H_{16}F_6O_2N$ ($[M+H]^+$), found 356.1079, requires 356.1080 (−0.2 ppm).

(2*S*,3*S*)-*N*-Allyl-4,4,4-trifluoro-3-hydroxy-2-methyl-3-(*p*-tolyl)butanamide (15)

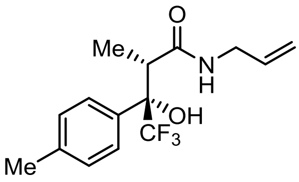

Following general procedure **A**, 1-oxopropan-2-yl 4-nitrobenzoate **4** (335 mg, 1.50 mmol), 2,2,2-trifluoro-1-(p-tolyl)ethan-1-one **S6** (152 µL, 1.00 mmol), precatalyst **3** (37 mg, 0.10 mmol), caesium carbonate (358 mg, 1.10 mmol) and THF (20 mL) for 24h; followed by allylamine (0.38 mL, 5.0 mmol), NEt_3 (139 µL, 1.00 mmol) and THF (10 mL) for a further 24 h gave the crude product (75:25

dr), which was purified by column chromatography on silica (25% Et_2O in hexane) to give (2*S*,3*S*)-*N*-allyl-4,4,4-trifluoro-3-hydroxy-2-methyl-3-(*p*-tolyl)butanamide **15** as a colourless crystalline solid (single diastereoisomer, 139 mg, 0.460 mmol, 46%).

mp 108–110 °C; $[\alpha]_D^{20}$ +23.0 (*c* 0.5, $CHCl_3$); **^{1}H NMR** (500 MHz, $CDCl_3$) δ_H: 0.98 (3H, d, *J* 7.0, C*H*$_3$CH), 2.36 (3H, s, ArC(4)C*H*$_3$), 2.90 (1H, q, *J* 7.0, CH_3C*H*), 3.78–4.10 (2H, m, NHC*H*$_2$), 5.07–5.38 (2H, m, HC=C*H*$_2$), 5.86 (1H, ddt, *J* 16.3, 10.8, 5.7, *H*C=CH_2), 6.04 (1H, t, *J* 6.0, N*H*), 6.58 (1H, s, O*H*), 7.20 (2H, d, *J* 8.0, ArC(3,5)*H*), 7.43 (2H, d, *J* 7.9, ArC(2,6)*H*); **^{19}F{^{1}H} NMR** (471 MHz, $CDCl_3$) δ_F: −77.0; **^{13}C{^{1}H} NMR** (101 MHz, $CDCl_3$) δ_C: 13.9 (*C*H$_3$CH), 21.2 (ArC(4)*C*H$_3$), 41.9 (CH_3*C*H), 42.0 (NH*C*H$_2$), 78.3 (q, *J* 27.3, *C*CF$_3$), 117.2 (HC=*C*H$_2$), 125.8 (q, *J* 288.3, *C*F$_3$), 125.9 (Ar*C*(2,6)H), 129.1 (Ar*C*(3,5)H), 133.0 (Ar*C*(1)), 133.1 (H*C*=CH_2), 138.2 (Ar*C*(4)), 175.8 (*C*=O); **IR** ν_{max} (film)/cm^{-1}: 3356 (O–H), 1647 (C=O); **HRMS** (APCI$^+$) $C_{15}H_{19}F_3O_2N$ ([M+H]$^+$), found 302.1365, requires 302.1362 (+0.9 ppm).

No separation of the enantiomers could be obtained using Chiral GC or HPLC.

(2*S*,3*R*)-*N*-Allyl-4,4,4-trifluoro-3-hydroxy-2-methyl-3-(thiophen-2-yl)butanamide (16)

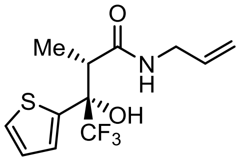

Following general procedure **A**, 1-oxopropan-2-yl 4-nitrobenzoate **4** (167 mg, 0.750 mmol), 2,2,2-trifluoro-1-(thiophen-2-yl)ethan-1-one **S7** (64 µL, 0.50 mmol), precatalyst **3** (18 mg, 50 µmol), caesium carbonate (179 mg, 0.550 mmol) and THF (10 mL) for 24h; followed by allylamine (188 µL, 2.50 mmol), NEt_3 (70 µL, 0.50 mmol) and THF (5 mL) for a further 24 h gave the crude product (80:20 dr), which was purified by column chromatography on silica (20% Et_2O in hexane) to give (2*S*,3*R*)-*N*-allyl-4,4,4-trifluoro-3-hydroxy-2-methyl-3-(thiophen-2-yl)butanamide **16** as a colourless crystalline solid (single diastereoisomer, 76 mg, 0.26 mmol, 51%).

mp 108–109 °C; $[\alpha]_D^{20}$ +14.6 (*c* 0.5, $CHCl_3$); **Chiral GC analysis** Agilent Cyclosil-B (length: 30 m, thickness: 0.250 mm, film thickness: 0.25 µm), carrier gas: He, linear velocity: 60 cm/sec, temperature: 160 °C, t_R minor (2*R*,3*R*) 33.6 min, t_R major (2*S*,3*S*) 34.5 min, 96:4 er; **^{1}H NMR** (400 MHz, $CDCl_3$) δ_H: 1.09 (3H, d, *J* 7.0, C*H*$_3$), 2.81 (1H, q, *J* 7.0, CH_3C*H*), 3.78–4.12 (2H, m, NHC*H*$_2$), 5.11–5.34 (2H, m, HC=C*H*$_2$), 5.84 (1H, ddt, *J* 17.2, 10.3, 5.8, *H*C=CH_2), 5.97 (1H, s, N*H*), 6.84 (1H, s, O*H*), 7.00–7.07 (2H, m, ArC(3,5)*H*), 7.30–7.37 (1H, m, ArC(4)*H*); **^{13}C{^{1}H} NMR** (126 MHz, $CDCl_3$) δ_C: 14.0 (*C*H$_3$), 42.0 (NH*C*H$_2$), 43.1 (CH_3*C*H), 78.4 (q, *J* 28.9, *C*CF$_3$), 117.4 (HC=*C*H$_2$), 124.5 (Ar*C*(3)H or Ar*C*(5)H), 125.2 (q, *J* 287.8, *C*F$_3$), 125.9 (Ar*C*(4)H), 127.2 (Ar*C*(3)H or Ar*C*(5)H),

133.0 (H*C*=CH_2), 140.3 (Ar*C*(2)), 175.4 (*C*=O); **^{19}F{^{1}H} NMR** (376 MHz, $CDCl_3$) δ_F: −78.5 (C*F*$_3$); **IR** ν_{max} (film)/cm^{-1}: 3341 (O–H), 1638 (C=O); **HRMS** (ESI^+) $C_{12}H_{15}F_3NO_2S$ ($[M+H]^+$), found 294.0773, requires 294.0770 (+1.0 ppm).

(2*S*,3*S*)-*N*-Allyl-2-benzyl-4,4,4-trifluoro-3-hydroxy-3-phenylbutanamide (**17**)

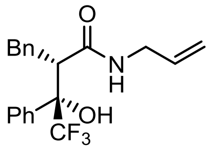

Following general procedure **A**, 1-oxo-3-phenylpropan-2-yl 4-nitrobenzoate **S1** (449 mg, 1.50 mmol), trifluoroacetophenone **5** (140 µL, 1.00 mmol), precatalyst **3** (37 mg, 0.10 mmol), caesium carbonate (358 mg, 1.10 mmol) and THF (20 mL) for 24h; followed by allylamine (0.38 mL, 5.0 mmol), NEt_3 (139 µL, 1.00 mmol) and THF (10 mL) for a further 24 h gave the crude product (70:30 dr), which was purified by column chromatography on silica (20% Et_2O in hexane) to give (2*S*,3*S*)-*N*-allyl-2-benzyl-4,4,4-trifluoro-3-hydroxy-3-phenylbutanamide **17** as a colourless crystalline solid (single diastereoisomer, 125 mg, 0.343 mmol, 34%).

mp 126–128 °C; $[\alpha]_D^{20}$ +31.1 (*c* 0.5, $CHCl_3$); **Chiral HPLC analysis**; Chiralcel OD-H (95:5 hexane : 2-propanol, flow rate 1 mLmin^{-1}, 211 nm, 30 °C) t_R minor (2*R*,3*R*): 5.7 min, t_R major (2*S*,3*S*): 7.1 min, 99:1 er; **^{1}H NMR** (400 MHz, $CDCl_3$) δ_H: 2.39 (1H, dd, *J* 12.9, 2.8, CH_aH$_b$CH), 2.80–2.90 (1H, m, CH$_a$$H_b$CH), 2.94 (1H, dd, *J* 11.9, 2.8, CH_2C*H*), 3.72 (2H, tt, *J* 5.6, 1.4, NHC*H*$_2$), 4.89 (1H, dd, *J* 17.1, 1.4, HC=CH_{trans}H), 5.00 (1H, dd, *J* 10.3, 1.3, HC=CH_{cis}H), 5.35 (1H, t, *J* 4.7, N*H*), 5.49 (1H, ddt, *J* 17.2, 10.3, 5.8, *H*C=CH_2), 6.69 (1H, s, O*H*), 6.96–7.03 (2H, m, Ar*H*), 7.15–7.26 (3H, m, Ar*H*), 7.38–7.44 (1H, m, Ar*H*), 7.45–7.53 (2H, m, Ar*H*), 7.68 (2H, d, *J* 7.6, C(3)ArC(2,6)*H*); **^{13}C{^{1}H} NMR** (126 MHz, $CDCl_3$) δ_C: 34.3 (*C*H$_2$CH), 41.8 (NH*C*H$_2$), 51.3 (CH_2*C*H), 78.4 (q, *J* 27.3, *C*CF$_3$), 117.1 (HC=*C*H$_2$), 125.5 (q, *J* 288.6, *C*F$_3$), 125.9 (C(3)Ar*C*(2,6)H), 126.8 (Ar*C*H), 128.63 (2 × Ar*C*H), 128.65 (2 × Ar*C*H), 128.68 (Ar*C*H), 128.8 (2 × Ar*C*H), 132.8 (H*C*=CH_2), 136.2 (C(3)Ar*C*(1)), 138.2 (CH_2Ar*C*(1)), 173.6 (*C*=O); **^{19}F{^{1}H} NMR** (376 MHz, $CDCl_3$) δ_F: −76.5 (C*F*$_3$); **IR** ν_{max} (film)/cm^{-1}: 3312 (O–H), 1628 (C=O); **HRMS** (ESI^+) $C_{20}H_{21}F_3NO_2$ ($[M+H]^+$), found 364.1518, requires 364.1519 (−0.2 ppm).

(2*S*,3*S*)-*N*-Allyl-2-(2-(benzyloxy)ethyl)-4,4,4-trifluoro-3-hydroxy-3-phenylbutanamide (**18**)

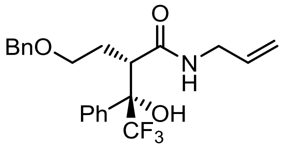

Following general procedure **A**, 4-(benzyloxy)-1-oxobutan-2-yl 4-nitrobenzoate **S2** (258 mg, 0.750 mmol), trifluoroacetophenone **5** (70 µL, 0.50 mmol), precatalyst **3** (18 mg, 50 µmol), caesium carbonate (179 mg, 0.550 mmol) and THF (10 mL) for 24h; followed by allylamine (188 µL, 2.50 mmol), NEt_3 (70 µL, 0.50 mmol) and THF (5 mL) for a further 24 h gave the crude product (70:30 dr), which was purified by column chromatography on silica (20% Et_2O in hexane) to give (2*S*,3*S*)-*N*-allyl-2-(2-(benzyloxy)ethyl)-4,4,4-trifluoro-3-hydroxy-3-phenylbutanamide **18** as a colourless oil (single diastereoisomer, 70 mg, 0.17 mmol, 34%).

$[\alpha]_D^{20}$ +26.5 (*c* 0.5, $CHCl_3$); **Chiral HPLC analysis**; Chiralcel OD-H (95:5 hexane : 2-propanol, flow rate 1 mLmin^{-1}, 211 nm, 30 °C) t_R minor (2*R*,3*R*): 6.8 min, t_R major (2*S*,3*S*): 10.0 min, 96:4 er; **^{1}H NMR** (400 MHz, $CDCl_3$) δ_H: 1.42–1.52 (1H, m, CH_aH$_b$CH), 1.79 (1H, ddt, *J* 14.7, 11.4, 3.4, CH$_a$$H_b$CH), 3.12 (1H, dd, *J* 11.4, 3.4, CH$_2$C*H*), 3.26–3.40 (2H, m, OCH_2CH$_2$), 3.65–3.76 (1H, m, NHCH_aH$_b$), 3.89–3.99 (1H, m, NHCH$_a$$H_b$), 4.29 (1H, d, *J* 11.9, OCH_aH$_b$Ar), 4.44 (1H, d, *J* 11.9, OCH$_a$$H_b$Ar), 5.13–5.29 (2H, m, HC=CH_2), 5.77 (1H, ddt, *J* 17.1, 10.2, 5.8, *H*C=CH$_2$), 5.90 (1H, t, *J* 6.0, N*H*), 6.65 (1H, s, O*H*), 7.24–7.45 (8H, m, Ar*H*), 7.57 (2H, d, *J* 7.5, C(3)ArC(2,6)*H*); **^{13}C{^{1}H} NMR** (126 MHz, $CDCl_3$) δ_C: 27.8 (*C*H$_2$CH), 42.0 (NH*C*H), 44.1 (CH$_2$*C*H), 66.5 (O*C*H$_2$CH$_2$), 72.8 (O*C*H$_2$Ar), 78.5 (q, *J* 27.4, *C*CF$_3$), 117.4 (HC=*C*H$_2$), 125.6 (q, *J* 288.5, *C*F$_3$), 126.1 (C(3)Ar*C*(2,6)H), 127.9 (2 × Ar*C*H), 128.0 (Ar*C*H), 128.4 (2 × Ar*C*H), 128.5 (Ar*C*H), 128.6 (2 × Ar*C*H), 133.2 (H*C*=CH$_2$), 136.1 (C(3)Ar*C*(1)), 138.0 (OCH$_2$Ar*C*(1)), 174.4 (*C*=O); **^{19}F{^{1}H} NMR** (376 MHz, $CDCl_3$) δ_F: −76.7 (C*F*$_3$); **IR** ν_{max} (film)/cm^{-1}: 3319 (O–H), 1628 (C=O); **HRMS** (APCI$^+$) $C_{22}H_{25}F_3O_3N$ ([M+H]$^+$), found 408.1781, requires 408.1781 (+0.0 ppm).

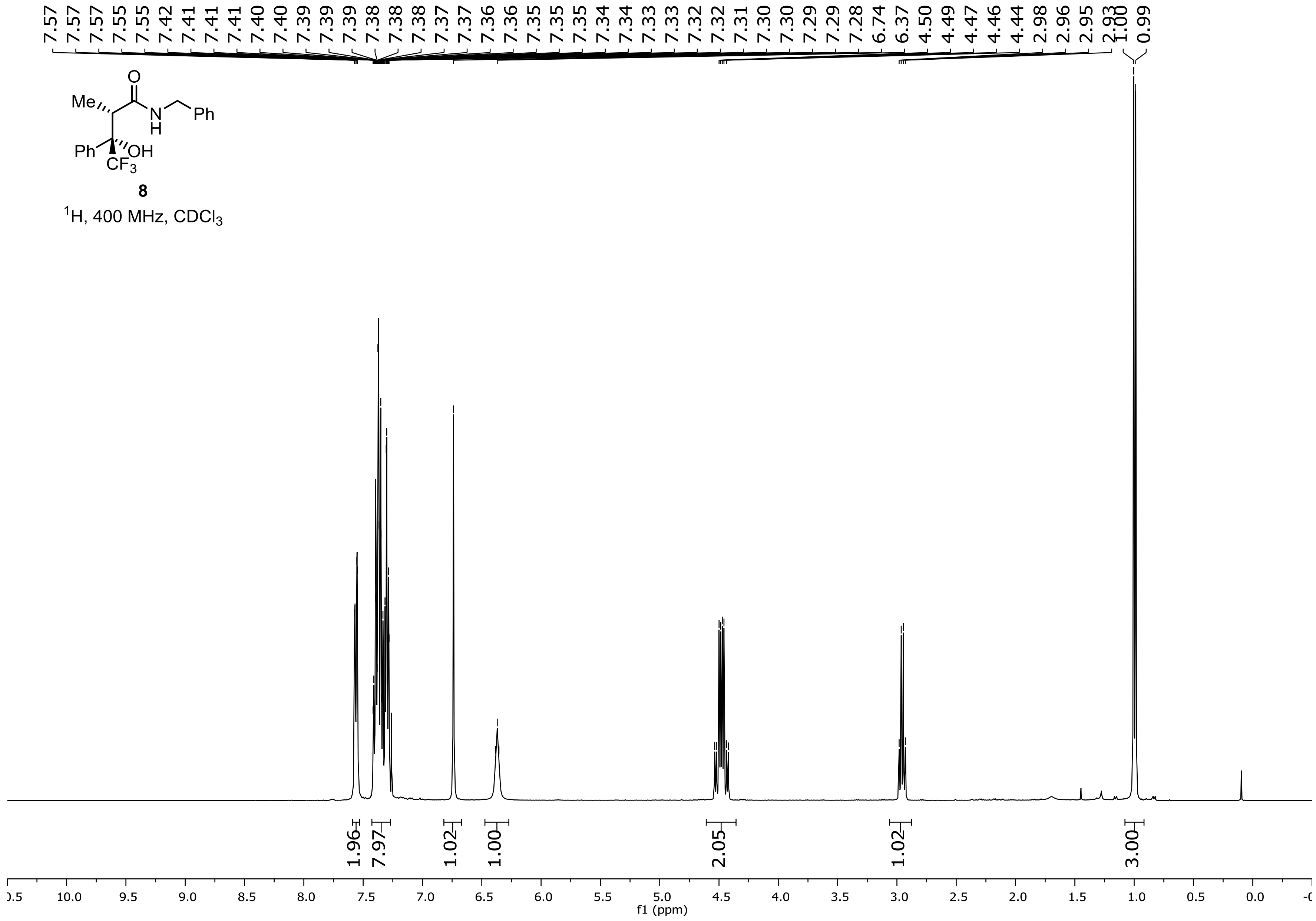
7.57 7.57 7.57 7.55 7.55 7.42 7.41 7.41 7.41 7.40 7.40 7.39 7.39 7.39 7.38 7.38 7.38 7.37 7.37 7.36 7.36 7.35 7.35 7.35 7.34 7.34 7.33 7.33 7.32 7.32 7.31 7.30 7.30 7.29 7.29 7.28 6.74 6.37 4.50 4.49 4.47 4.46 4.44 2.98 2.96 2.95 2.93 1.00 0.99
Me
O
N
H
Ph
Ph
OH
CF3
8
1H, 400 MHz, CDCl3
1.96
7.97
1.02
1.00
2.05
1.02
3.00
f1 (ppm)

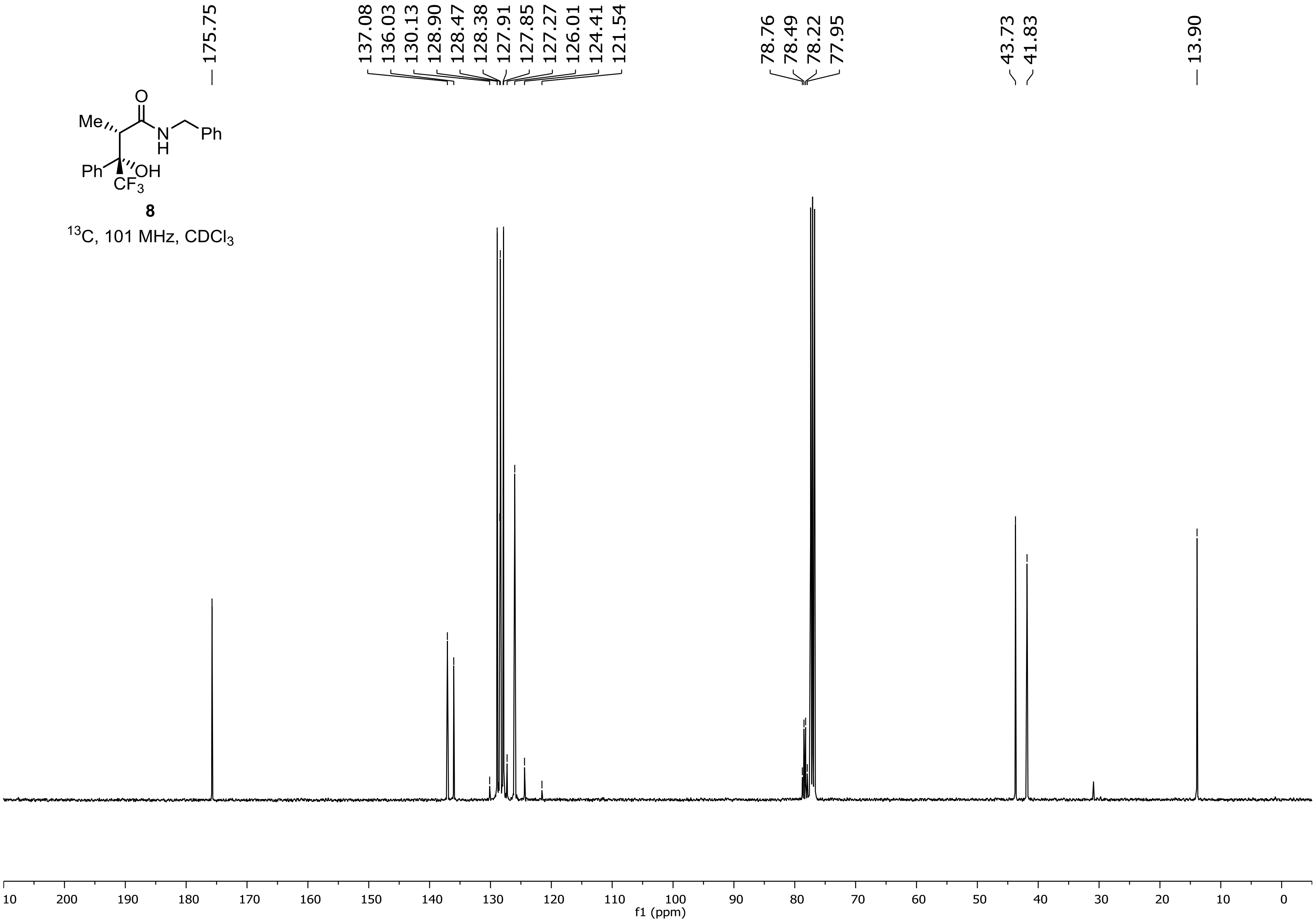
175.75
137.08
136.03
130.13
128.90
128.47
128.38
127.91
127.85
127.27
126.01
124.41
121.54
78.76
78.49
78.22
77.95
43.73
41.83
13.90
Me
O
N
H
Ph
Ph
OH
CF3
8
13C, 101 MHz, CDCl3
10 200 190 180 170 160 150 140 130 120 110 100 90 80 70 60 50 40 30 20 10 0
f1 (ppm)

8

^{19}F, 376 MHz, $CDCl_3$

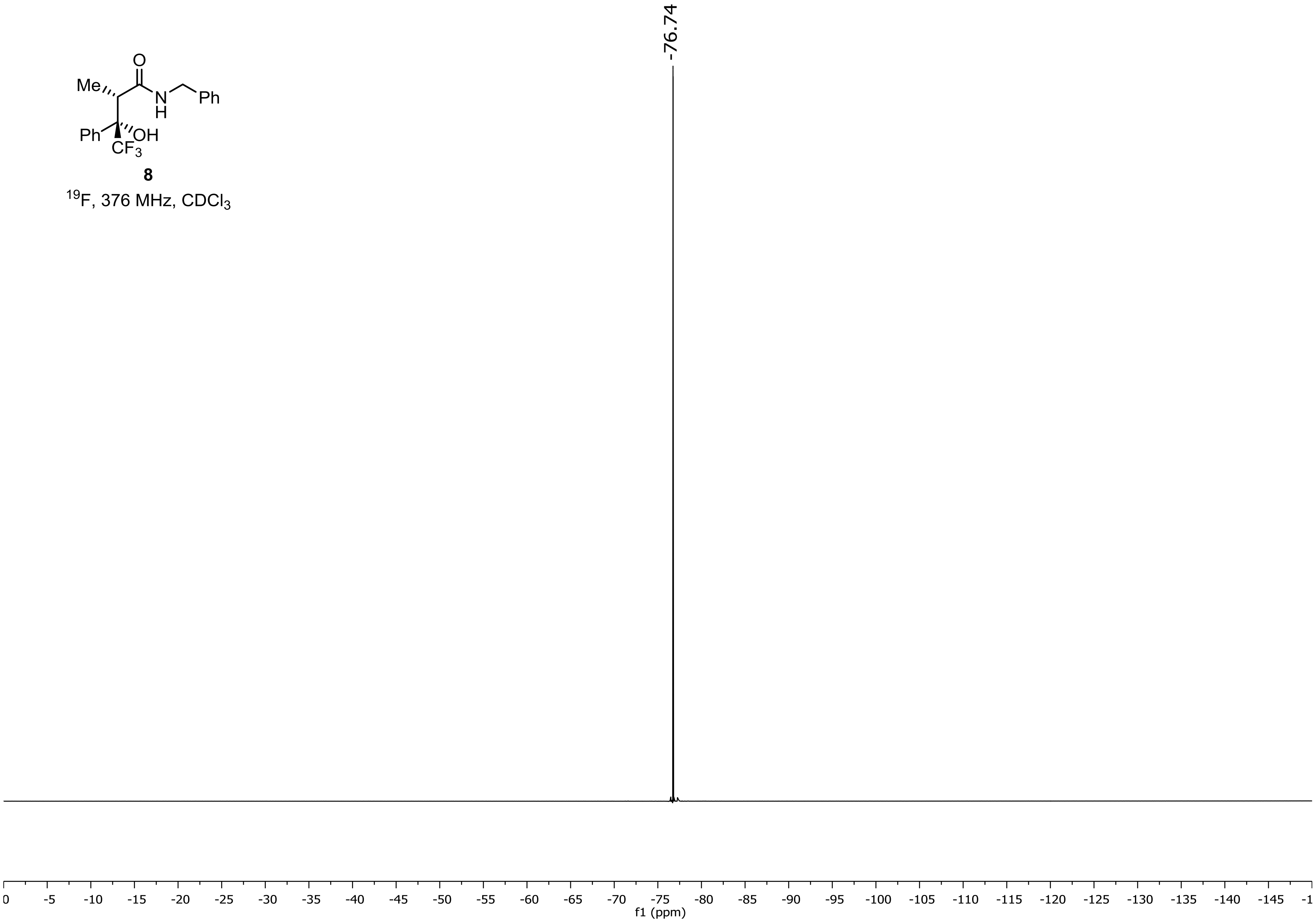

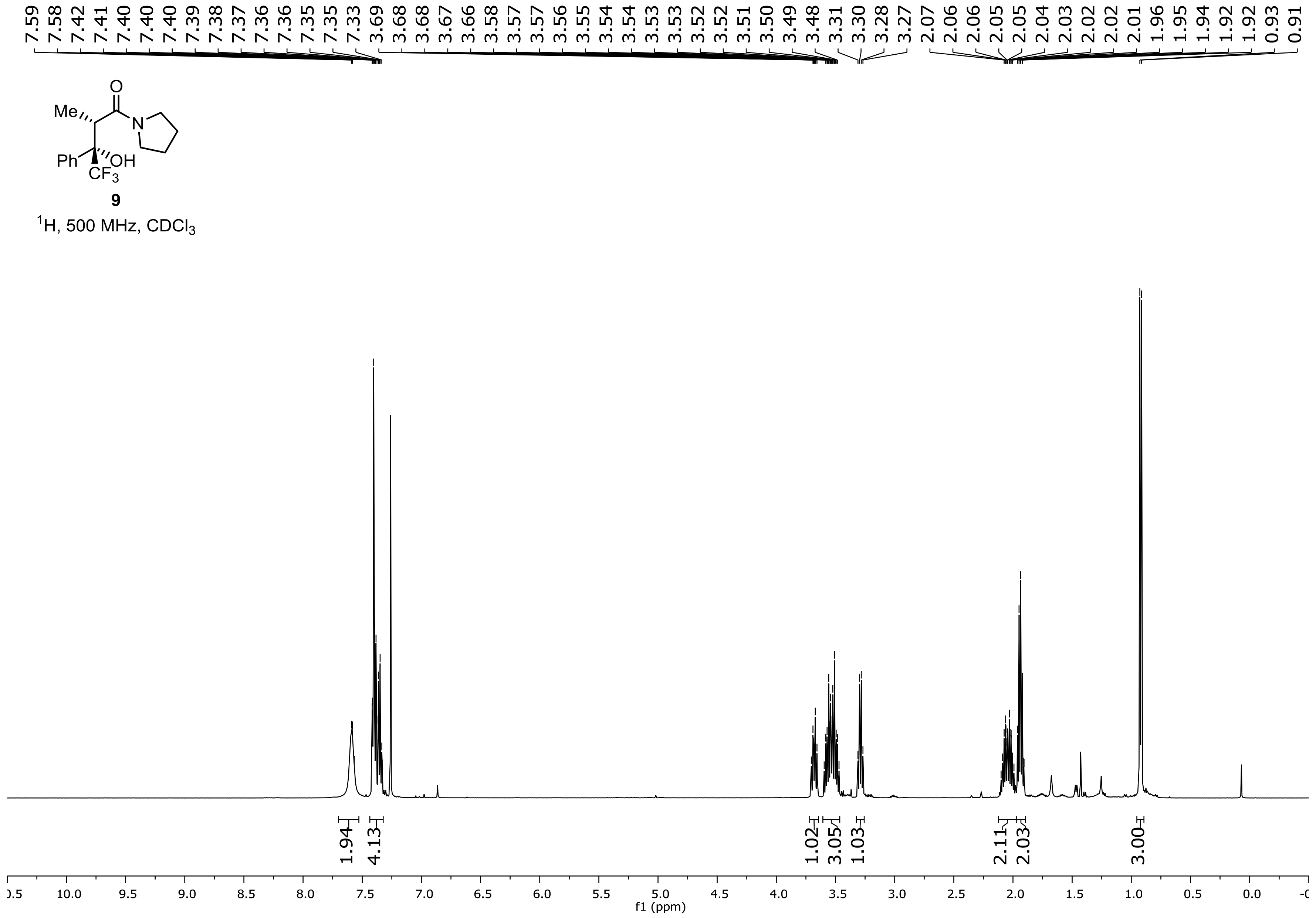
7.59 7.58 7.42 7.41 7.40 7.40 7.40 7.39 7.38 7.37 7.36 7.36 7.35 7.35 7.33 3.69 3.68 3.68 3.67 3.66 3.58 3.57 3.57 3.56 3.55 3.54 3.54 3.53 3.53 3.53 3.52 3.52 3.51 3.50 3.49 3.48 3.31 3.30 3.28 3.27 2.07 2.06 2.06 2.05 2.05 2.04 2.03 2.02 2.02 2.01 1.96 1.95 1.94 1.92 1.92 0.93 0.91
Me
O
N
Ph
OH
CF3
9
1H, 500 MHz, CDCl3
1.94
4.13
1.02
3.05
1.03
2.11
2.03
3.00
f1 (ppm)

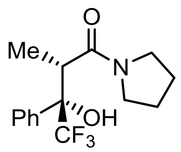

9

^{13}C, 126 MHz, $CDCl_3$

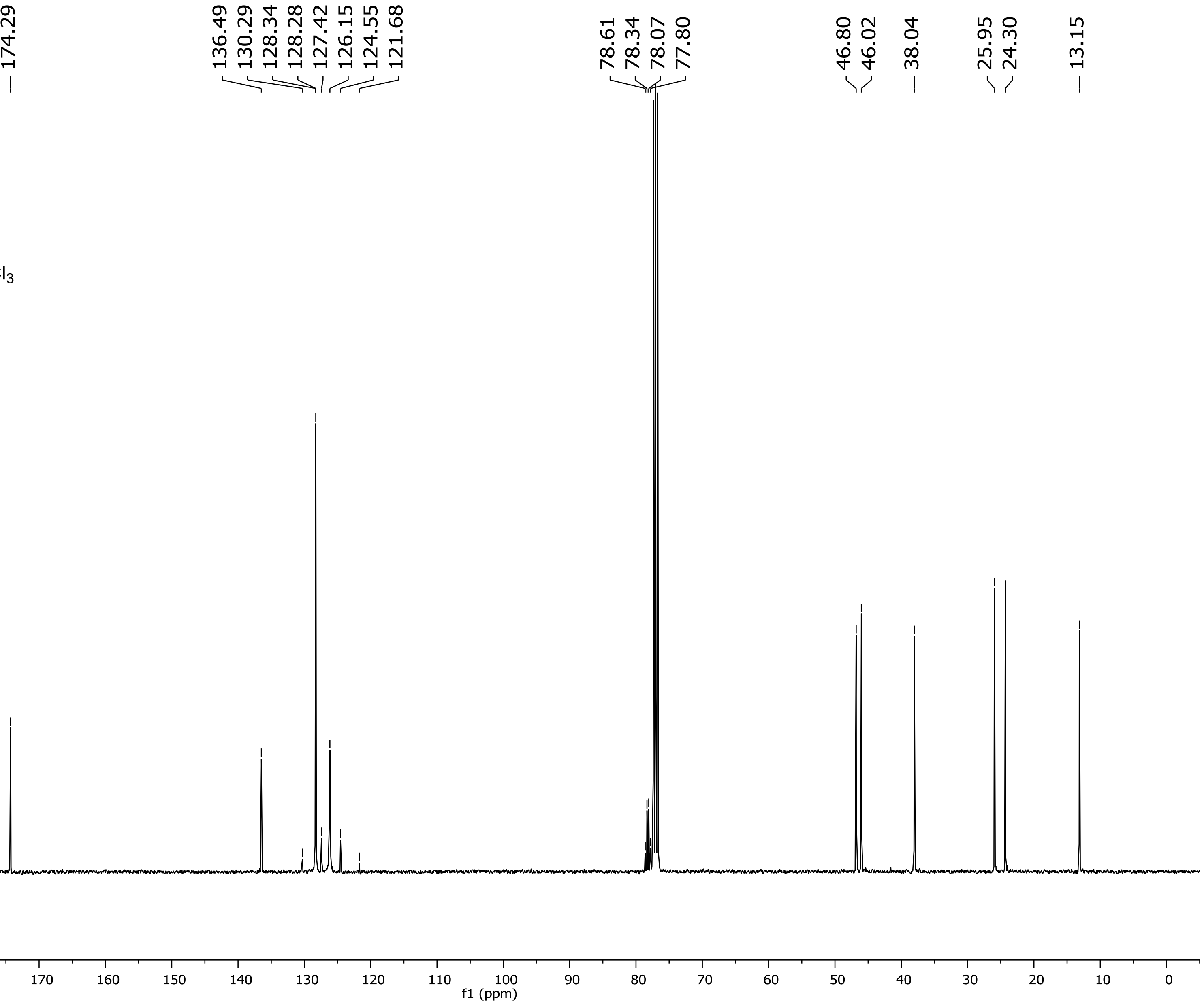

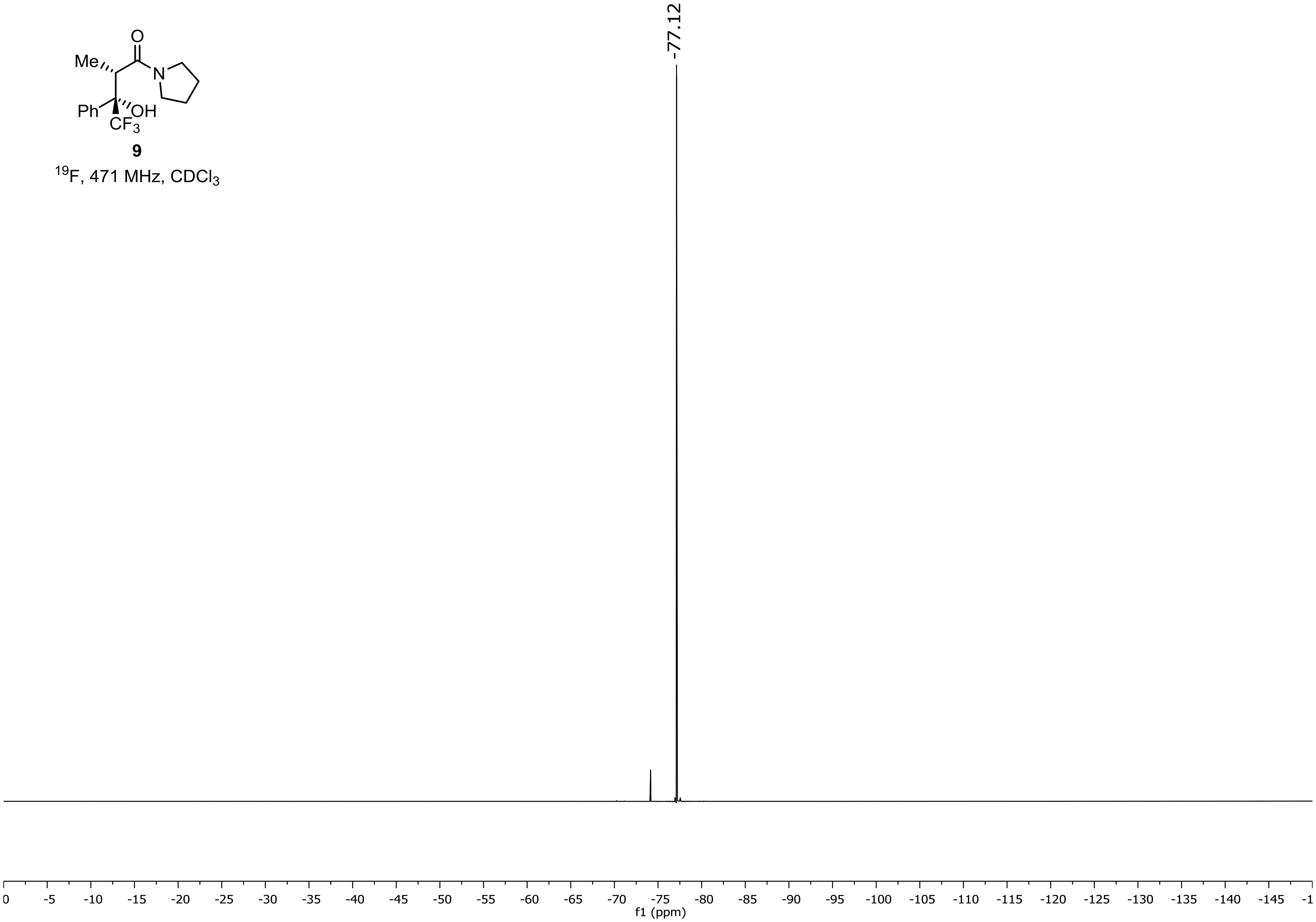
Me
O
N
Ph
OH
CF3
9
19F, 471 MHz, CDCl3
-77.12
0 -5 -10 -15 -20 -25 -30 -35 -40 -45 -50 -55 -60 -65 -70 -75 -80 -85 -90 -95 -100 -105 -110 -115 -120 -125 -130 -135 -140 -145
f1 (ppm)

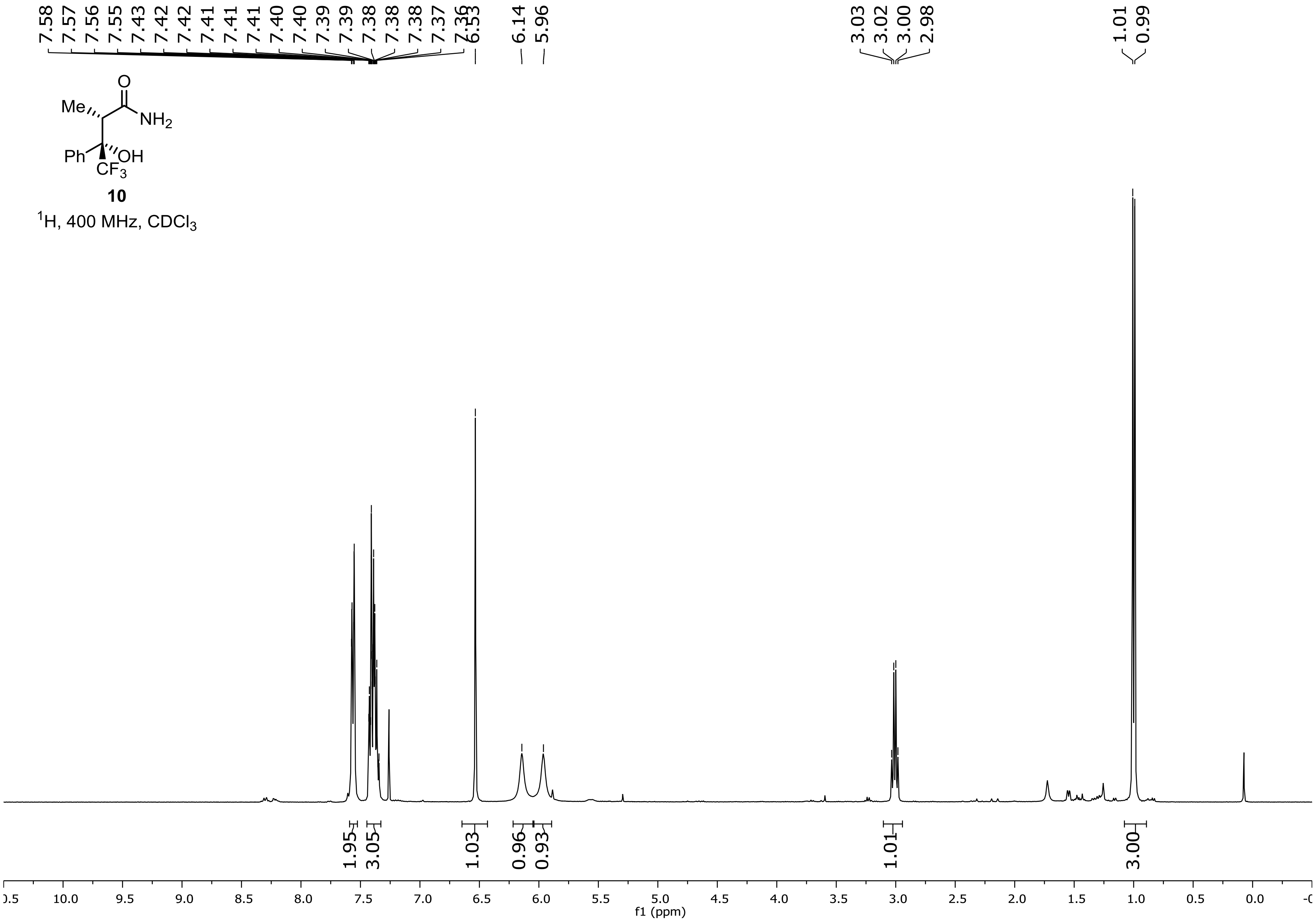
7.58 7.57 7.56 7.55 7.43 7.42 7.42 7.41 7.41 7.41 7.40 7.40 7.39 7.39 7.38 7.38 7.38 7.37 7.36 6.53
6.14
5.96
3.03 3.02 3.00 2.98
1.01 0.99
Me
O
NH2
Ph
OH
CF3
10
1H, 400 MHz, CDCl3
1.95
3.05
1.03
0.96
0.93
1.01
3.00
f1 (ppm)

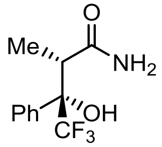

10

^{13}C, 101 MHz, $CDCl_3$

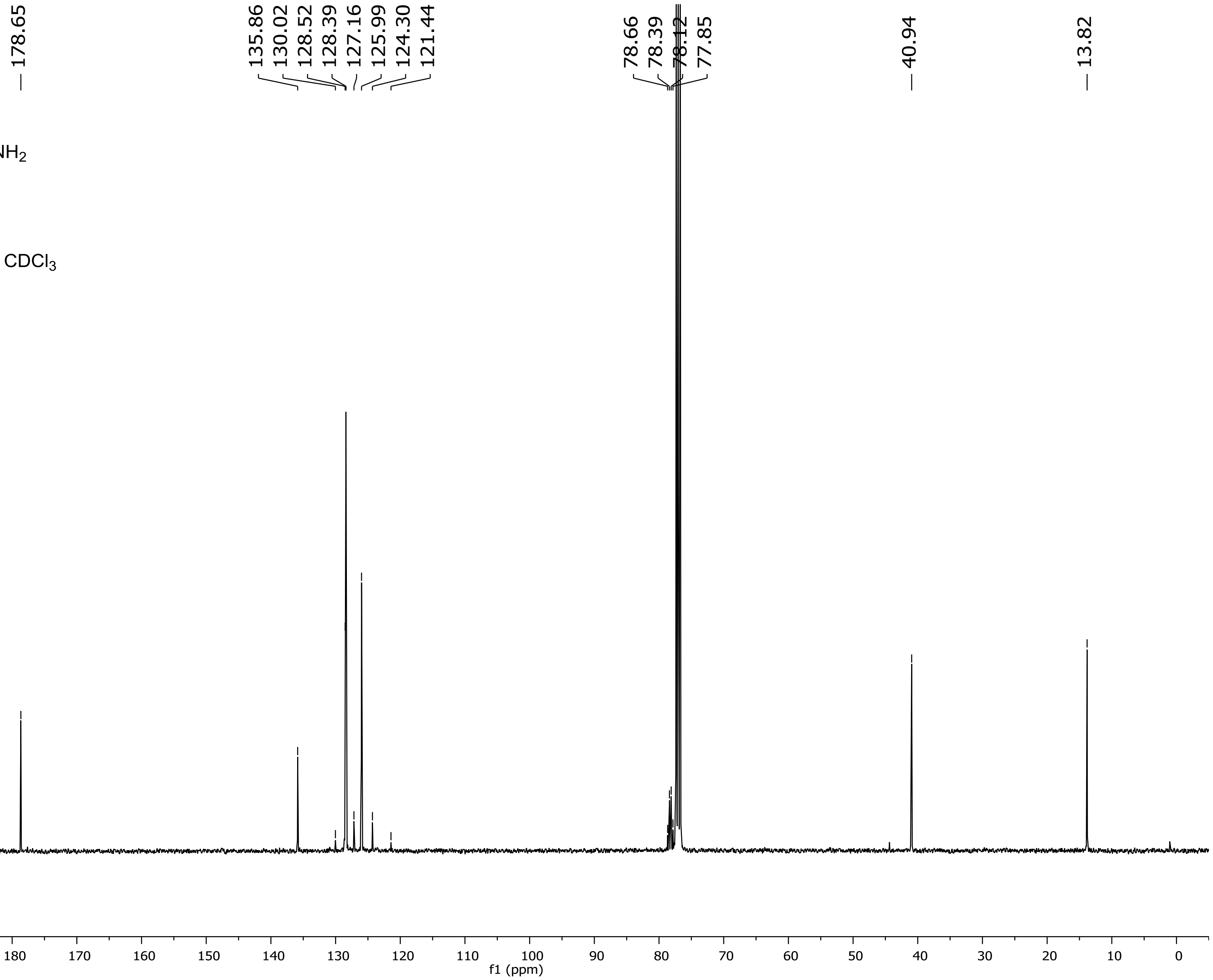

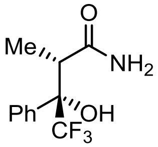

10

^{19}F, 376 MHz, $CDCl_3$

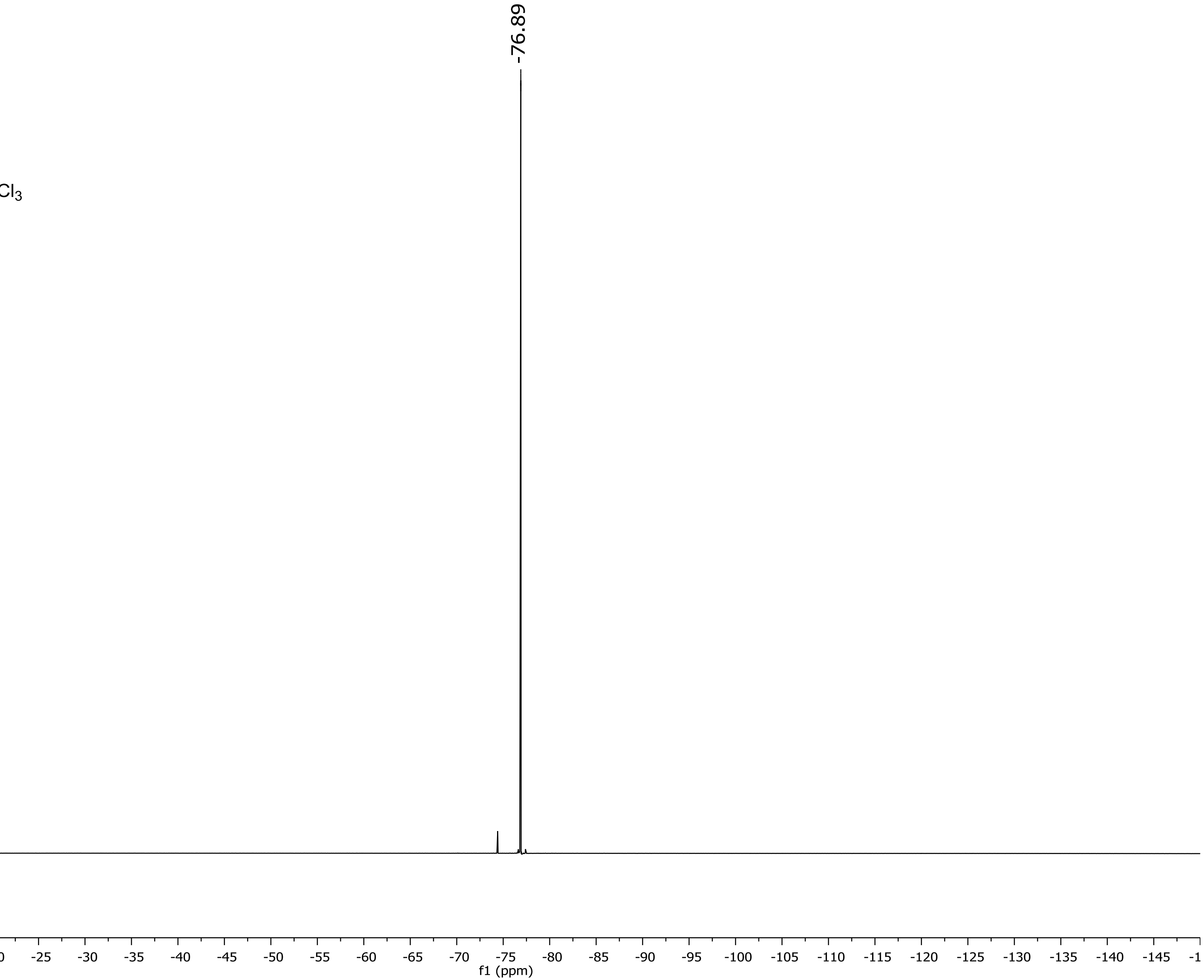

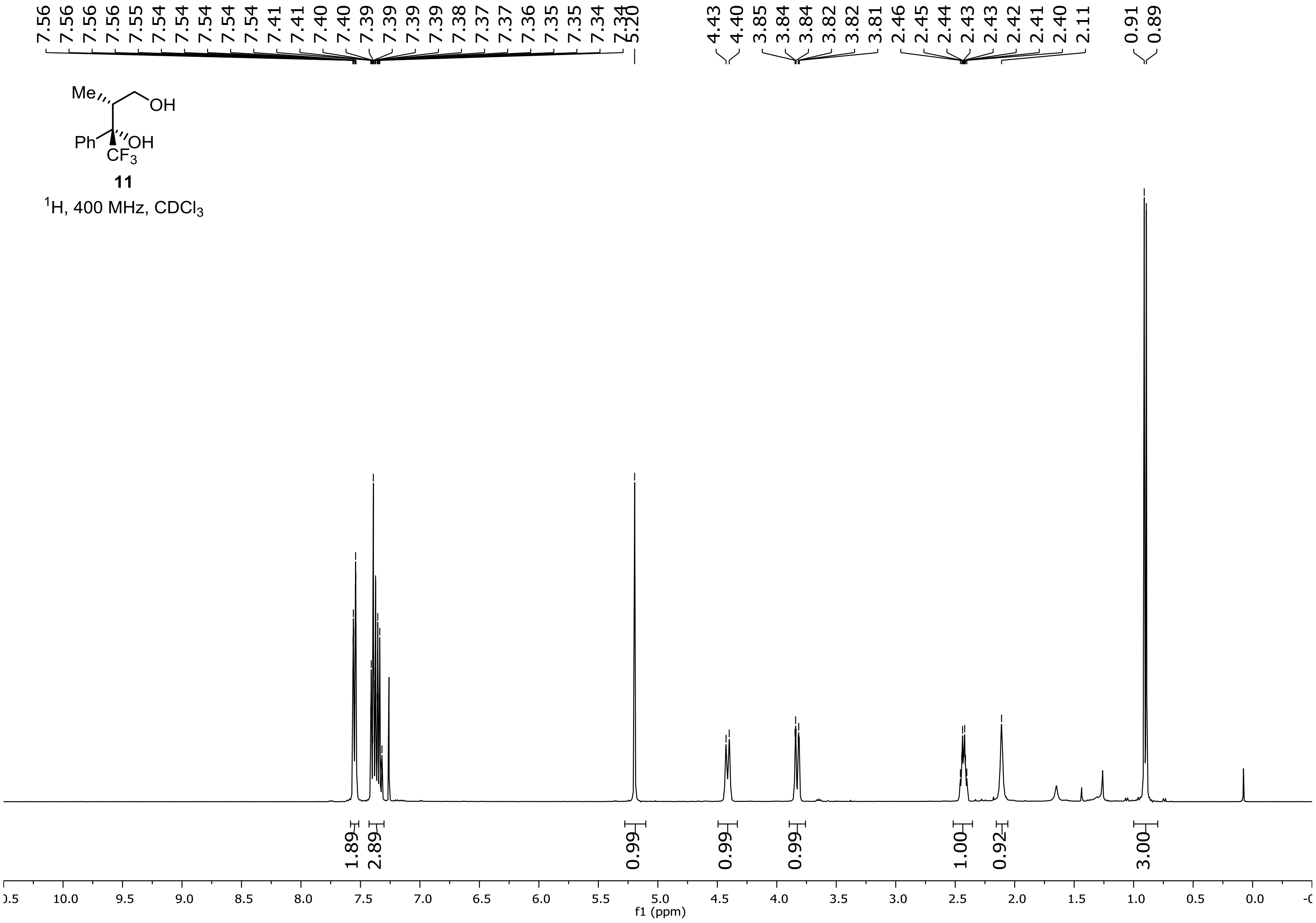
7.56 7.56 7.56 7.56 7.55 7.54 7.54 7.54 7.54 7.54 7.41 7.41 7.40 7.40 7.39 7.39 7.39 7.39 7.38 7.37 7.37 7.36 7.35 7.35 7.34 7.34 5.20
4.43 4.40 3.85 3.84 3.84 3.82 3.82 3.81 2.46 2.45 2.44 2.43 2.43 2.42 2.41 2.40 2.11
0.91 0.89
Me
OH
Ph
OH
CF3
11
1H, 400 MHz, CDCl3
1.89
2.89
0.99
0.99
0.99
1.00
0.92
3.00
10.0 9.5 9.0 8.5 8.0 7.5 7.0 6.5 6.0 5.5 5.0 4.5 4.0 3.5 3.0 2.5 2.0 1.5 1.0 0.5 0.0
f1 (ppm)

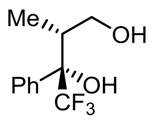

11

^{13}C, 101 MHz, $CDCl_3$

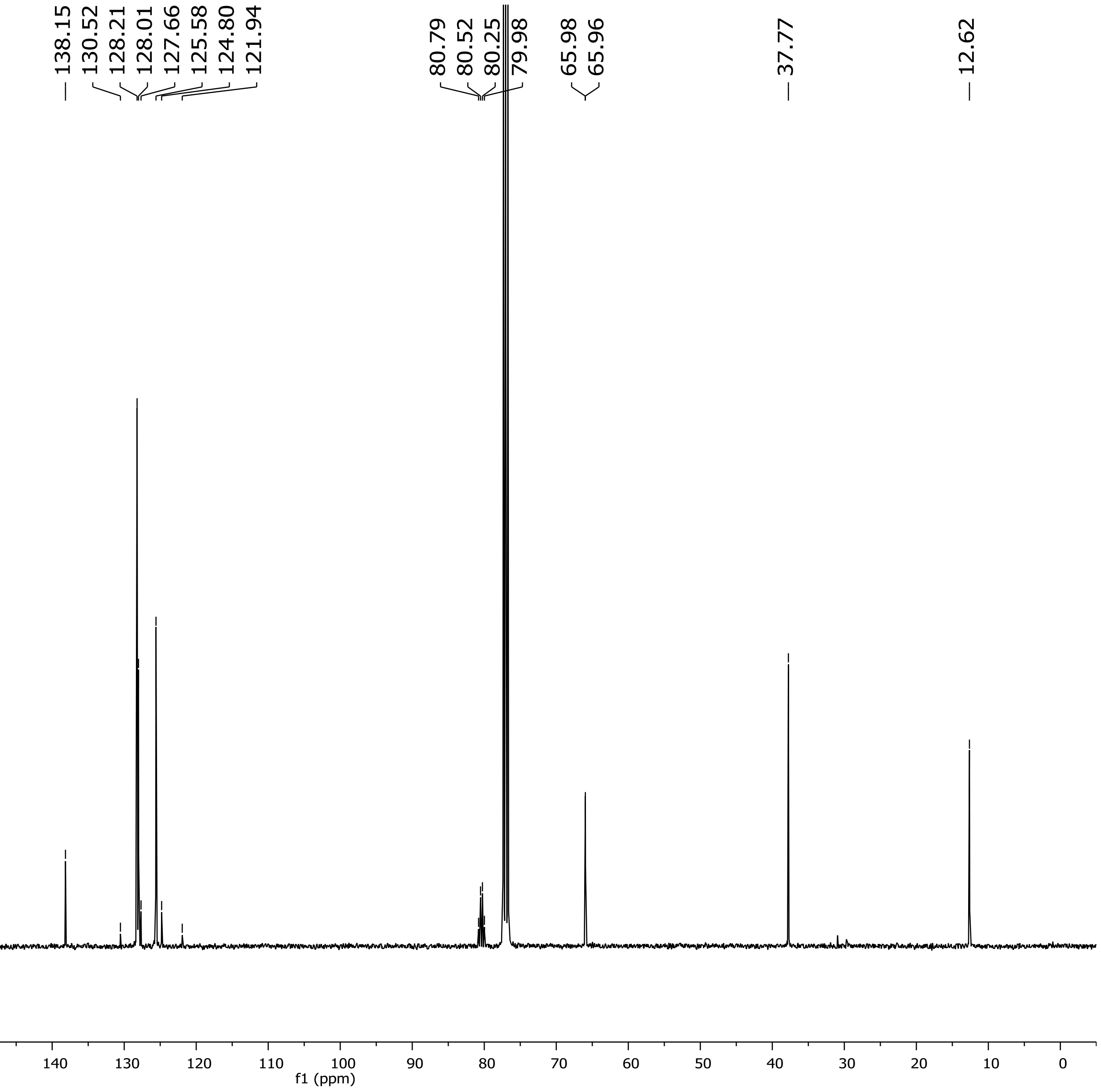

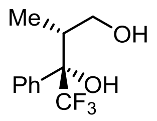

11

^{19}F, 376 MHz, $CDCl_3$

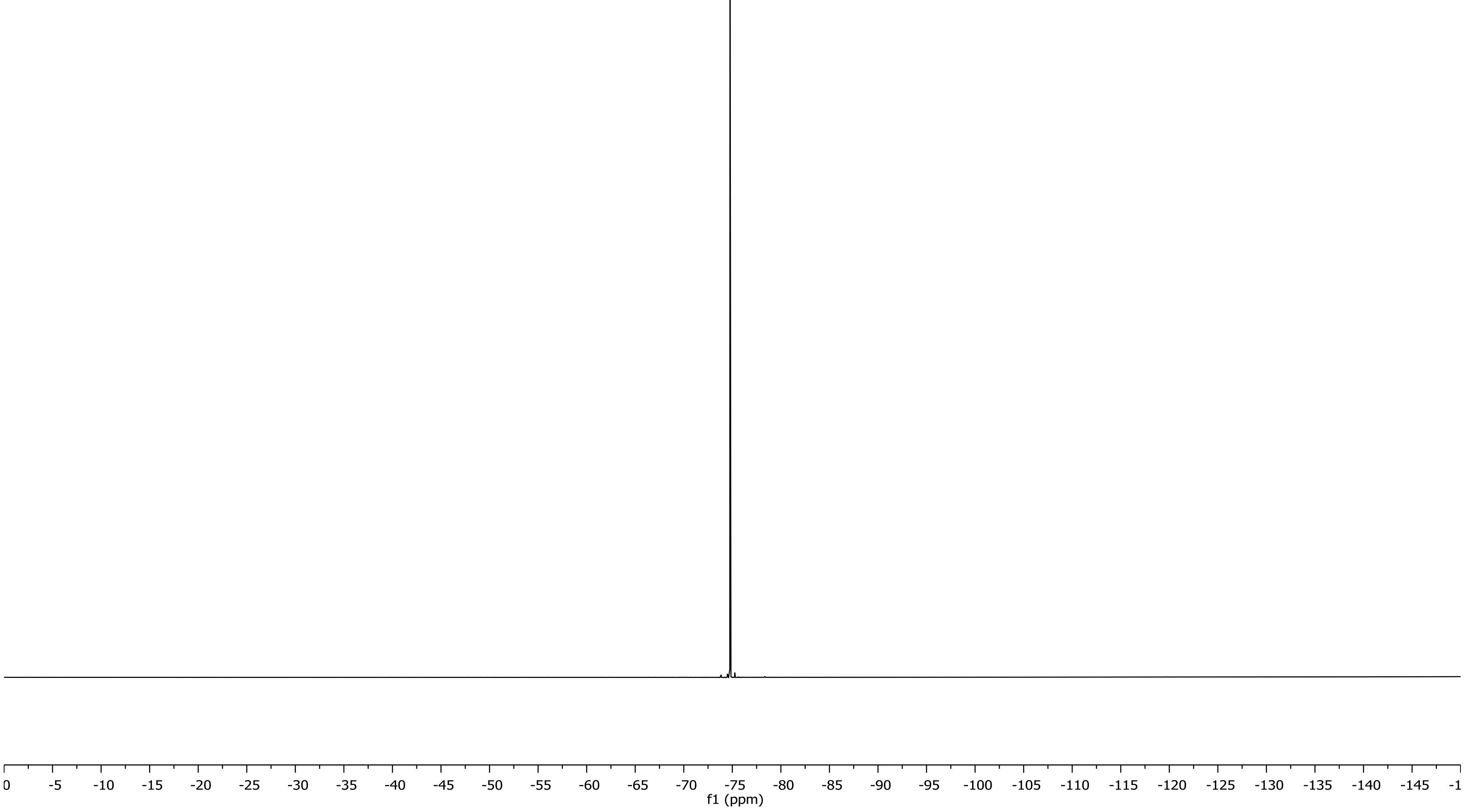

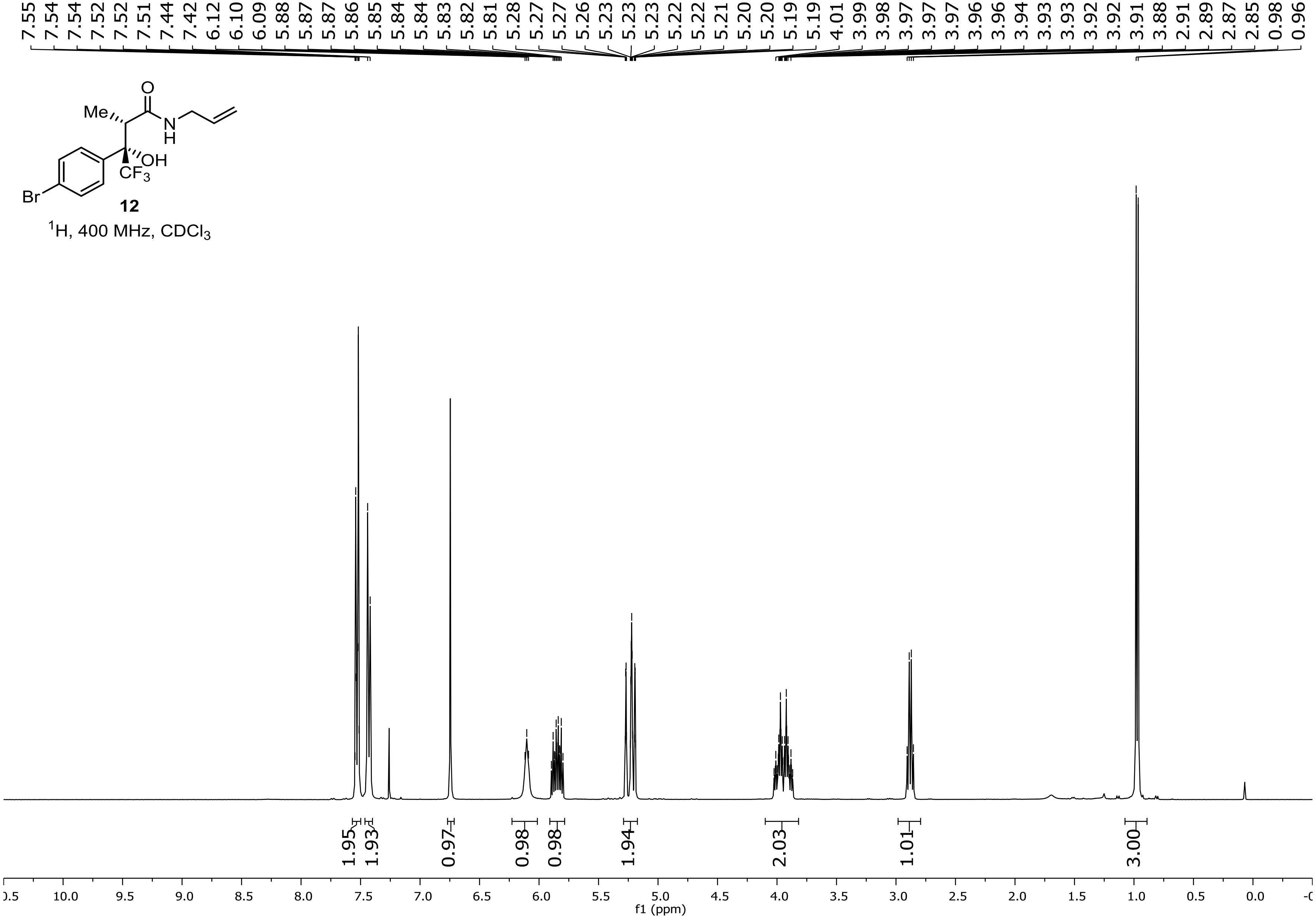

^{1}H, 400 MHz, $CDCl_3$

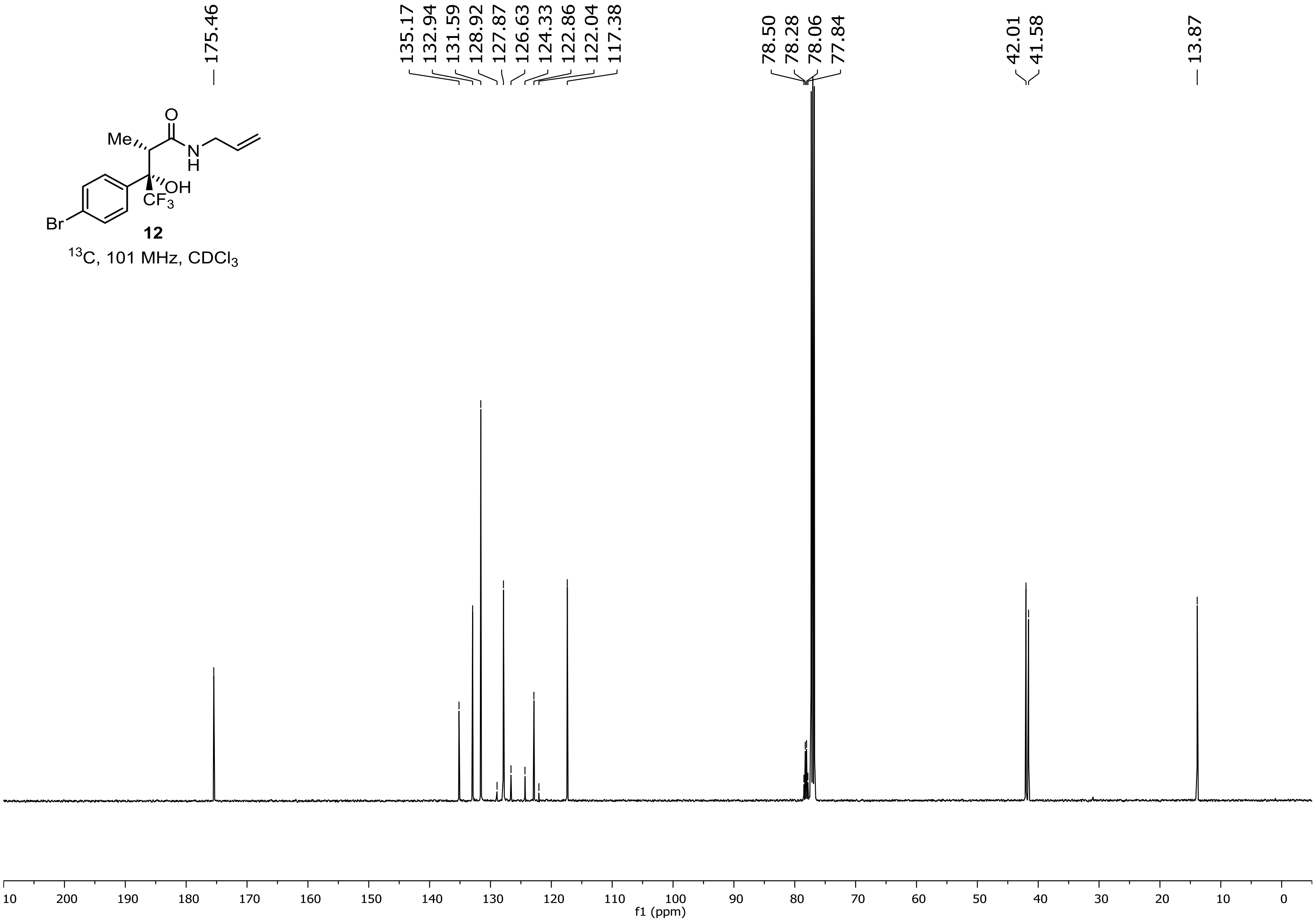
175.46
135.17
132.94
131.59
128.92
127.87
126.63
124.33
122.86
122.04
117.38
78.50
78.28
78.06
77.84
42.01
41.58
13.87
Me
O
N
H
OH
CF3
Br
12
13C, 101 MHz, CDCl3
f1 (ppm)

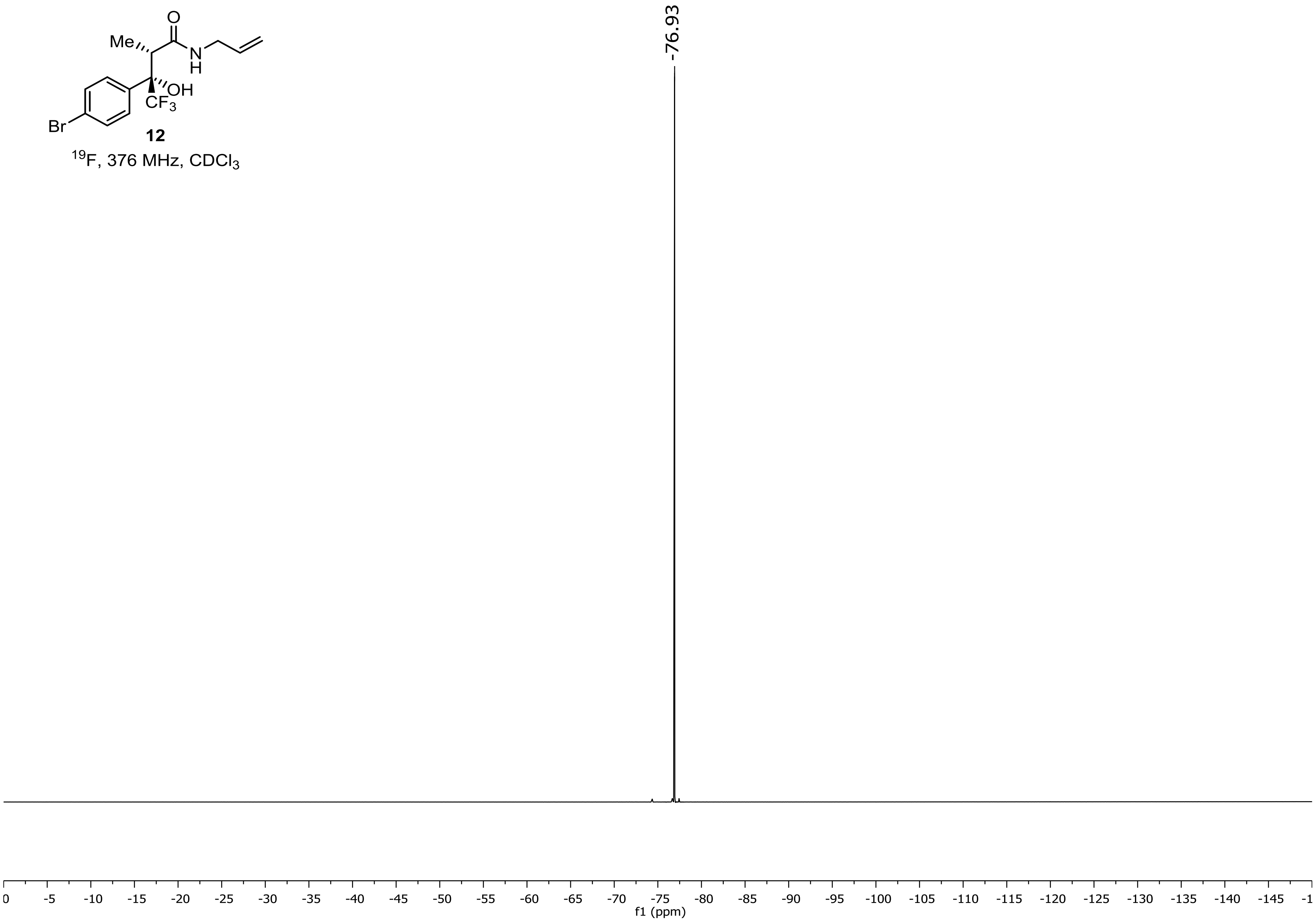

Me
O
N
H
OH
CF3
Br
12
19F, 376 MHz, CDCl3
-76.93
0 -5 -10 -15 -20 -25 -30 -35 -40 -45 -50 -55 -60 -65 -70 -75 -80 -85 -90 -95 -100 -105 -110 -115 -120 -125 -130 -135 -140 -145
f1 (ppm)

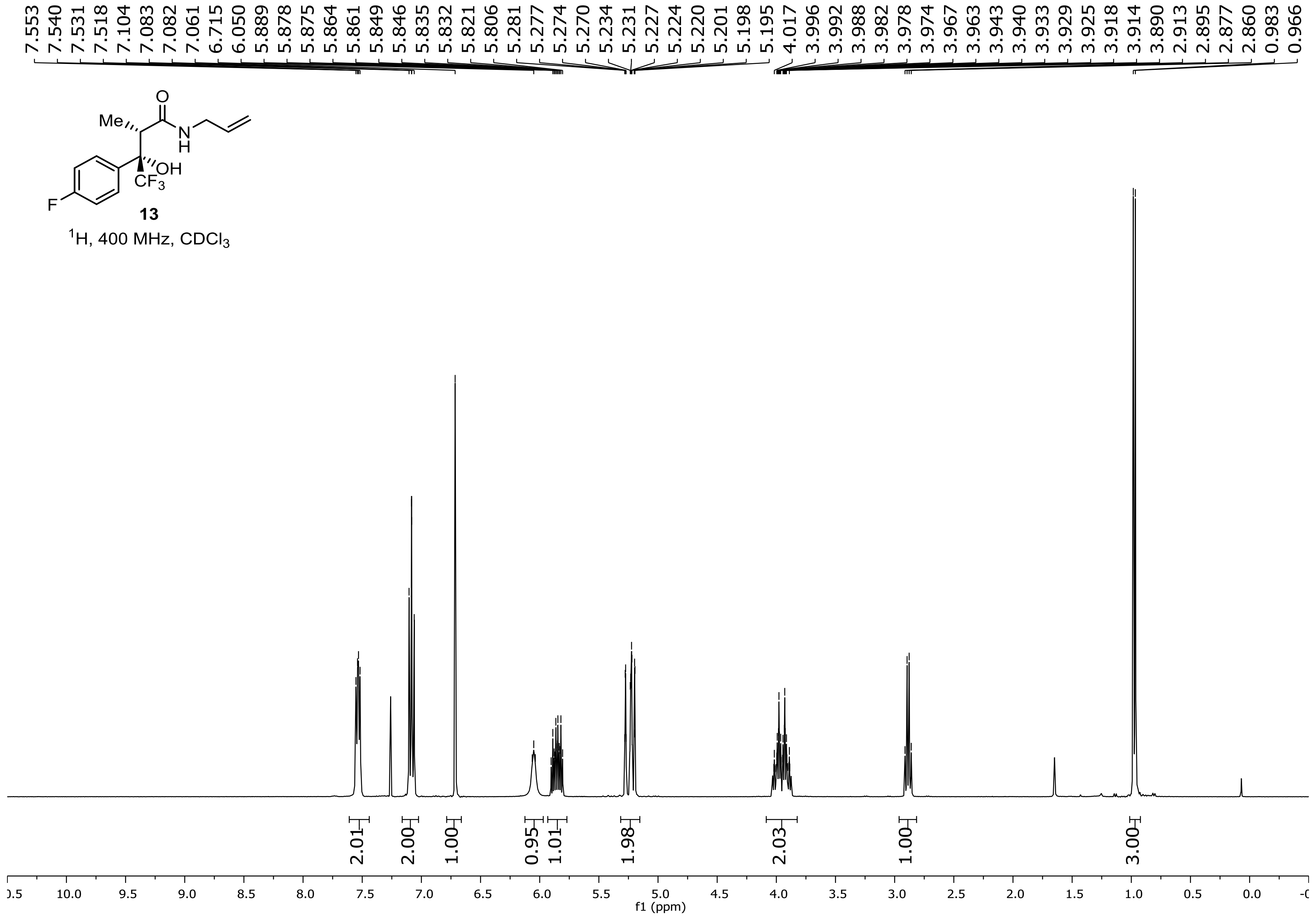
7.553 7.540 7.531 7.518 7.104 7.083 7.082 7.061 6.715 6.050 5.889 5.878 5.875 5.864 5.861 5.849 5.846 5.835 5.832 5.821 5.806 5.281 5.277 5.274 5.270 5.234 5.231 5.227 5.224 5.220 5.201 5.198 5.195 4.017 3.996 3.992 3.988 3.982 3.978 3.974 3.967 3.963 3.943 3.940 3.933 3.929 3.925 3.918 3.914 3.890 2.913 2.895 2.877 2.860 0.983 0.966
Me
O
N
H
OH
CF3
F
13
1H, 400 MHz, CDCl3
2.01
2.00
1.00
0.95
1.01
1.98
2.03
1.00
3.00
f1 (ppm)

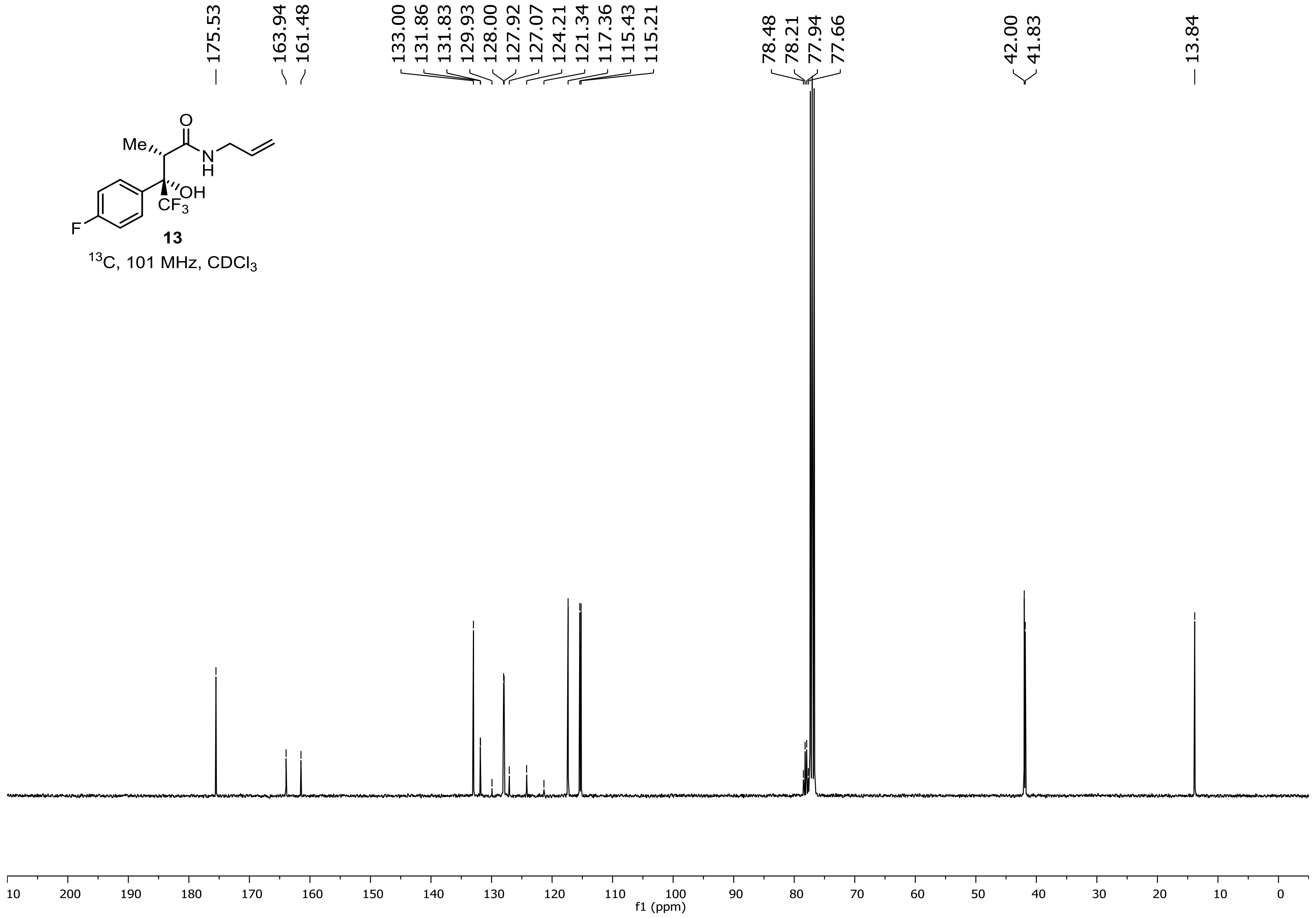

175.53
163.94
161.48
133.00
131.86
131.83
129.93
128.00
127.92
127.07
124.21
121.34
117.36
115.43
115.21
78.48
78.21
77.94
77.66
42.00
41.83
13.84
Me
O
N
H
OH
CF3
F
13
13C, 101 MHz, CDCl3
10
200
190
180
170
160
150
140
130
120
110
100
90
80
70
60
50
40
30
20
10
0
f1 (ppm)

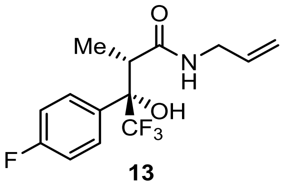

13

^{19}F, 376 MHz, $CDCl_3$

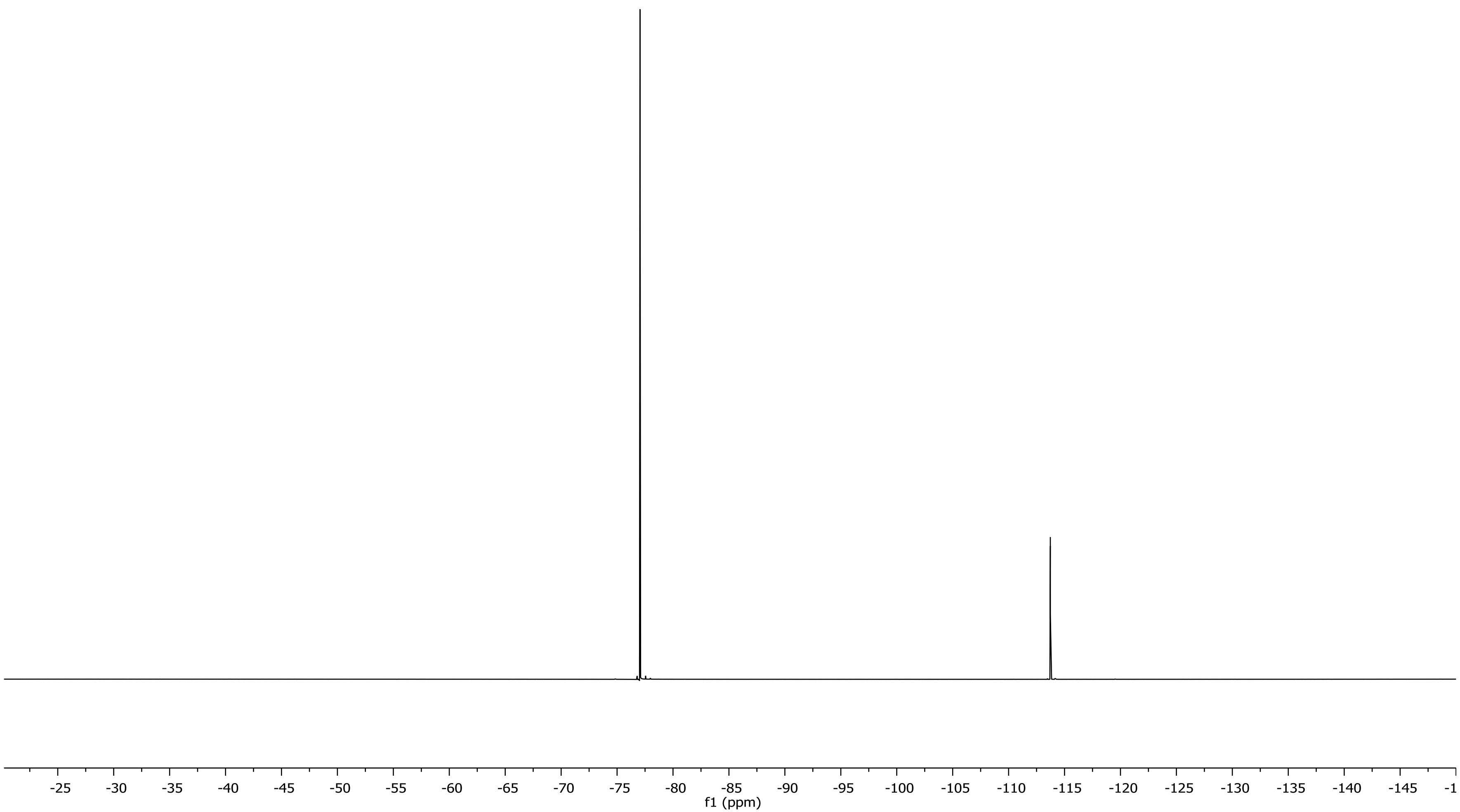

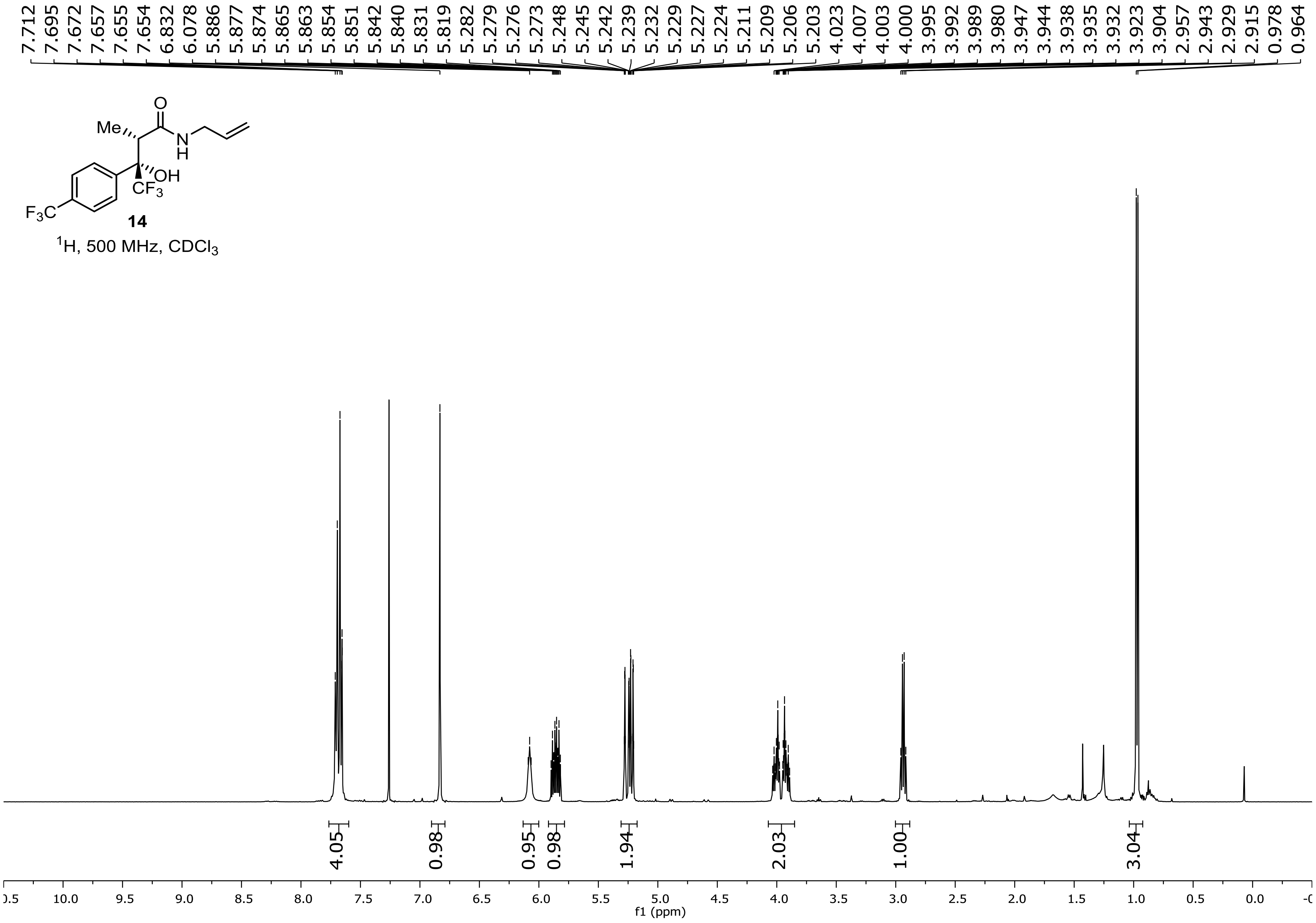
Me
O
N
H
OH
CF3
F3C
14
1H, 500 MHz, CDCl3
7.712
7.695
7.672
7.657
7.655
7.654
6.832
6.078
5.886
5.877
5.874
5.865
5.863
5.854
5.851
5.842
5.840
5.831
5.819
5.282
5.279
5.276
5.273
5.248
5.245
5.242
5.239
5.232
5.229
5.227
5.224
5.211
5.209
5.206
5.203
4.023
4.007
4.003
4.000
3.995
3.992
3.989
3.980
3.947
3.944
3.938
3.935
3.932
3.923
3.904
2.957
2.943
2.929
2.915
0.978
0.964
4.05
0.98
0.95
0.98
1.94
2.03
1.00
3.04
10.0
9.5
9.0
8.5
8.0
7.5
7.0
6.5
6.0
5.5
5.0
4.5
4.0
3.5
3.0
2.5
2.0
1.5
1.0
0.5
0.0
f1 (ppm)

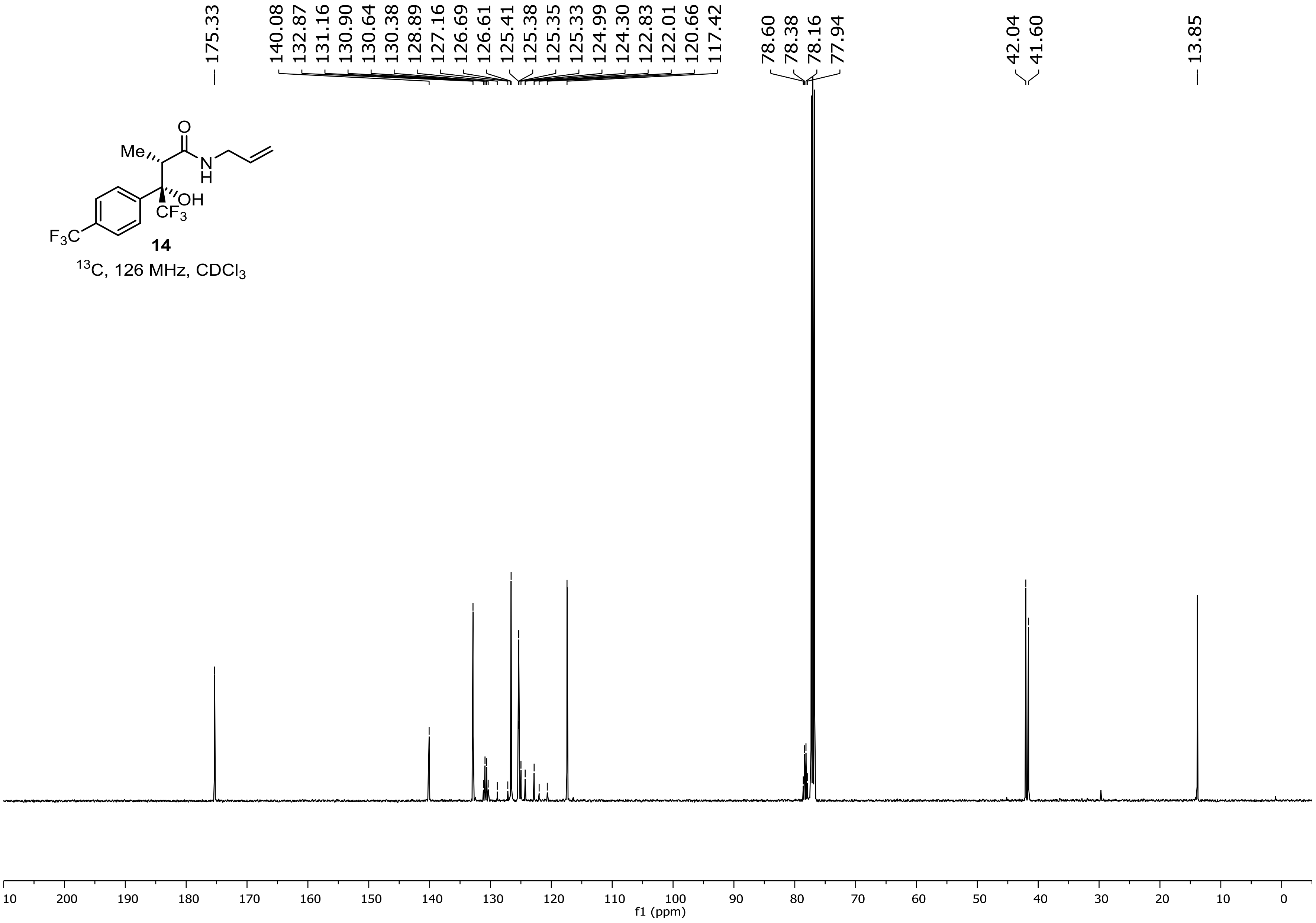
175.33
140.08
132.87
131.16
130.90
130.64
130.38
128.89
127.16
126.69
126.61
125.41
125.38
125.35
125.33
124.99
124.30
122.83
122.01
120.66
117.42
78.60
78.38
78.16
77.94
42.04
41.60
13.85
Me
O
N
H
OH
CF3
F3C
14
13C, 126 MHz, CDCl3
10
200
190
180
170
160
150
140
130
120
110
100
90
80
70
60
50
40
30
20
10
0
f1 (ppm)

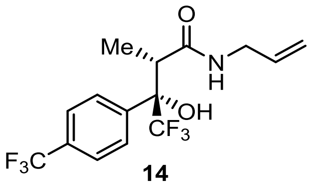

14

^{19}F, 471 MHz, $CDCl_3$

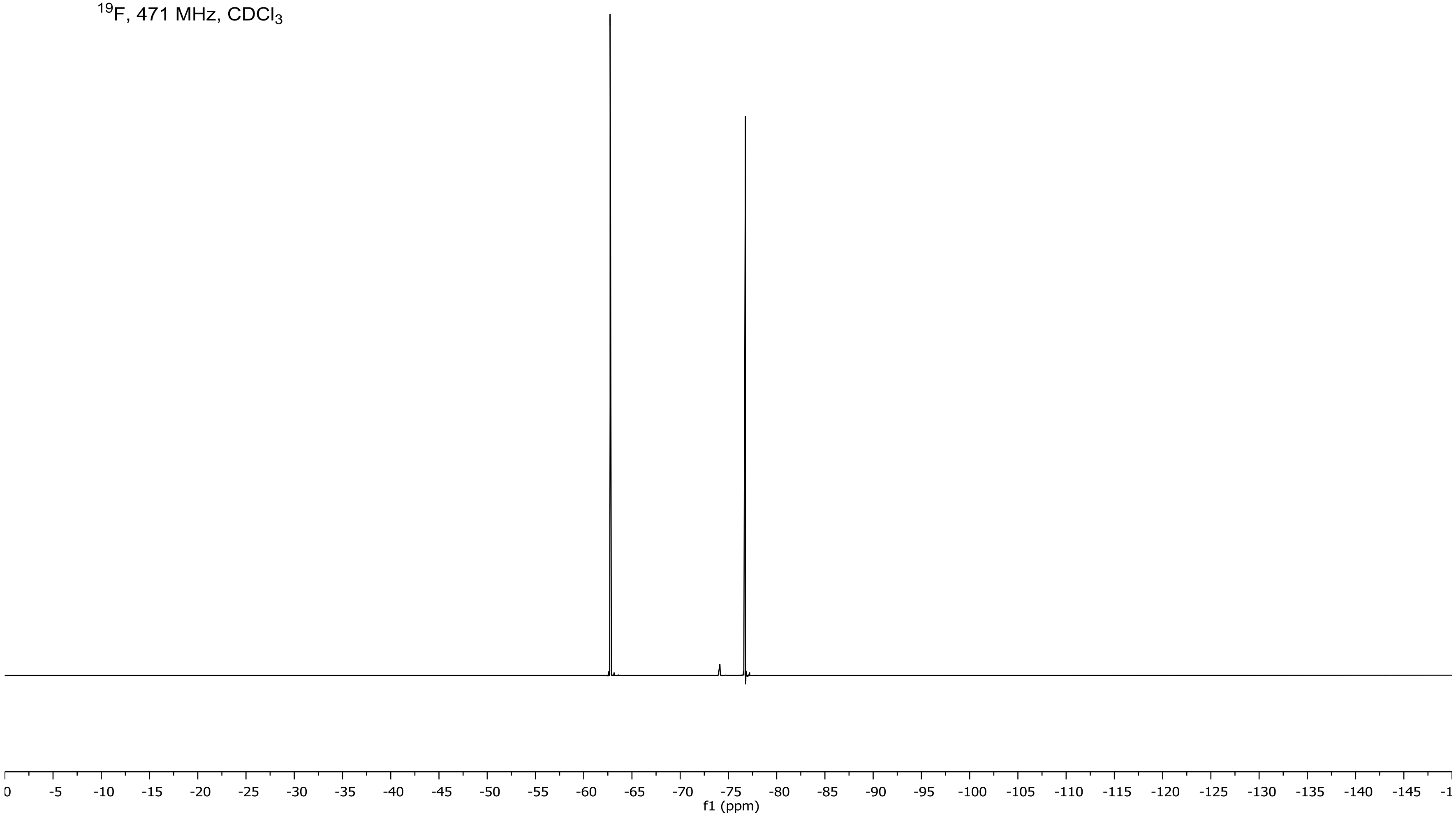

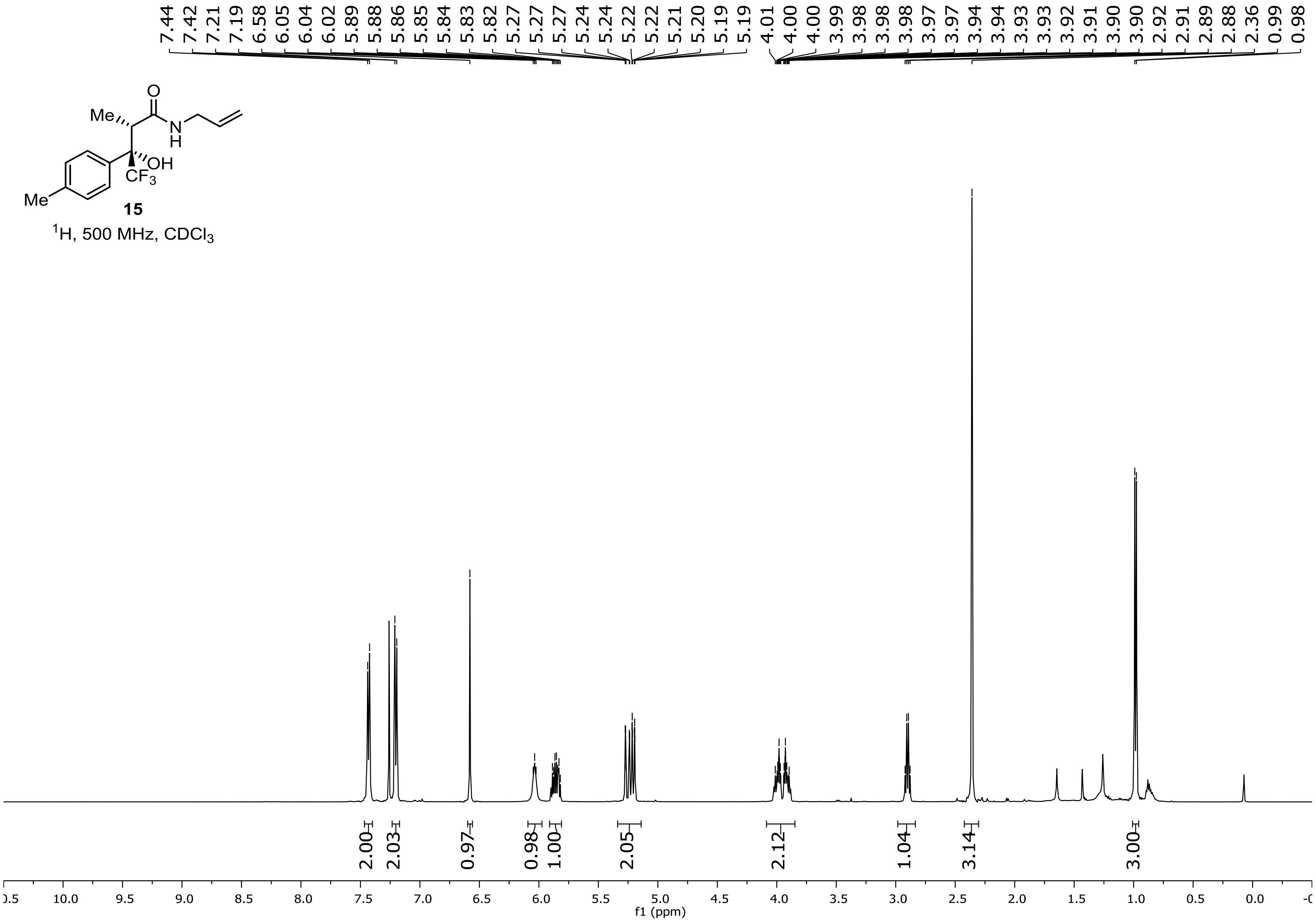
7.44 7.42 7.21 7.19 6.58 6.05 6.04 6.02 5.89 5.88 5.86 5.85 5.84 5.83 5.82 5.27 5.27 5.27 5.24 5.24 5.22 5.22 5.21 5.20 5.19 5.19 4.01 4.00 4.00 3.99 3.98 3.98 3.98 3.97 3.97 3.94 3.94 3.93 3.93 3.92 3.91 3.90 3.90 2.92 2.91 2.89 2.88 2.36 0.99 0.98
Me
O
N
H
OH
CF3
Me
15
1H, 500 MHz, CDCl3
2.00
2.03
0.97
0.98
1.00
2.05
2.12
1.04
3.14
3.00
10.0 9.5 9.0 8.5 8.0 7.5 7.0 6.5 6.0 5.5 5.0 4.5 4.0 3.5 3.0 2.5 2.0 1.5 1.0 0.5 0.0
f1 (ppm)

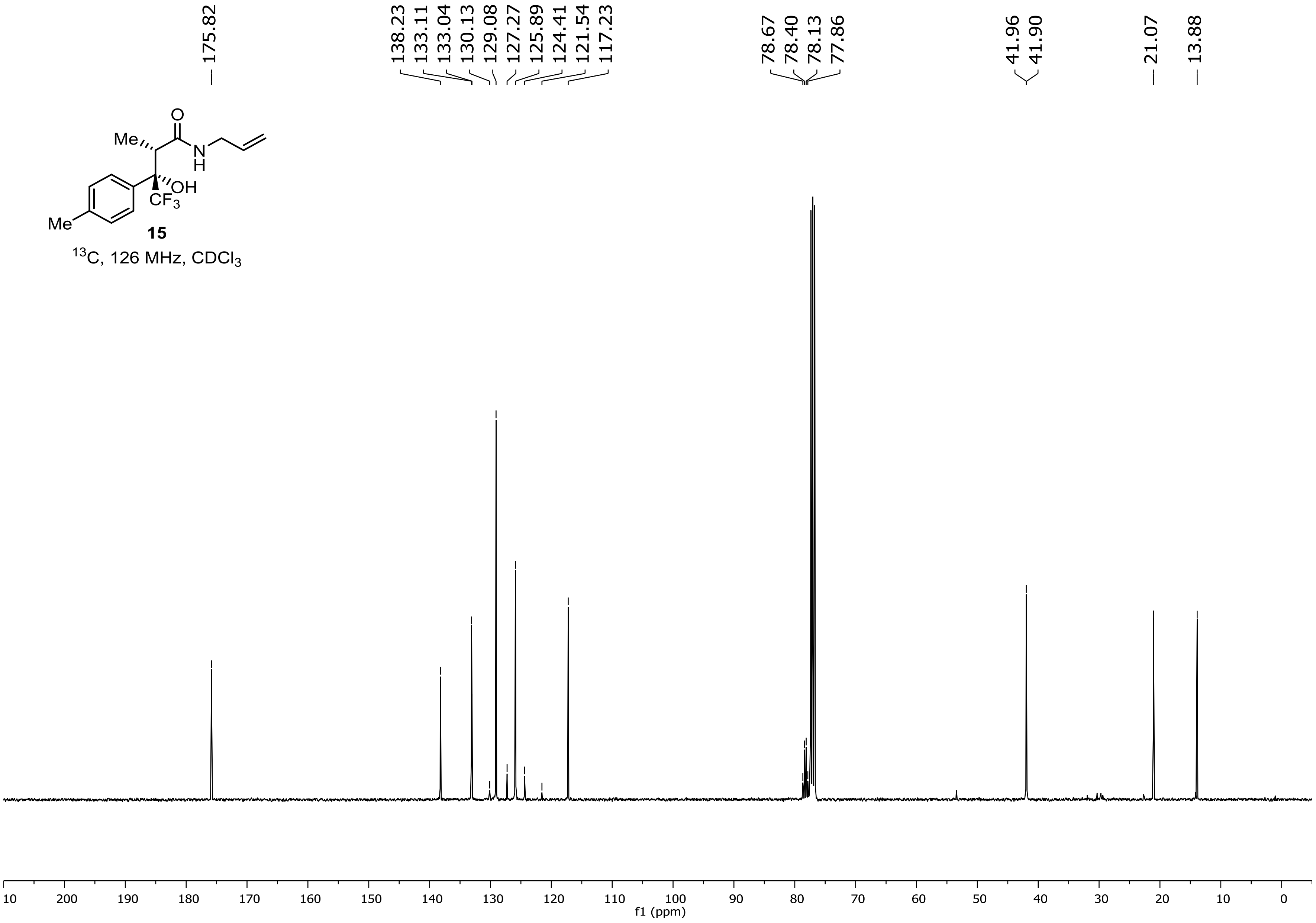
175.82
138.23
133.11
133.04
130.13
129.08
127.27
125.89
124.41
121.54
117.23
78.67
78.40
78.13
77.86
41.96
41.90
21.07
13.88
O
Me
N
H
OH
CF_3
Me
15
^{13}C, 126 MHz, $CDCl_3$
10
200
190
180
170
160
150
140
130
120
110
100
90
80
70
60
50
40
30
20
10
0
f1 (ppm)

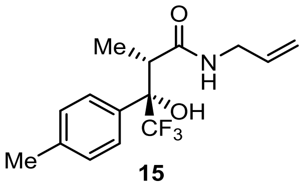

15

^{19}F, 471 MHz, $CDCl_3$

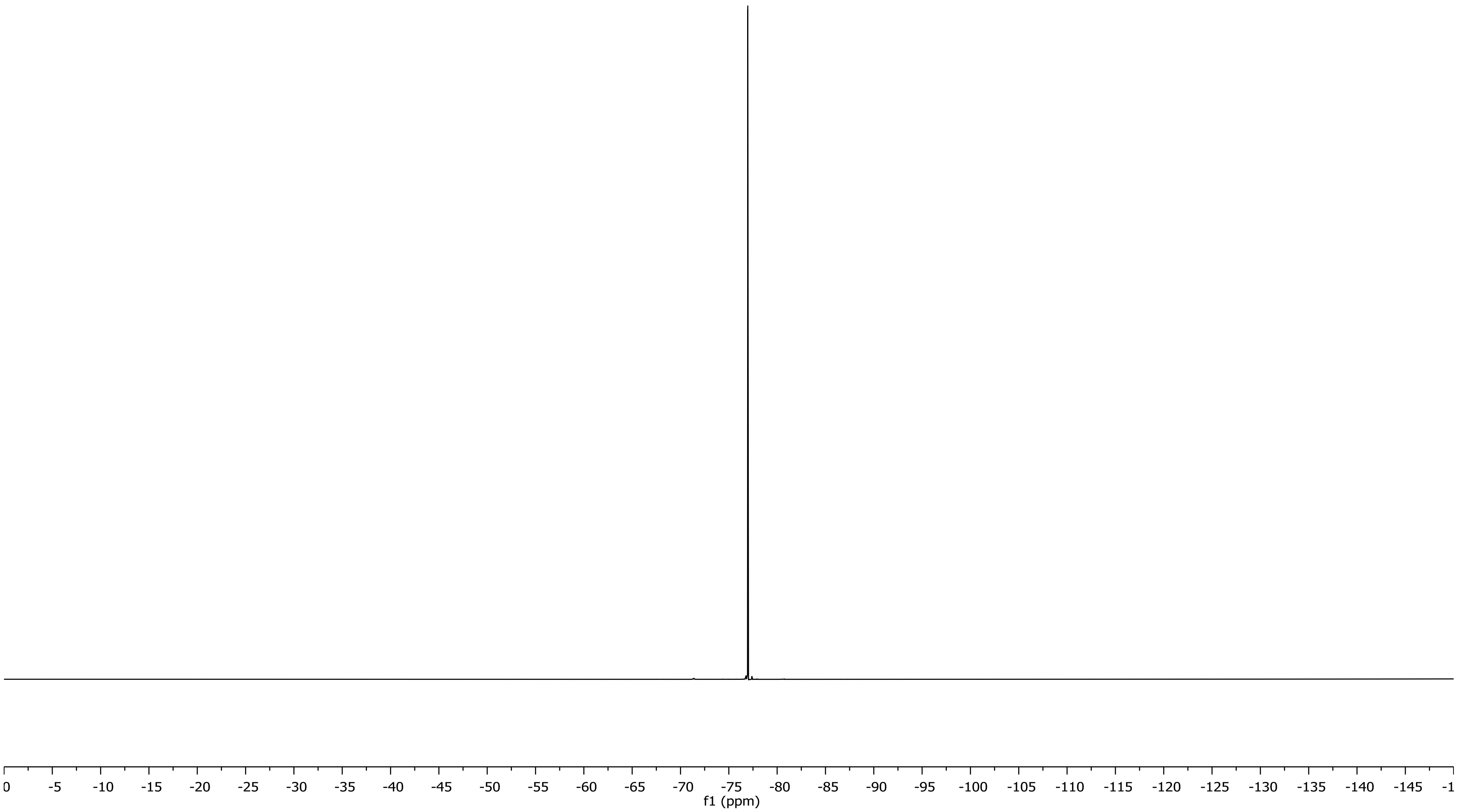

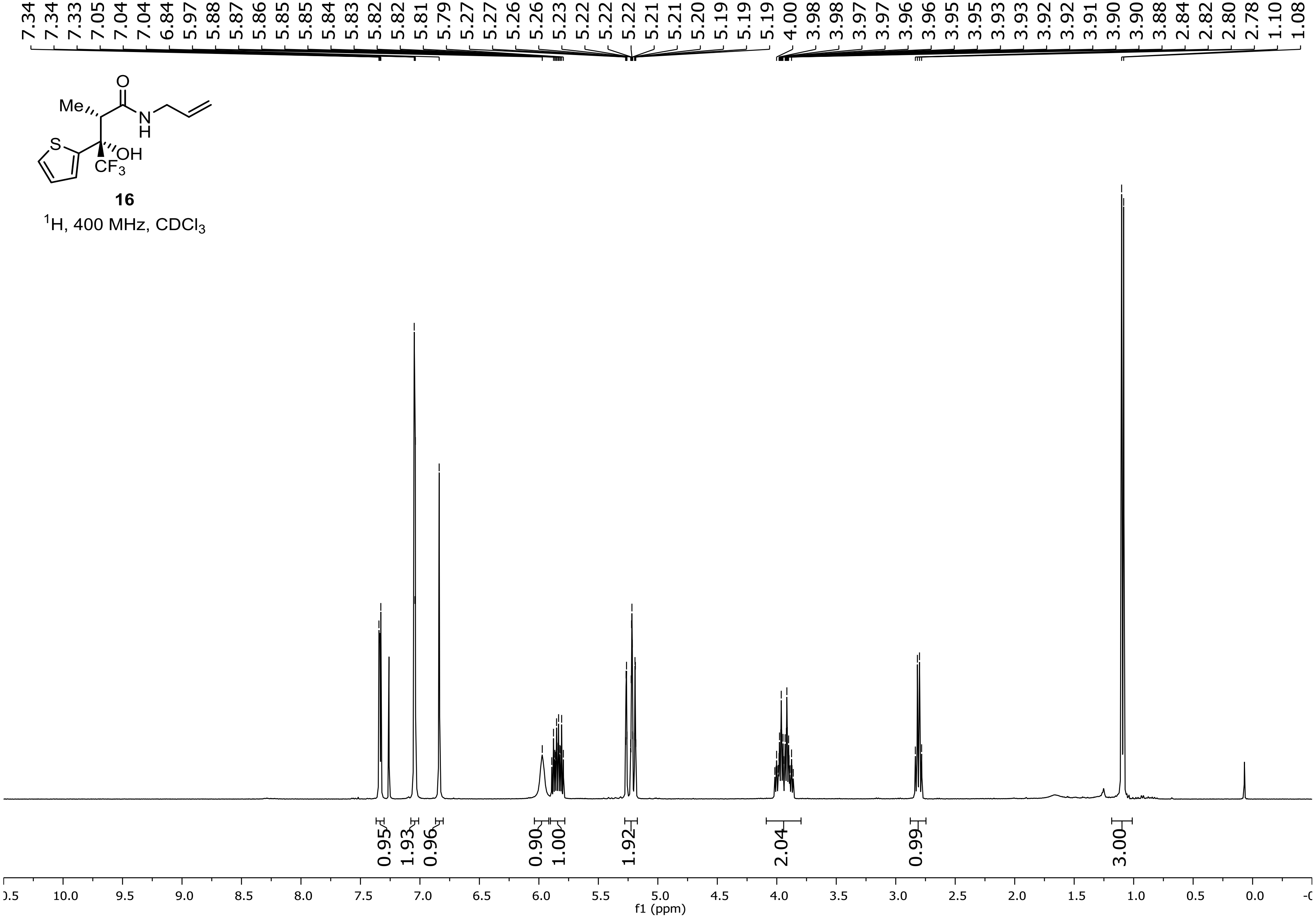
7.34 7.34 7.33 7.05 7.04 7.04 6.84 5.97 5.88 5.87 5.86 5.85 5.85 5.84 5.83 5.82 5.82 5.81 5.79 5.27 5.27 5.26 5.26 5.23 5.22 5.22 5.22 5.21 5.21 5.20 5.19 5.19 5.19 4.00 3.98 3.98 3.97 3.97 3.96 3.96 3.95 3.95 3.93 3.93 3.92 3.92 3.91 3.90 3.90 3.88 2.84 2.82 2.80 2.78 1.10 1.08
Me
O
N
H
S
OH
CF3
16
1H, 400 MHz, CDCl3
0.95
1.93
0.96
0.90
1.00
1.92
2.04
0.99
3.00
10.0 9.5 9.0 8.5 8.0 7.5 7.0 6.5 6.0 5.5 5.0 4.5 4.0 3.5 3.0 2.5 2.0 1.5 1.0 0.5 0.0
f1 (ppm)

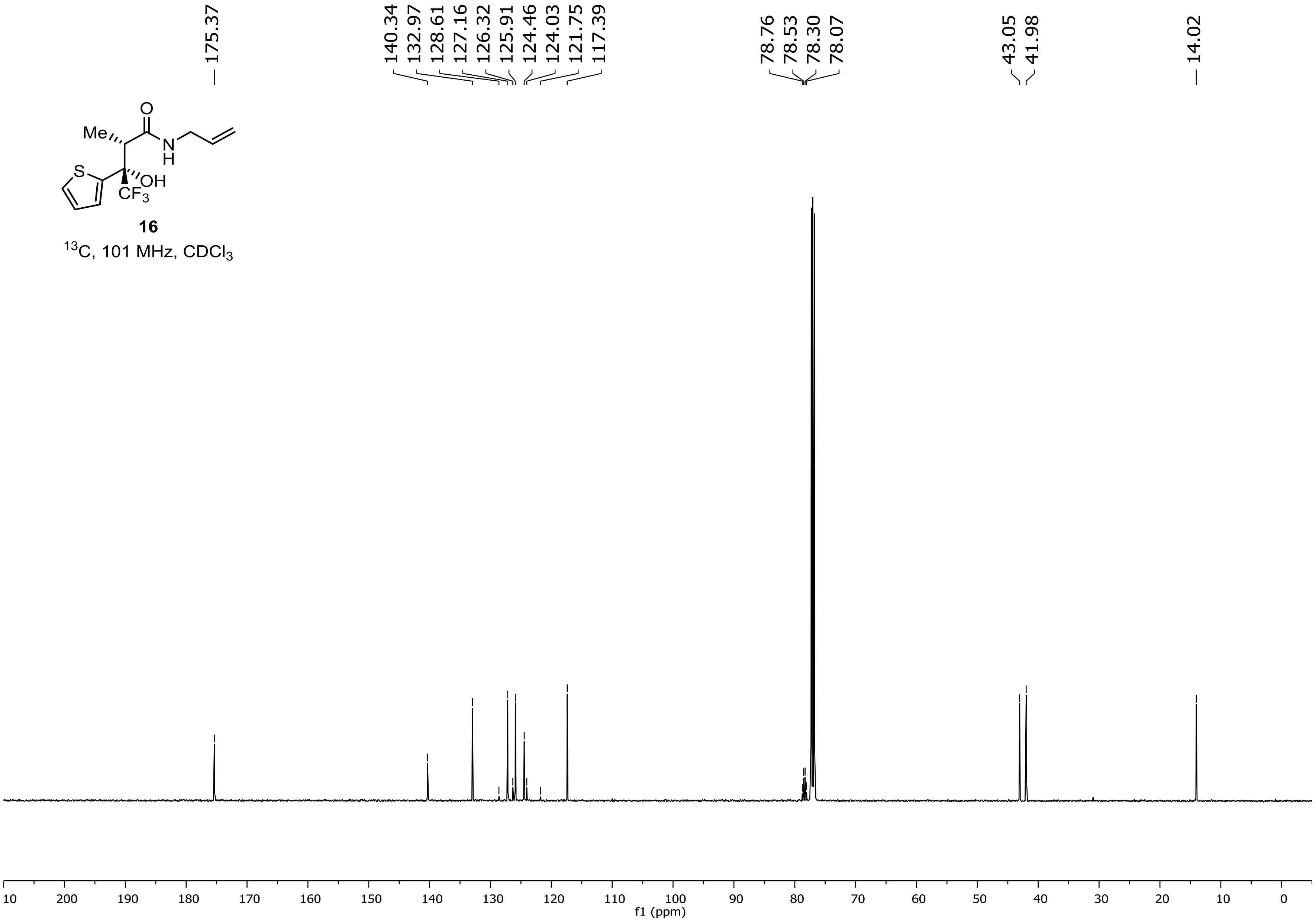
175.37
140.34
132.97
128.61
127.16
126.32
125.91
124.46
124.03
121.75
117.39
78.76
78.53
78.30
78.07
43.05
41.98
14.02
Me
O
N
H
S
OH
CF3
16
13C, 101 MHz, CDCl3
10
200
190
180
170
160
150
140
130
120
110
100
90
80
70
60
50
40
30
20
10
0
f1 (ppm)

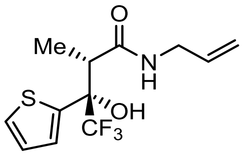

16

^{19}F, 376 MHz, $CDCl_3$

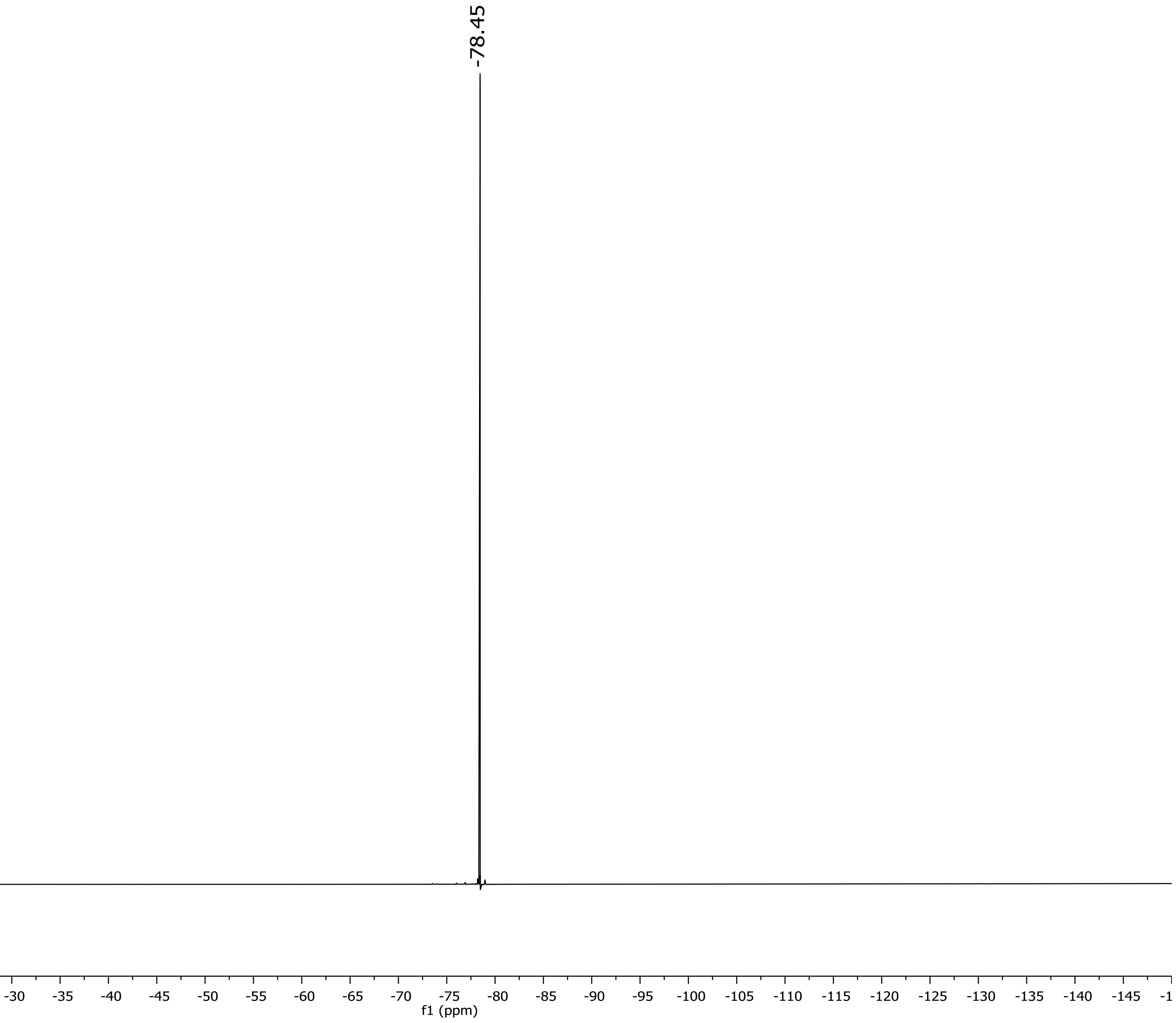

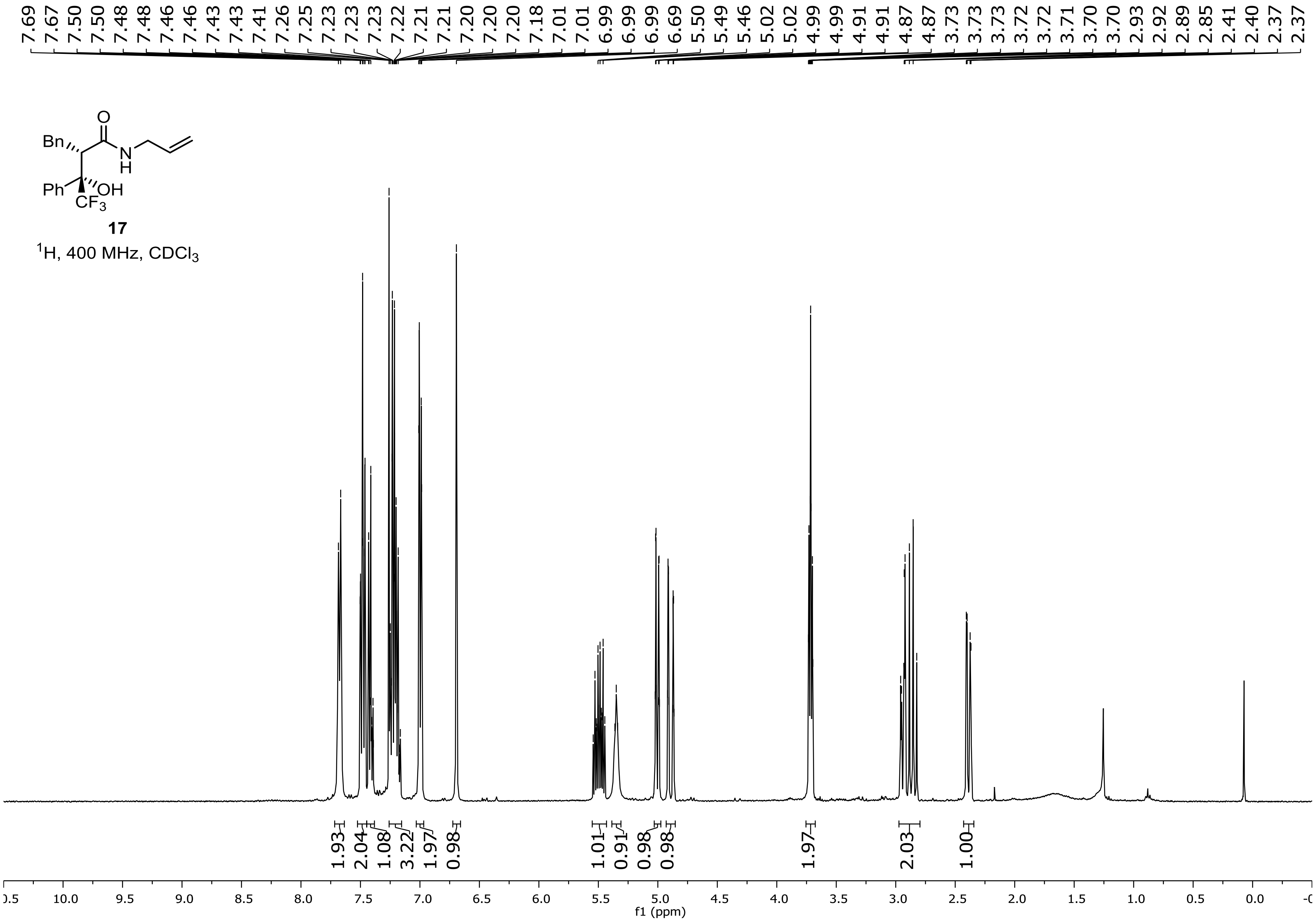

7.69 7.67 7.50 7.50 7.48 7.48 7.46 7.46 7.43 7.43 7.41 7.26 7.25 7.23 7.23 7.23 7.22 7.21 7.21 7.20 7.20 7.20 7.18 7.01 7.01 6.99 6.99 6.99 6.69 5.50 5.49 5.46 5.02 5.02 4.99 4.99 4.91 4.91 4.87 4.87 3.73 3.73 3.73 3.72 3.72 3.71 3.70 3.70 2.93 2.92 2.89 2.85 2.41 2.40 2.37 2.37
Bn
O
N
H
Ph
OH
CF_3
17
^{1}H, 400 MHz, $CDCl_3$
1.93 2.04 1.08 3.22 1.97 0.98 1.01 0.91 0.98 0.98 1.97 2.03 1.00
10.0 9.5 9.0 8.5 8.0 7.5 7.0 6.5 6.0 5.5 5.0 4.5 4.0 3.5 3.0 2.5 2.0 1.5 1.0 0.5 0.0
f1 (ppm)

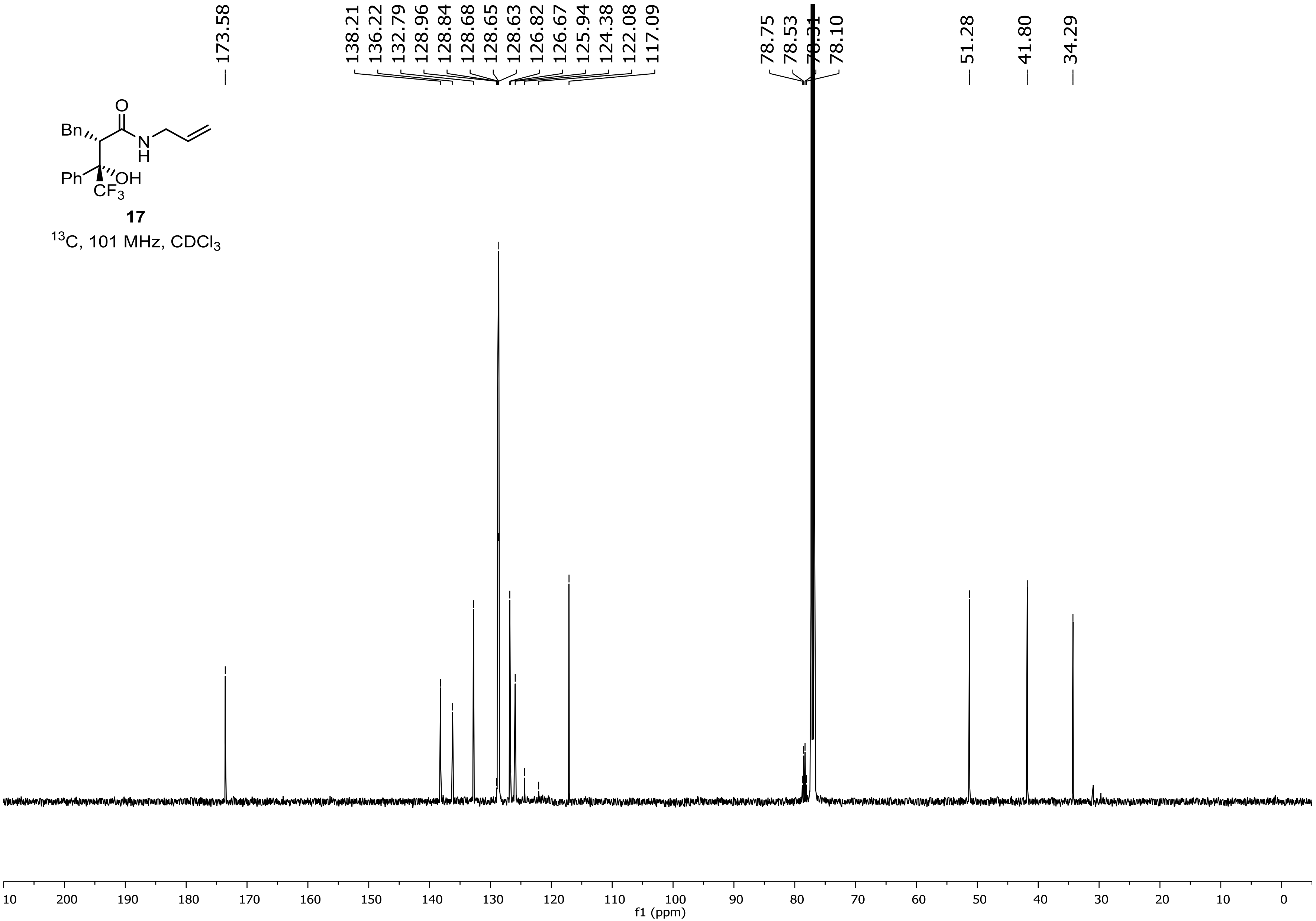

173.58
138.21
136.22
132.79
128.96
128.84
128.68
128.65
128.63
126.82
126.67
125.94
124.38
122.08
117.09
78.75
78.53
78.10
51.28
41.80
34.29
Bn
O
N
H
Ph
OH
CF3
17
^{13}C, 101 MHz, $CDCl_3$
10 200 190 180 170 160 150 140 130 120 110 100 90 80 70 60 50 40 30 20 10 0
f1 (ppm)

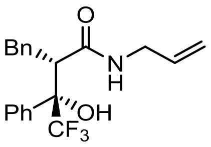

17

^{19}F, 376 MHz, $CDCl_3$

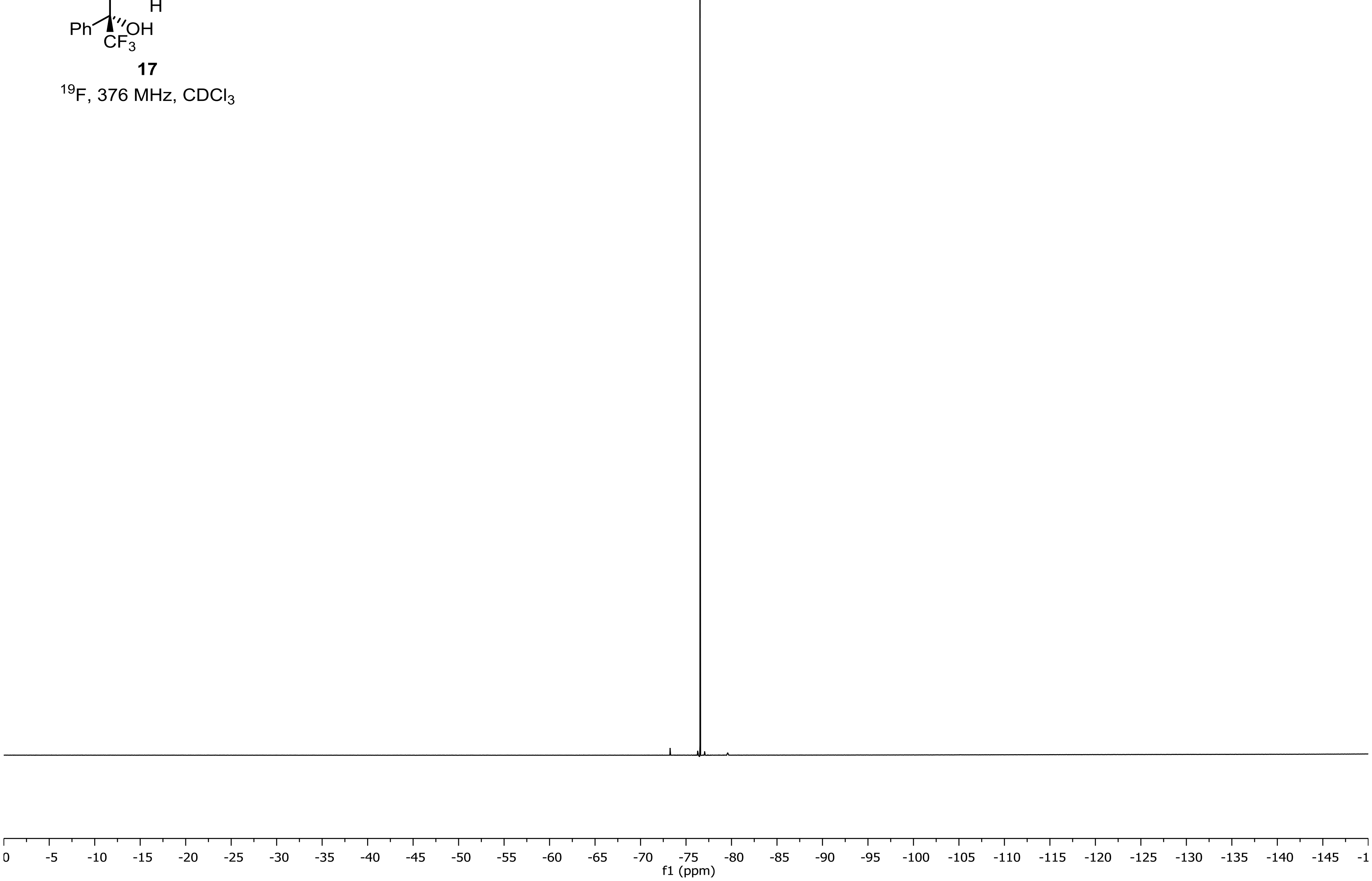

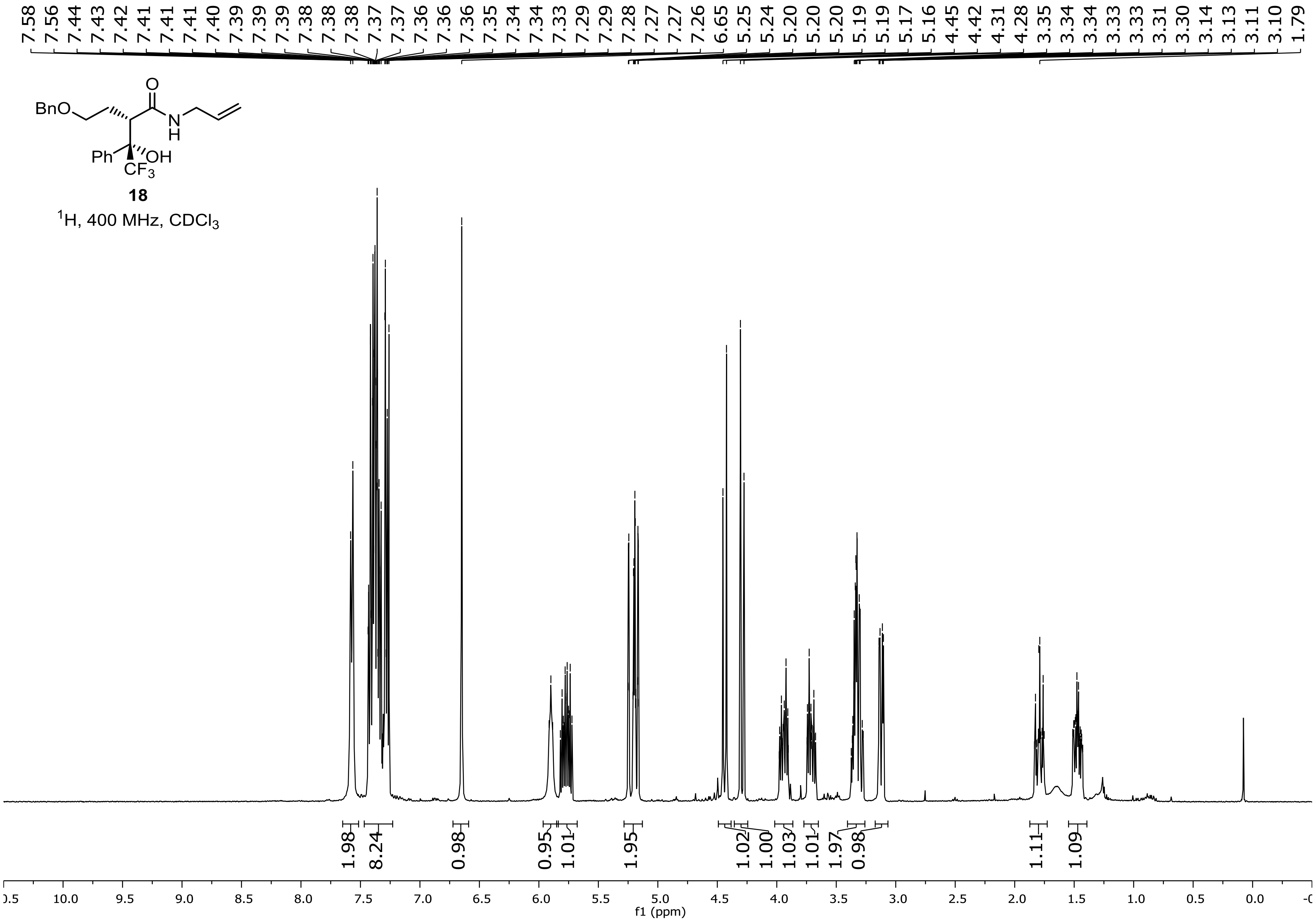
7.58 7.56 7.44 7.43 7.42 7.41 7.41 7.41 7.40 7.39 7.39 7.39 7.38 7.38 7.38 7.37 7.37 7.36 7.36 7.36 7.35 7.34 7.34 7.33 7.29 7.29 7.28 7.27 7.27 7.26 6.65 5.25 5.24 5.20 5.20 5.20 5.19 5.19 5.17 5.16 4.45 4.42 4.31 4.28 3.35 3.34 3.34 3.33 3.33 3.31 3.30 3.14 3.13 3.11 3.10 1.79
BnO
O
N
H
Ph
OH
CF_3
18
^{1}H, 400 MHz, $CDCl_3$
1.98
8.24
0.98
0.95
1.01
1.95
1.02
1.00
1.03
1.01
1.97
0.98
1.11
1.09
10.0 9.5 9.0 8.5 8.0 7.5 7.0 6.5 6.0 5.5 5.0 4.5 4.0 3.5 3.0 2.5 2.0 1.5 1.0 0.5 0.0
f1 (ppm)

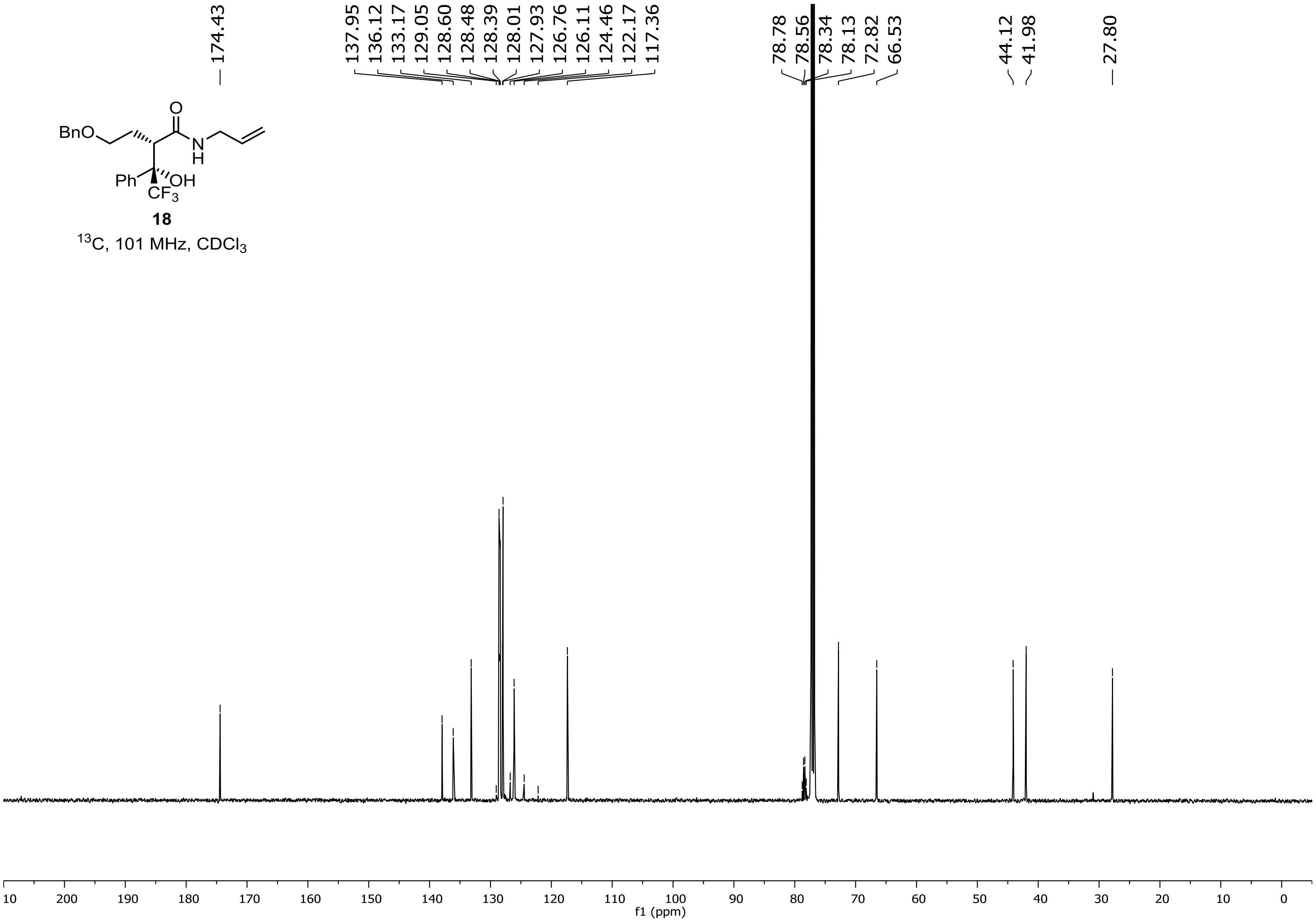

18

^{13}C, 101 MHz, $CDCl_3$

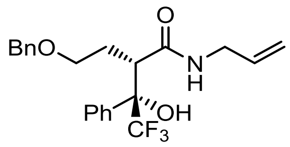

18

^{19}F, 376 MHz, $CDCl_3$

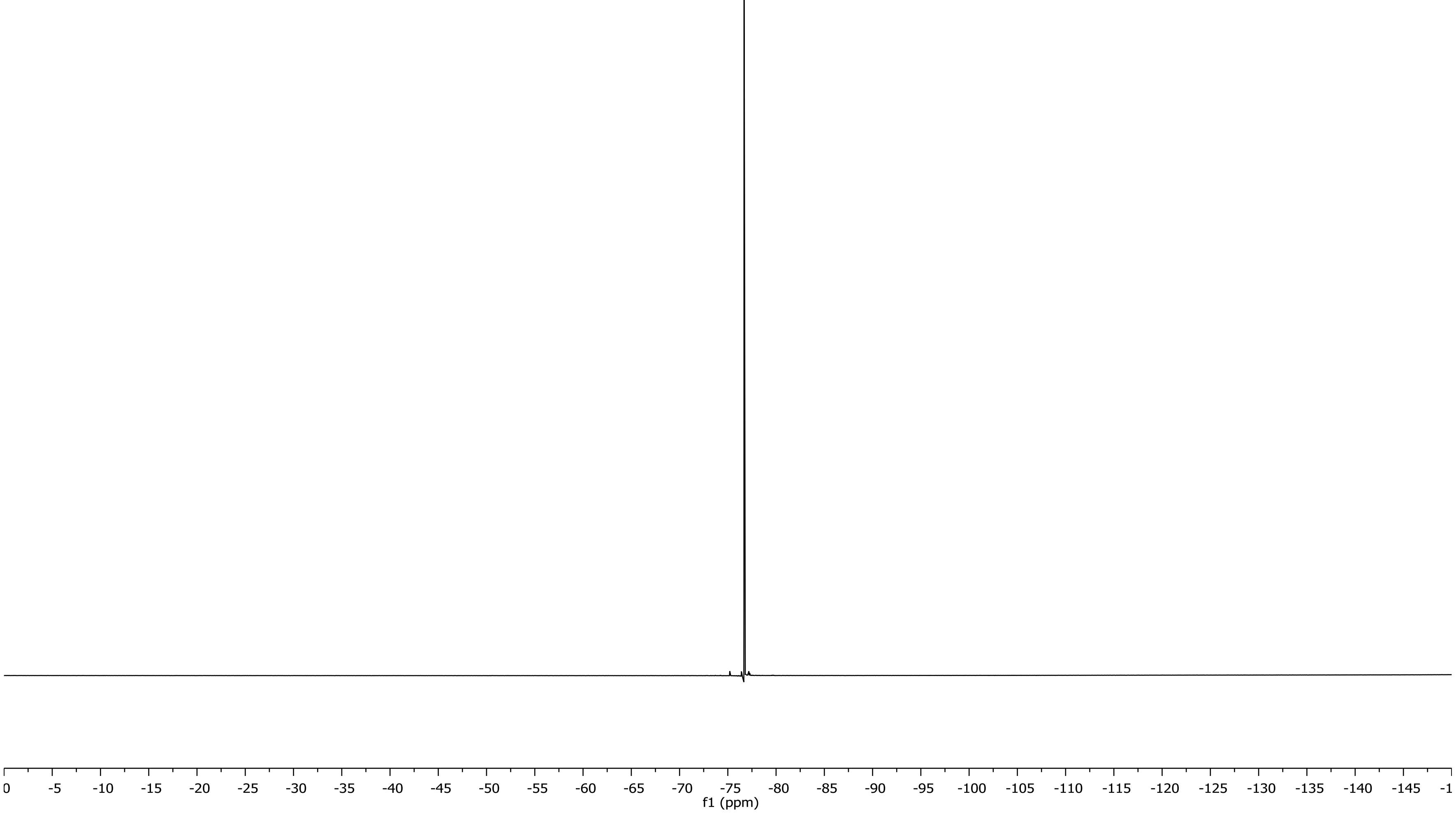

(2*S*,3*S*)-*N*-Benzyl-4,4,4-trifluoro-3-hydroxy-2-methyl-3-phenylbutanamide (8)

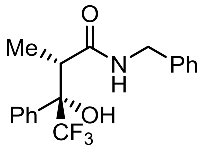

Chiral HPLC analysis; Chiralcel OD-H (95:5 hexane : 2-propanol, flow rate 1 mLmin^{-1}, 211 nm, 30 °C) t_R minor (2*R*,3*R*): 23.0 min, t_R major (2*S*,3*S*): 24.8 min, 96:4 er.

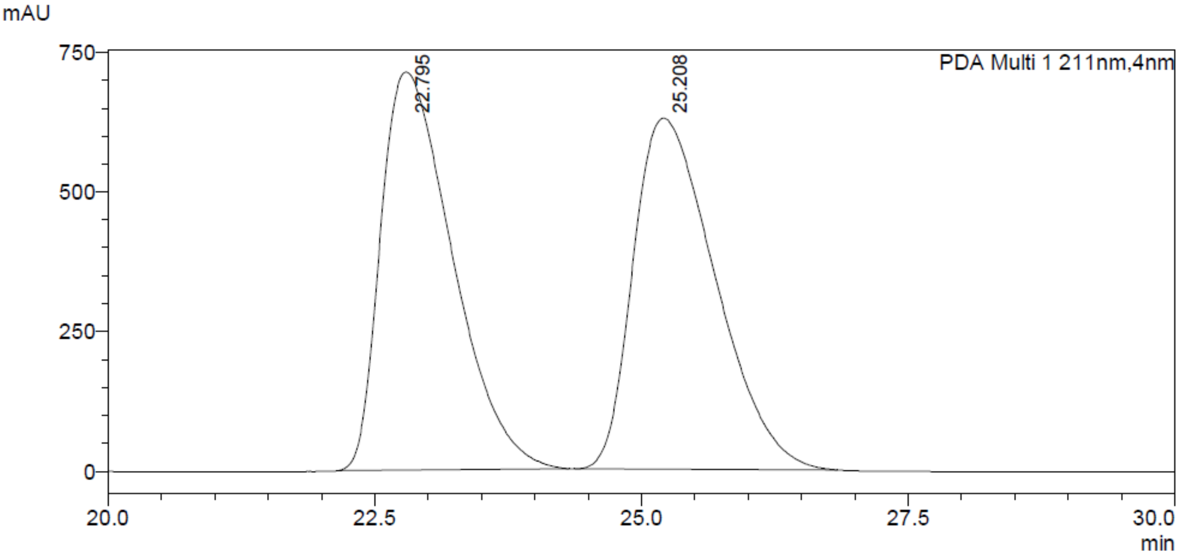

<Peak Table>

PDA Ch1 211nm

Peak#	Ret. Time	Area%
1	22.795	49.822
2	25.208	50.178
Total		100.000

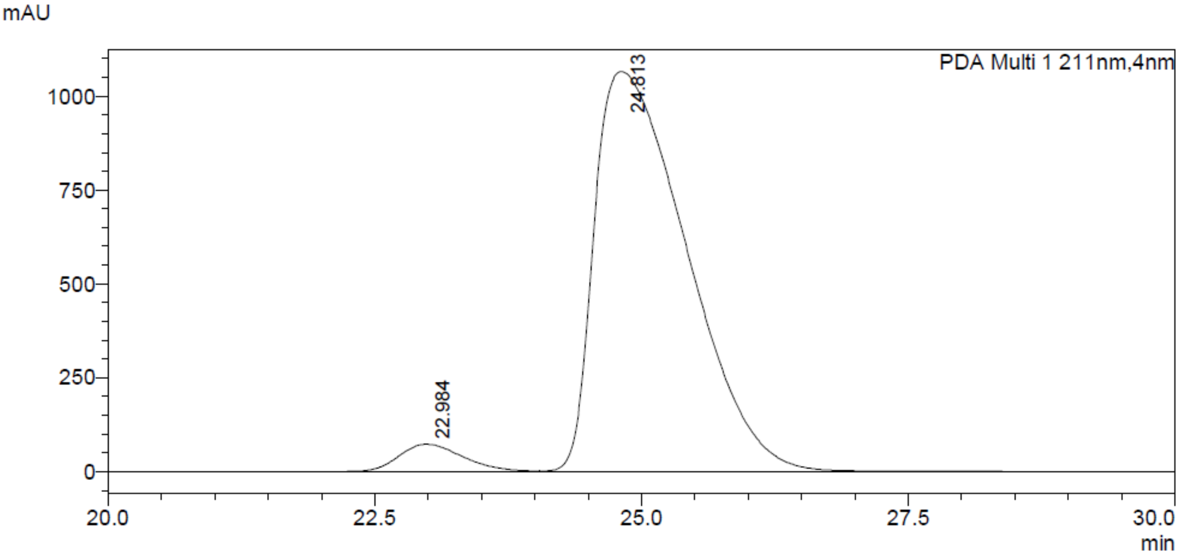

<Peak Table>

PDA Ch1 211nm

Peak#	Ret. Time	Area%
1	22.984	4.496
2	24.813	95.504
Total		100.000

(2*S*,3*S*)-4,4,4-Trifluoro-3-hydroxy-2-methyl-3-phenyl-1-(pyrrolidin-1-yl)butan-1-one (**9**)

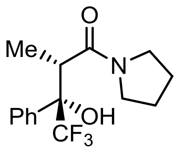

Chiral HPLC analysis; Chiralcel OD-H (90:10 hexane : 2-propanol, flow rate 1 mLmin^{-1}, 211 nm, 30 °C) t_R minor (2*R*,3*R*): 5.3 min, t_R major (2*S*,3*S*): 5.9 min, 96:4 er.

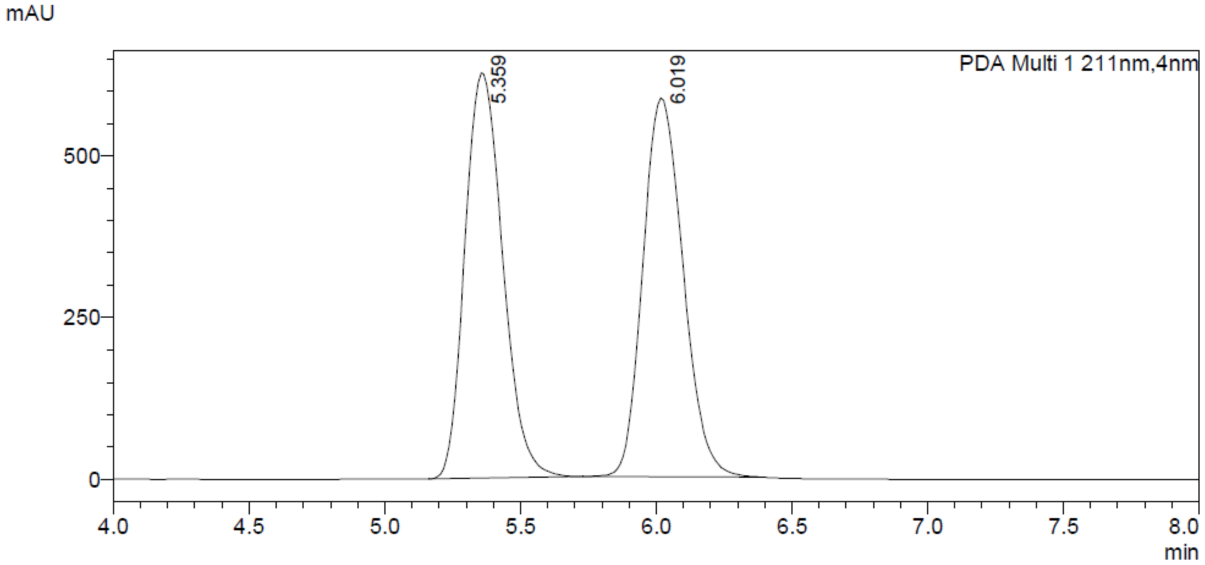

<Peak Table>

PDA Ch1 211nm

Peak#	Ret. Time	Area%
1	5.359	50.129
2	6.019	49.871
Total		100.000

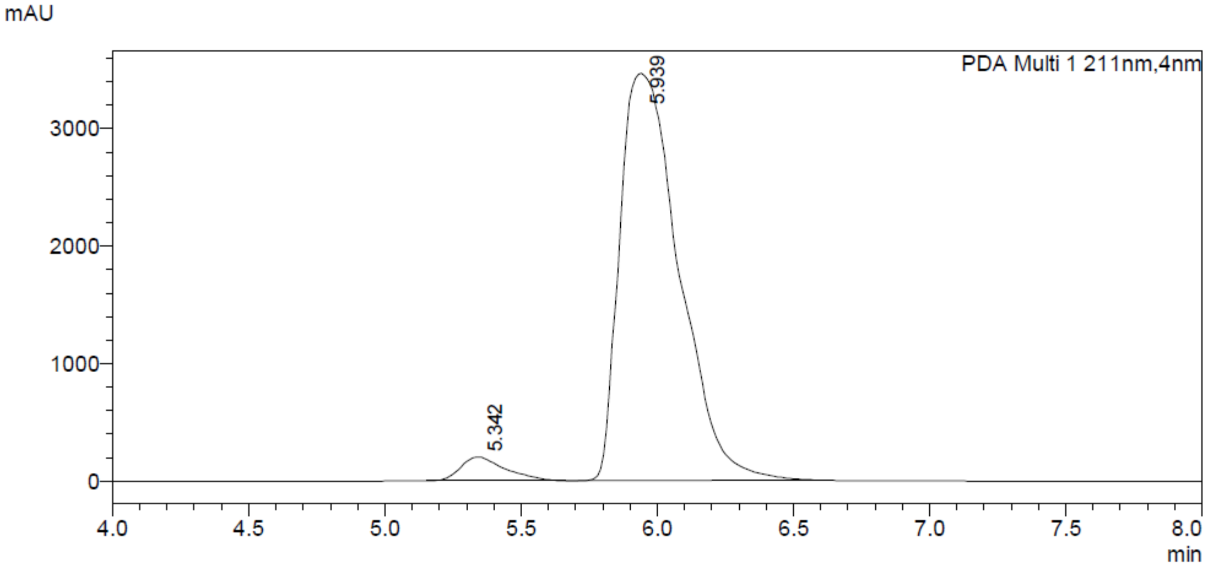

<Peak Table>

PDA Ch1 211nm

Peak#	Ret. Time	Area%
1	5.342	4.171
2	5.939	95.829
Total		100.000

(2*S*,3*S*)-4,4,4-Trifluoro-3-hydroxy-2-methyl-3-phenylbutanamide (10)

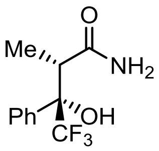

Chiral GC analysis Restek Rt®bDEXcst (length: 30 m, thickness: 0.250 mm, film thickness: 0.25 μm), carrier gas: He, linear velocity: 60 cm/sec, temperature: 160 °C, t_R minor (2*R*,3*R*) 44.0 min, t_R major (2*S*,3*S*) 45.6 min, > 99:1 er.

Peaks at 46.5 and 49.1 min in racemic trace belong to minor diastereoisomer

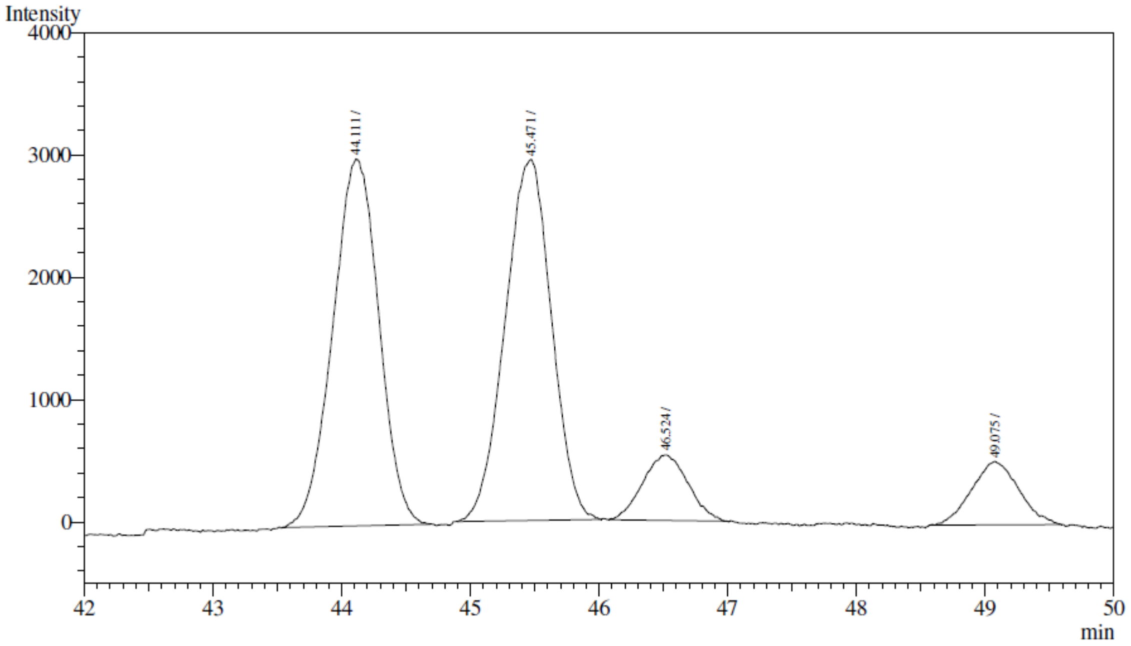

Peak#	Ret.Time	Area	Area%
1	44.111	74853	42.7773
2	45.471	73843	42.2003
3	46.524	13078	7.4740
4	49.075	13209	7.5485
Total		174983	100.0000

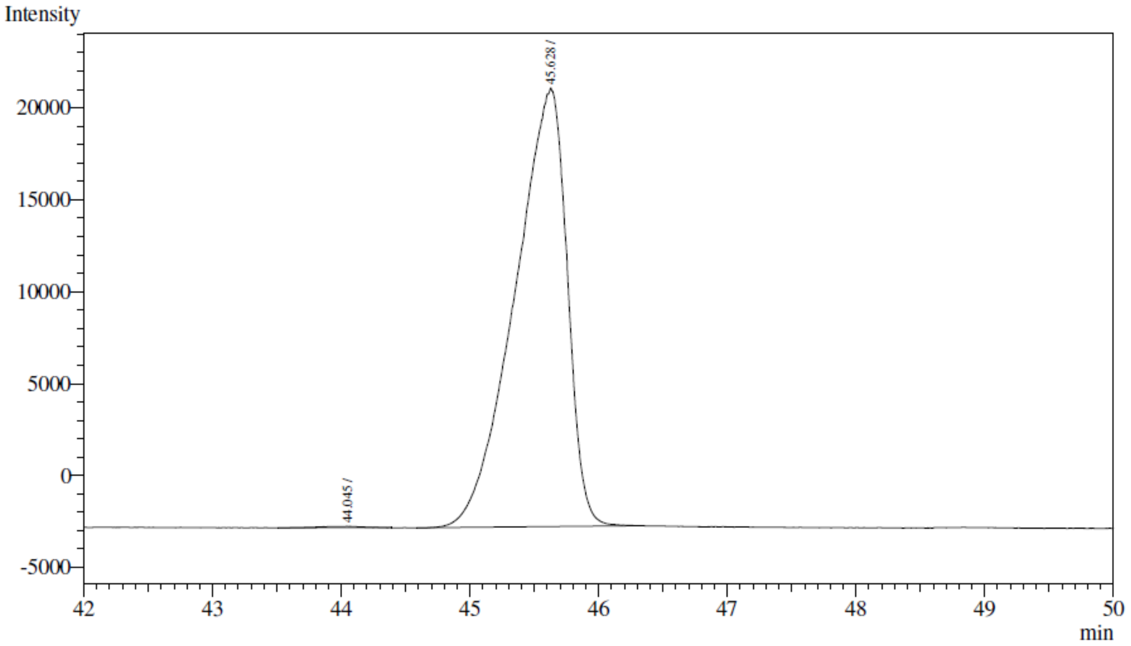

Peak#	Ret.Time	Area	Area%
1	44.045	1637	0.2424
2	45.628	673526	99.7576
Total		675163	100.0000

(2*R*,3*S*)-4,4,4-Trifluoro-2-methyl-3-phenylbutane-1,3-diol (**11**)

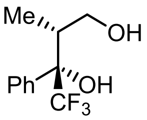

Chiral GC analysis Agilent Cyclosil-B (length: 30 m, thickness: 0.250 mm, film thickness: 0.25 μm), carrier gas: He, linear velocity: 30 cm/sec, temperature: 135 °C, t_R minor (2*S*,3*R*) 50.8 min, t_R major (2*R*,3*S*) 51.8 min, 96:4 er.

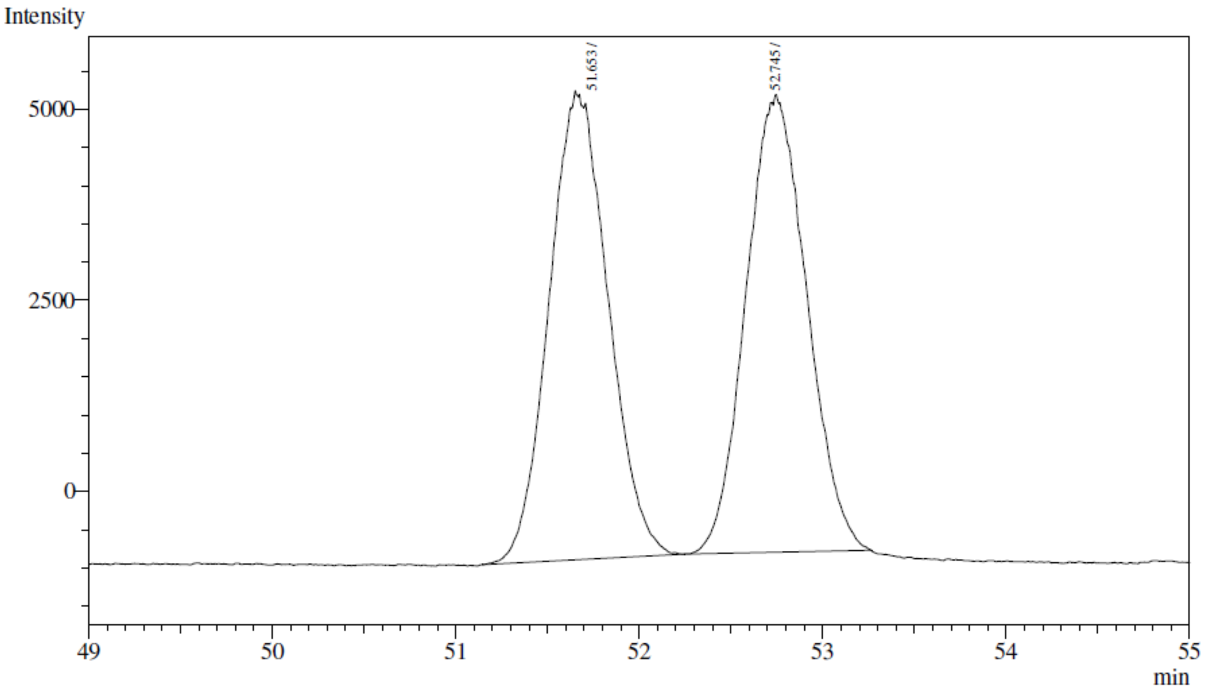

Peak#	Ret.Time	Area	Area%
1	51.653	137275	49.8653
2	52.745	138017	50.1347
Total		275292	100.0000

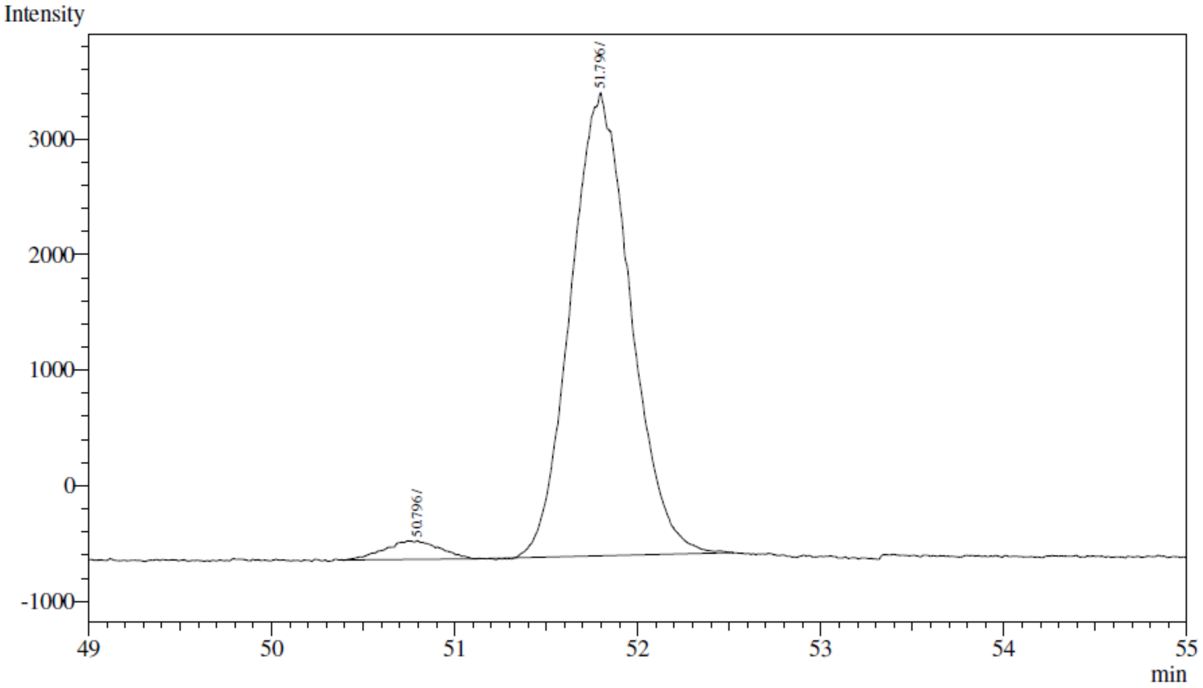

Peak#	Ret.Time	Area	Area%
1	50.796	3356	3.5473
2	51.796	91246	96.4527
Total		94602	100.0000

(2*S*,3*S*)-*N*-Allyl-3-(4-bromophenyl)-4,4,4-trifluoro-3-hydroxy-2-methylbutanamide (12)

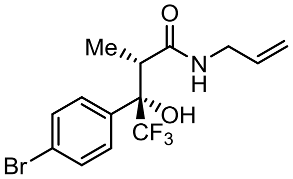

Chiral GC analysis Agilent Cyclosil-B (length: 30 m, thickness: 0.250 mm, film thickness: 0.25 μm), carrier gas: He, linear velocity: 60 cm/sec, temperature: 190 °C, t_R minor (2*R*,3*R*) 29.9 min, t_R major (2*S*,3*S*) 30.6 min, 99:1 er.

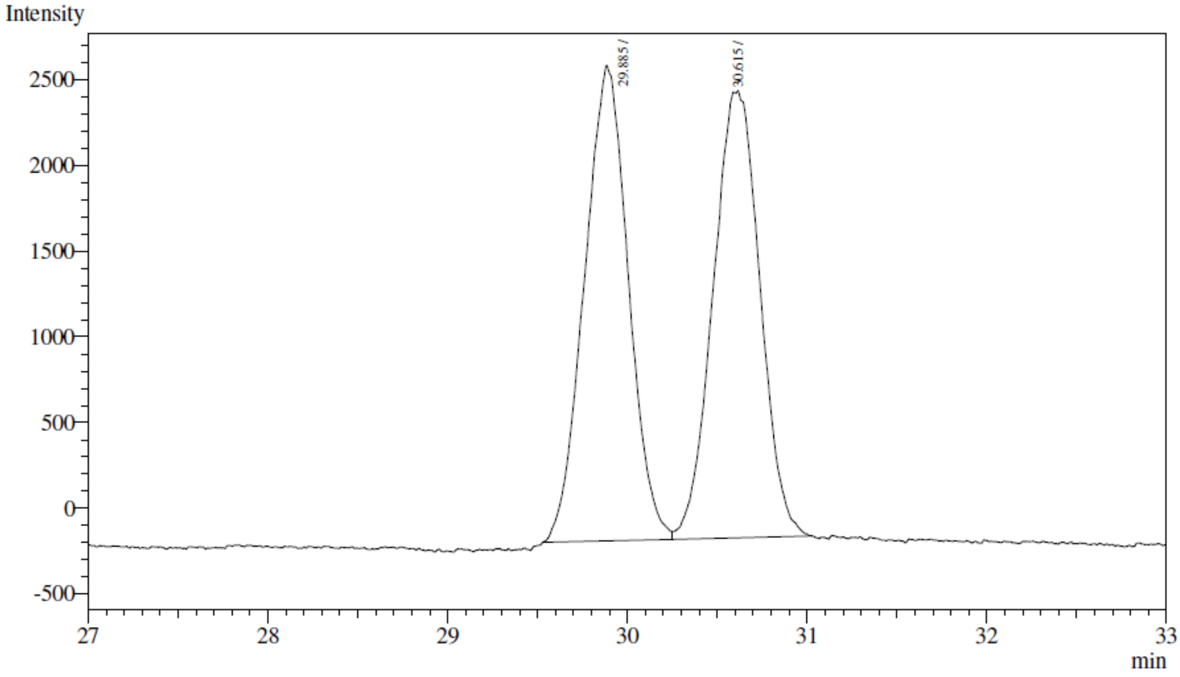

Peak#	Ret.Time	Area	Area%
1	29.885	47191	49.9343
2	30.615	47315	50.0657
**Total**		94506	100.0000

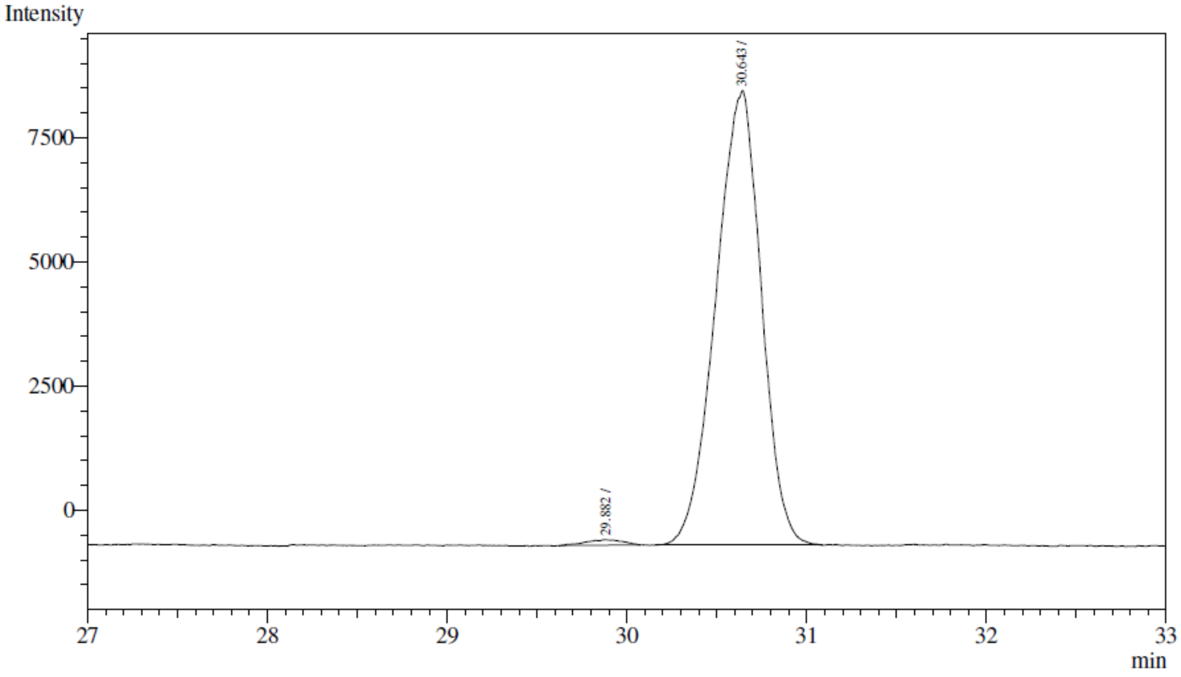

Peak#	Ret.Time	Area	Area%
1	29.882	1602	0.9866
2	30.643	160755	99.0134
**Total**		162357	100.0000

(2*S*,3*S*)-*N*-Allyl-4,4,4-trifluoro-3-(4-fluorophenyl)-3-hydroxy-2-methylbutanamide (13)

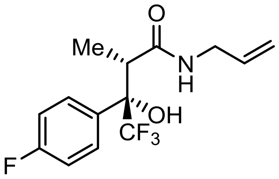

Chiral GC analysis Agilent Cyclosil-B (length: 30 m, thickness: 0.250 mm, film thickness: 0.25 µm), carrier gas: He, linear velocity: 60 cm/sec, temperature: 157 °C, t_R minor (2*R*,3*R*) 40.1 min, t_R major (2*S*,3*S*) 41.1 min, 97:3 er.

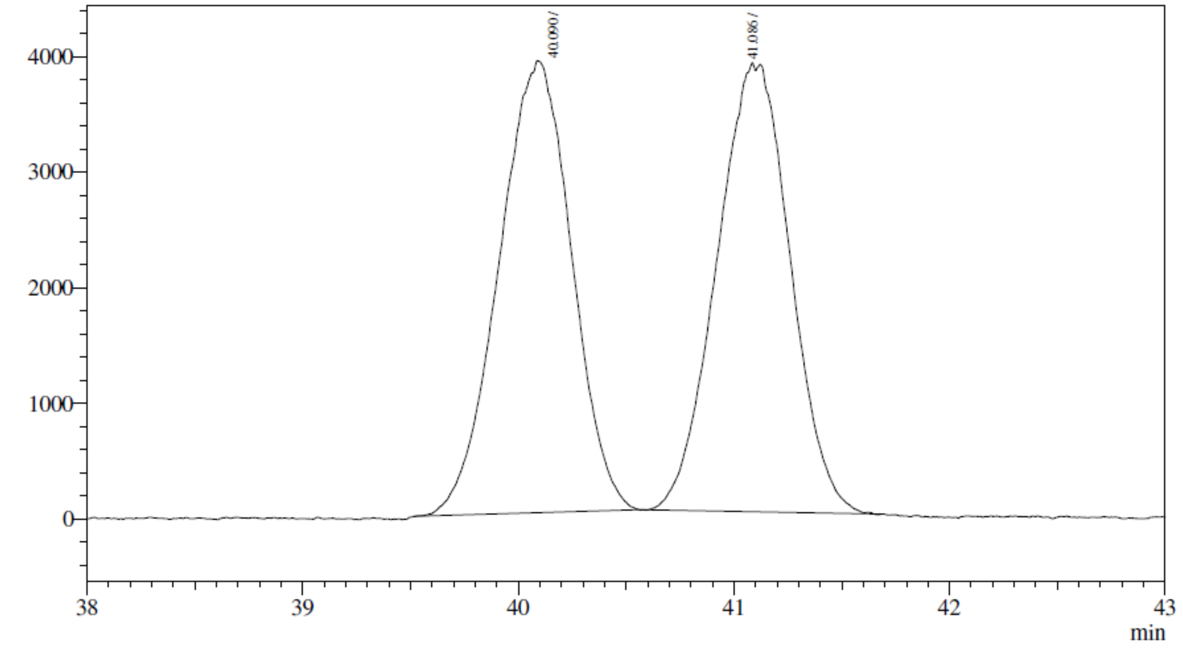

Peak#	Ret.Time	Area	Area%
1	40.090	91703	49.9090
2	41.086	92037	50.0910
Total		183740	100.0000

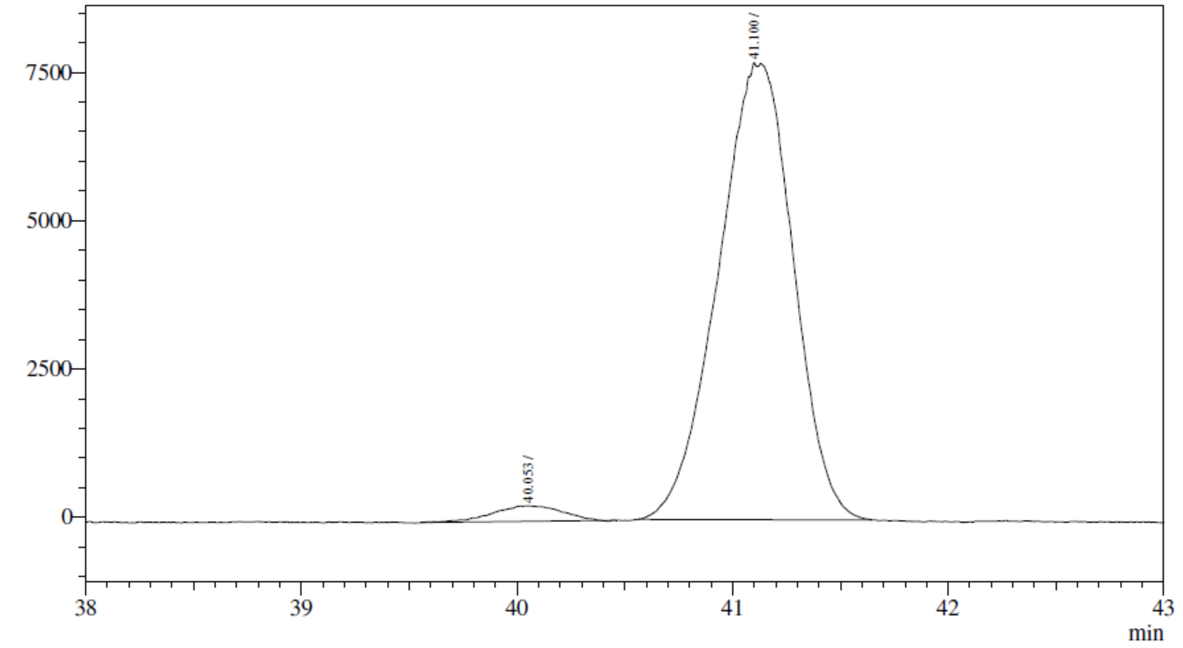

Peak#	Ret.Time	Area	Area%
1	40.053	5833	3.0318
2	41.100	186578	96.9682
Total		192411	100.0000

(2*S*,3*S*)-*N*-allyl-4,4,4-trifluoro-3-hydroxy-2-methyl-3-(4-(trifluoromethyl)phenyl)butanamide (14)

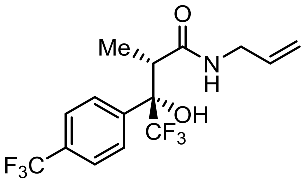

Chiral GC analysis Agilent Cyclosil-B (length: 30 m, thickness: 0.250 mm, film thickness: 0.25 μm), carrier gas: He, linear velocity: 40 cm/sec, temperature: 165 °C, t_R minor (2*R*,3*R*) 40.0 min, t_R major (2*S*,3*S*) 40.7 min, 95:5 er.

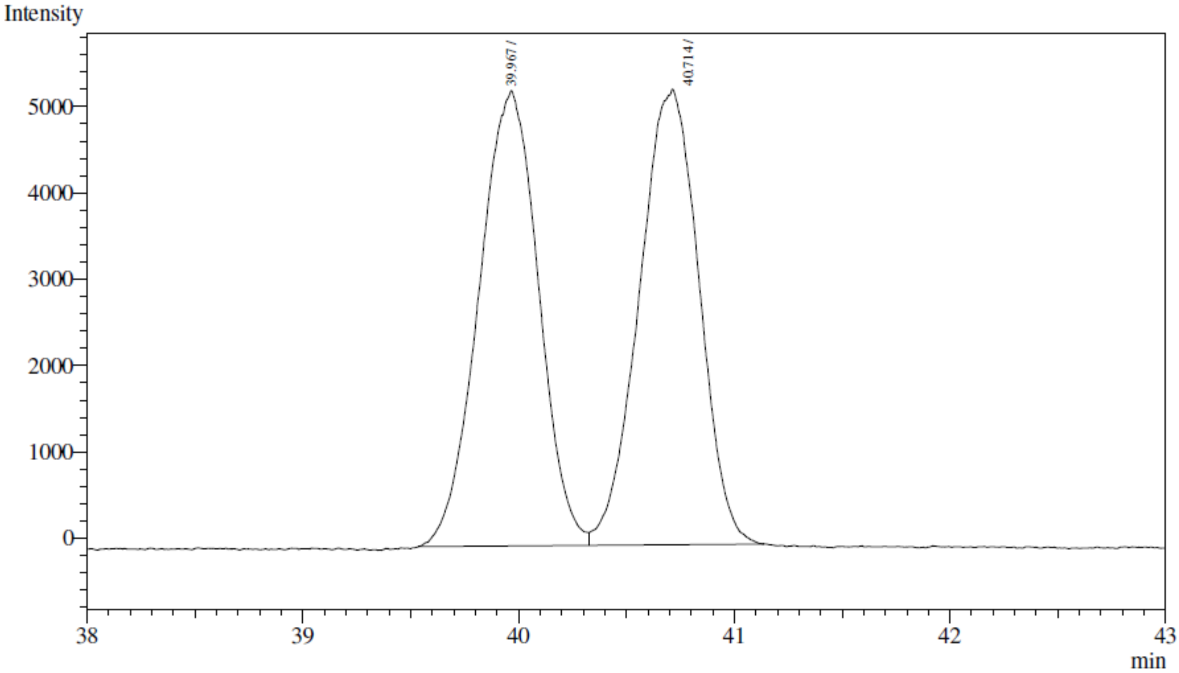

Peak#	Ret.Time	Area	Area%
1	39.967	101664	49.9536
2	40.714	101853	50.0464
**Total**		203517	100.0000

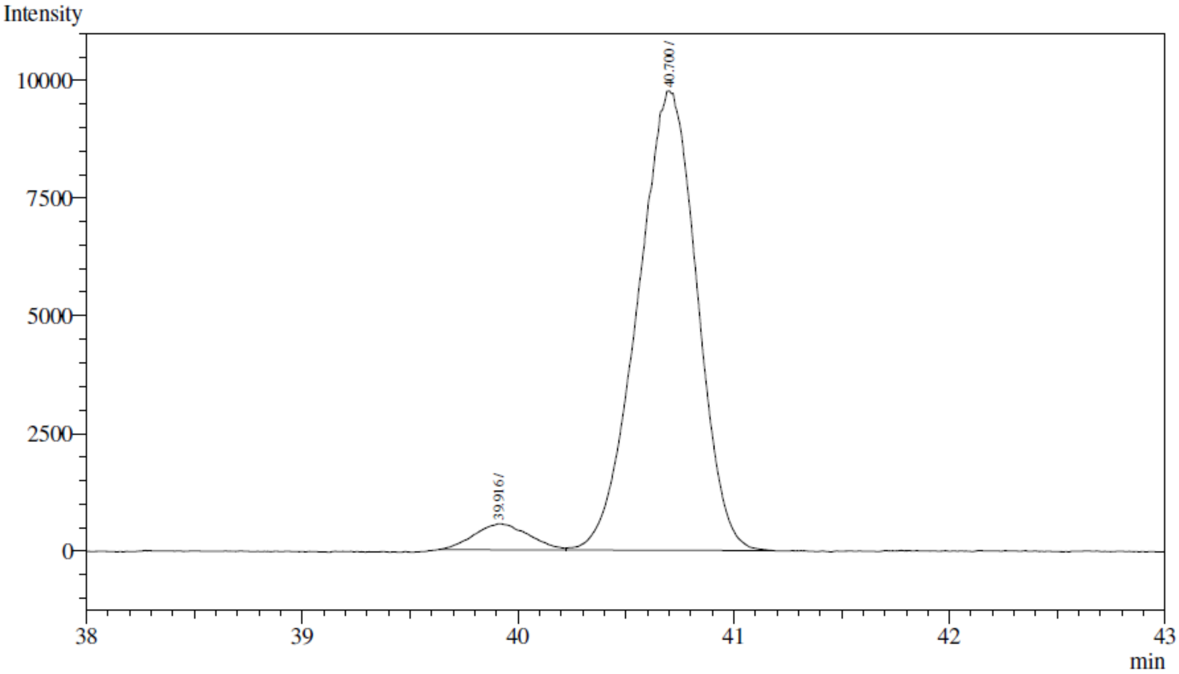

Peak#	Ret.Time	Area	Area%
1	39.916	9662	4.9148
2	40.700	186919	95.0852
**Total**		196581	100.0000

(2*S*,3*R*)-*N*-Allyl-4,4,4-trifluoro-3-hydroxy-2-methyl-3-(thiophen-2-yl)butanamide (16)

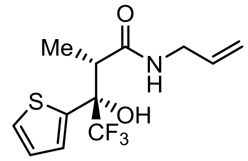

Chiral GC analysis Agilent Cyclosil-B (length: 30 m, thickness: 0.250 mm, film thickness: 0.25 μm), carrier gas: He, linear velocity: 60 cm/sec, temperature: 160 °C, t_R minor (2*R*,3*R*) 33.6 min, t_R major (2*S*,3*S*) 34.5 min, 96:4 er.

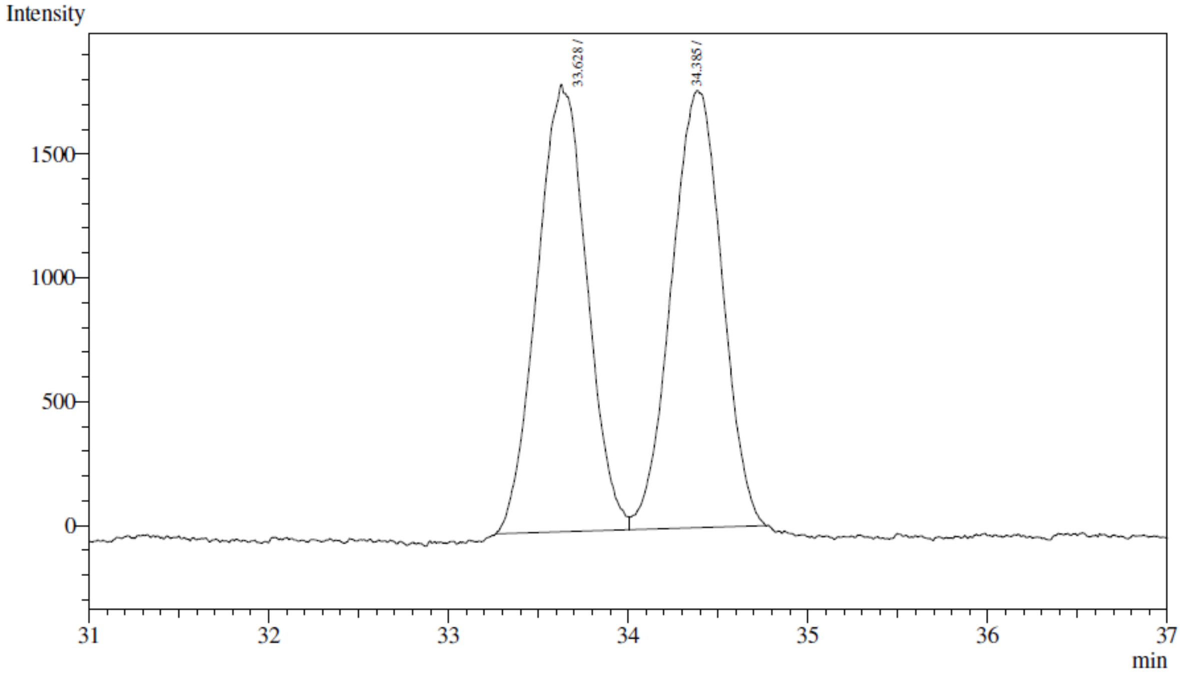

Peak#	Ret.Time	Area	Area%
1	33.628	34929	50.2018
2	34.385	34649	49.7982
Total		69578	100.0000

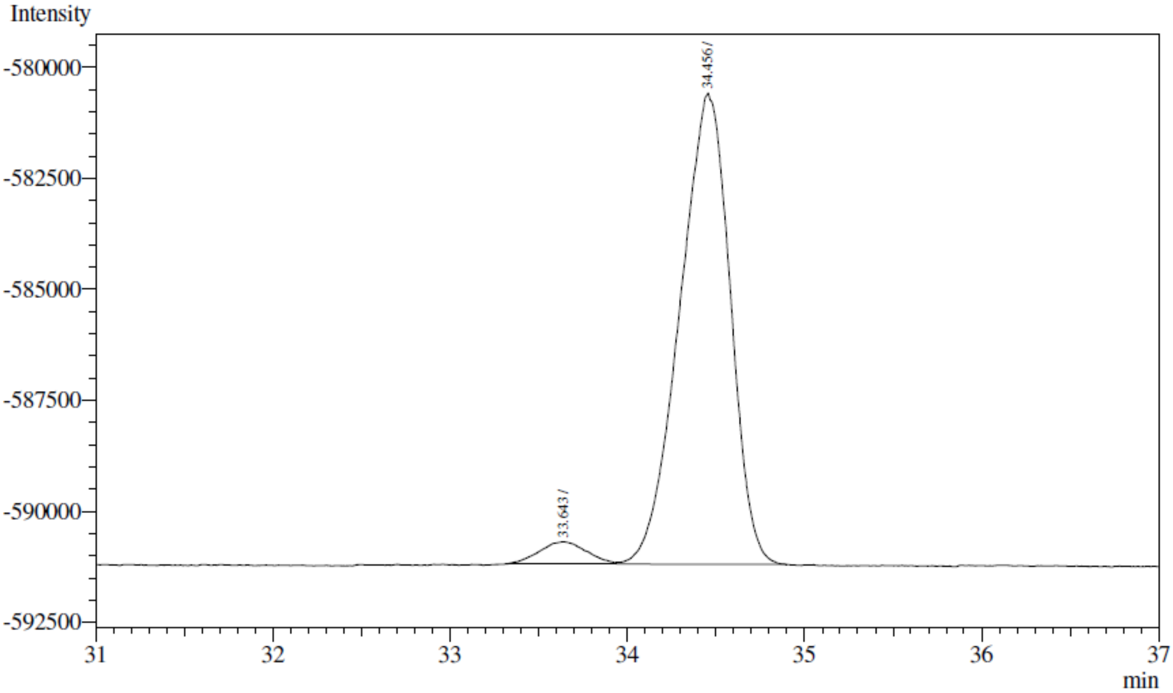

Peak#	Ret.Time	Area	Area%
1	33.643	9403	4.2968
2	34.456	209431	95.7032
Total		218834	100.0000

(2*S*,3*S*)-*N*-Allyl-2-benzyl-4,4,4-trifluoro-3-hydroxy-3-phenylbutanamide (17)

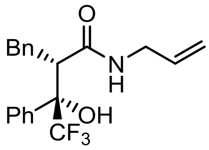

Chiral HPLC analysis; Chiralcel OD-H (95:5 hexane : 2-propanol, flow rate 1 mLmin^{-1}, 211 nm, 30 °C) t_R minor (2*R*,3*R*): 5.7 min, t_R major (2*S*,3*S*): 7.1 min, 99:1 er.

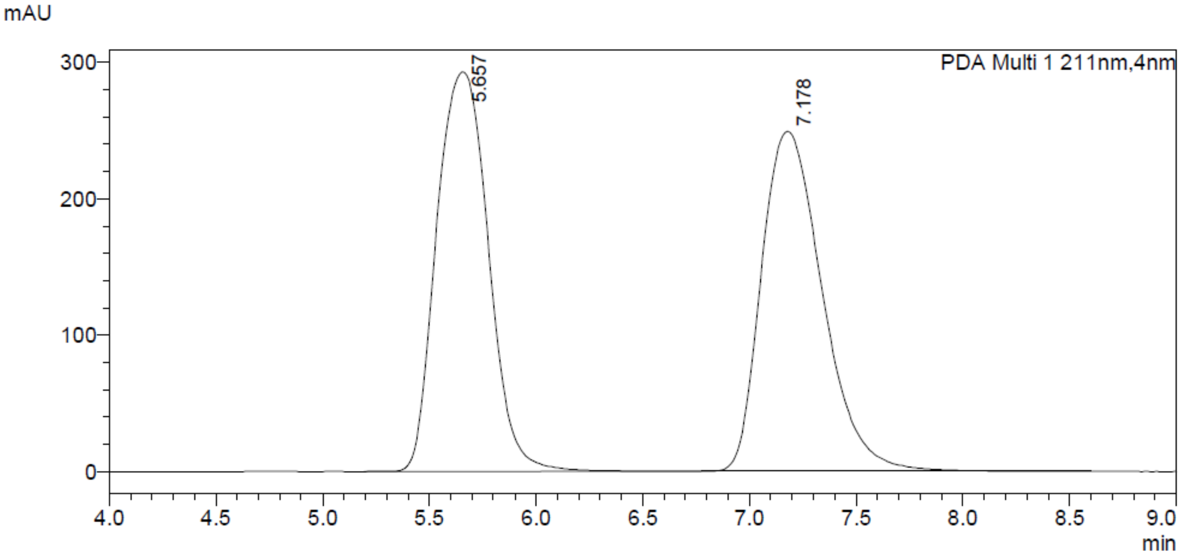

<Peak Table>

PDA Ch1 211nm

Peak#	Ret. Time	Area%
1	5.657	49.975
2	7.178	50.025
Total		100.000

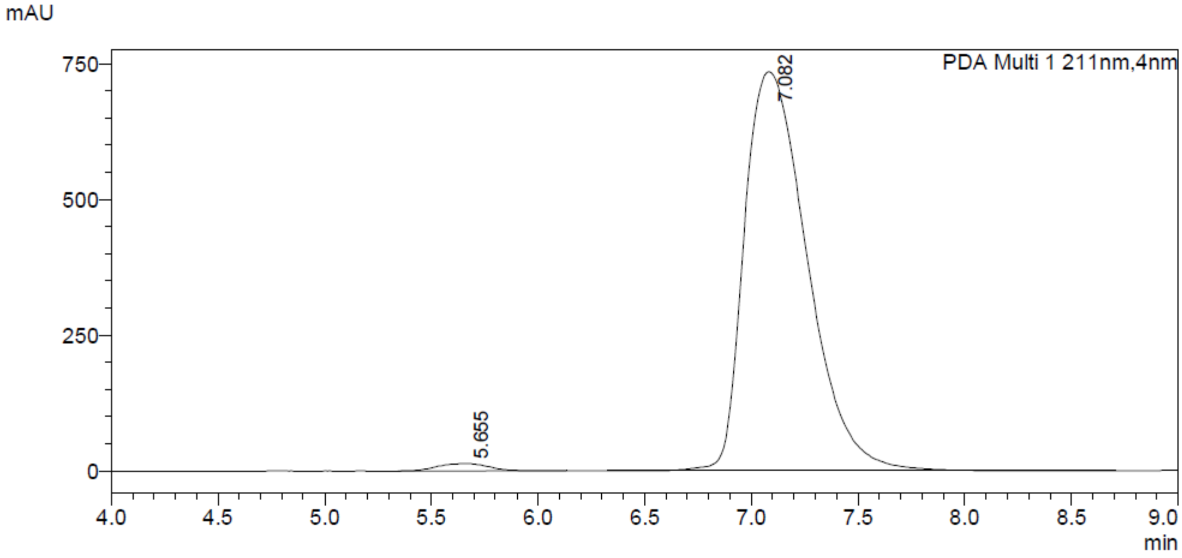

<Peak Table>

PDA Ch1 211nm

Peak#	Ret. Time	Area%
1	5.655	1.471
2	7.082	98.529
Total		100.000

(2*S*,3*S*)-*N*-Allyl-2-(2-(benzyloxy)ethyl)-4,4,4-trifluoro-3-hydroxy-3-phenylbutanamide (18)

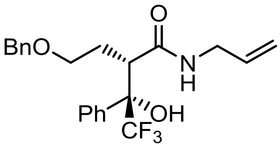

Chiral HPLC analysis; Chiralcel OD-H (95:5 hexane : 2-propanol, flow rate 1 mLmin^{-1}, 211 nm, 30 °C) t_R minor (2*R*,3*R*): 6.8 min, t_R major (2*S*,3*S*): 10.0 min, 96:4 er.

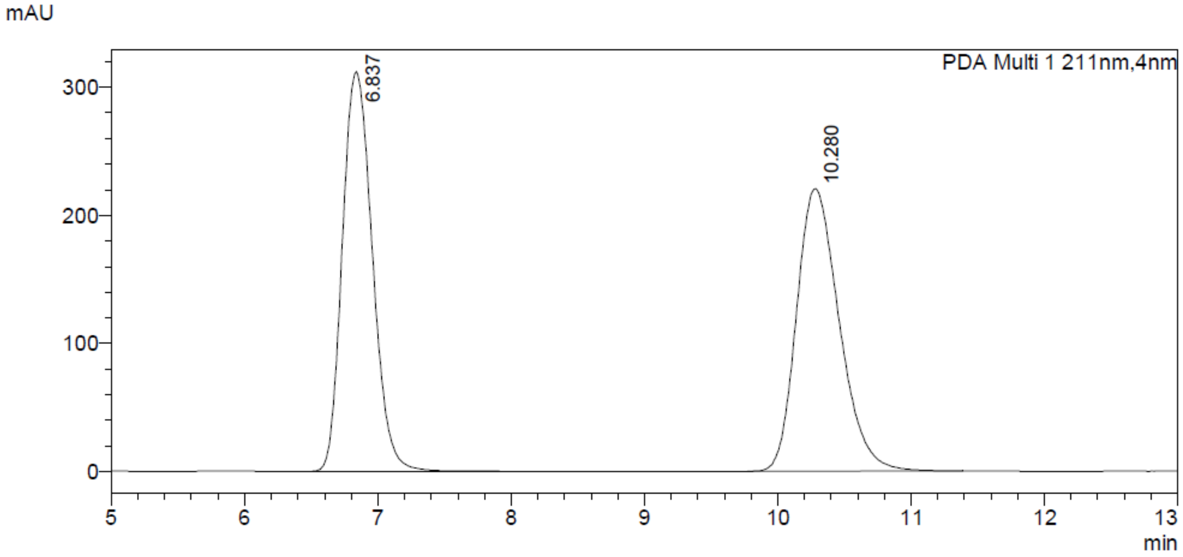

<Peak Table>

PDA Ch1 211nm

Peak#	Ret. Time	Area%
1	6.837	49.859
2	10.280	50.141
Total		100.000

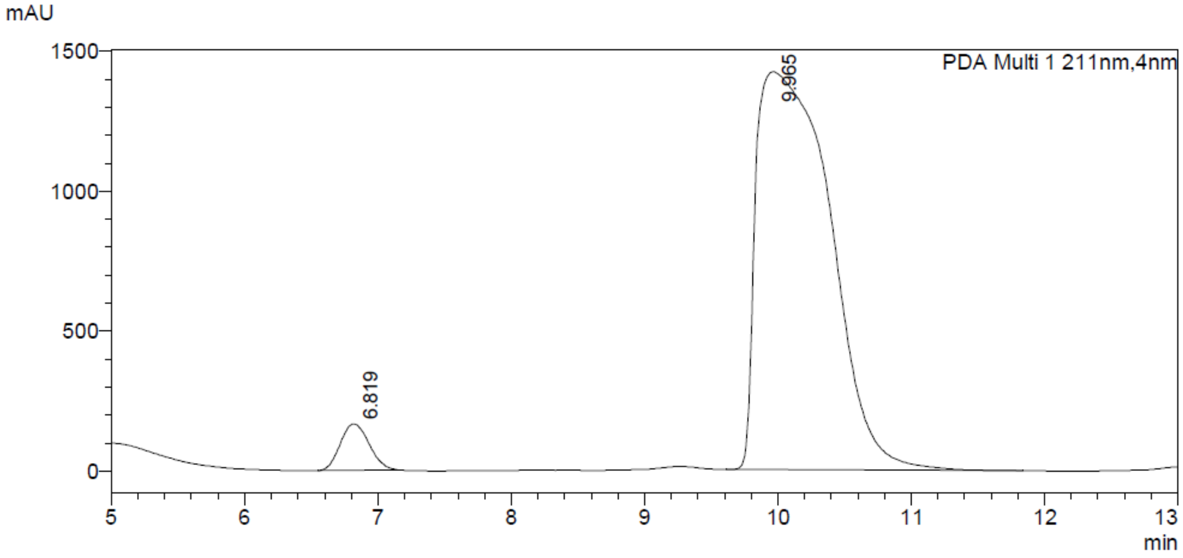

<Peak Table>

PDA Ch1 211nm

Peak#	Ret. Time	Area%
1	6.819	4.365
2	9.965	95.635
Total		100.000

References

[1] (a) Struble J. R.; Bode, J. W. *Org. Synth.* **2010**, *87*, 362–376; (b) Vora, H. U.; Lathrop, S. P.; Reynolds, N. T.; Kerr, M. S.; Read de Alaniz J.; Rovis, T. *Org. Synth.* **2010**, *87*, 350–361.

[2] Davies, A. T.; Taylor, J. E.; Douglas, J.; Collett, C. J.; Morrill, L. C.; Fallan, C.; Slawin, A. M. Z.; Churchill G.; Smith, A. D. *J. Org. Chem.* **2013**, *78*, 9243–9257.

[3] Davies, A. T.; Slawin, A. M. Z.; Smith, A. D. *Chem. Eur. J.* **2015**, *21*, 18944–18948.
